# The Interpersonal ELIZA Effect: a dyadic account of persistent over-attribution of understanding

**DOI:** 10.3389/fpsyg.2026.1869060

**Published:** 2026-10-21

**Authors:** Ashok Riehm

**Affiliations:** Independent Researcher, Hasenmoor, Germany

**Keywords:** attachment insecurity, companion chatbots, complex PTSD, demonstrated understanding, ELIZA effect, empathic accuracy, felt understanding, perceived partner responsiveness

## Abstract

Weizenbaum’s ELIZA returned users’ own words as questions and was credited with an understanding it did not have. I develop the Interpersonal ELIZA Effect (IEE) as a dyadic configuration in close relationships and with conversational agents. The responder side rests on demonstrated understanding, what a responder puts on record about the perceiver’s states in a form that can be checked against the perceiver’s own account; self-disclosure enters as a covariate of discoverability. The responder condition is one criterion with two ways of failing it: the responder produces determinate content elsewhere, on matters as intimate as the perceiver’s own reports, while setting determinate attributions about the perceiver below a specified threshold, or an attribution explicitly and literally disconfirmed is re-asserted in its uncorrected form on later, independent occasions. Uptake is persistence across occasions, not echo in the next turn; ELIZA replayed transformed inputs without modeling the user. The account joins two conditions of different kinds. The responder condition is decided: it is coded from recorded interaction and a dyad meets it, fails to meet it, or is not classified. Perceiver readiness is graded, elevated where attachment insecurity or complex post-traumatic stress lowers the threshold for counting a contribution as understanding, and is never cut into groups. A standing surplus of felt over demonstrated understanding is predicted rather than definitional: dyads can meet the responder condition at high readiness and fail to show the surplus, and, once the probe has met its evidential standard, the account is then wrong. Both are scored *in vivo* on one correspondence metric through an adaptation of the empathic accuracy paradigm. The proposed self-report instrument measures readiness and records current appraisal; it does not measure the discrepancy, which the configuration conceals from the perceiver. Five predictions follow, specified across short-term analog designs and dyadic longitudinal data. The claim is about maintenance: the surplus does not decline across the waves that follow a dyad’s meeting the responder condition; the growing share of recycled material is a secondary prediction. Path dependence, divergence by the order in which accuracy was first demanded, is a further conjecture.

## 1 Introduction

People sometimes describe romantic relationships in which they felt, over an extended period, that they had been deeply understood, only to discover weeks or months later that the partner had never grasped who they were. The conversations had felt substantive and the recognition mutual; the partner had repeated the right words, mirrored the right values, and appeared to anticipate emotional needs. What had been experienced as resonance turned out to have originated in one direction only, sustained by minimal cues from the other.

Clinicians working with people who have left a partner of the kind just described, or a chronically unavailable one, meet this account regularly, and parts of it are recognized in the literatures on traumatic bonding (Dutton and Painter, 1981; Carnes, 1997) and narcissistic mirroring (Kohut, 1971). What those accounts leave unspecified is the condition on the other side of the dyad. A perceiver’s belief that he has been understood is ordinarily disciplined by what his partner puts on record about him, in attributions determinate enough to be wrong, put to him, revised when they miss, and held in revised form afterward. Where that sequence never completes, the belief has nothing to calibrate it. The partner of the opening account, who repeated the right words and mirrored the right values, set nothing about the perceiver in that form, and the criterion of Section 3.1 reaches him only where he shows elsewhere that he can set such content, or where an attribution he did set was corrected and set again; a partner who is sparse with everyone is left unclassified.

The most discussed case of a belief left uncalibrated came from a machine. Weizenbaum (1966) published ELIZA, a natural-language program whose psychotherapy script reformulated user input as therapist-style questions. He reported that users treated it as though it had insight and concern and were reluctant to accept that it understood nothing (Weizenbaum, 1976); Section 2.1 takes up the standing of that report. What the qualification leaves standing is what Hofstadter (1995) named the ELIZA effect: people read far more understanding into strings of symbols than the symbols themselves warrant.

The perceiver side of the interpersonal case already has a literature: research on accuracy and bias in close relationships has measured the gap between what a partner is credited with and what he supplies, and has decomposed it (Ickes, 1993; Fletcher and Kerr, 2010; West and Kenny, 2011). This article begins one step further on, at the condition under which such a gap stops closing; that condition sits with the responder, it can be scored from recorded interaction, and the claim built on it is a claim about maintenance.

I propose the Interpersonal ELIZA Effect (IEE) as the name of the dyadic configuration the account holds responsible for that gap: the responder condition set out below, taken together with a level of perceiver readiness. The contribution is fourfold: the responder condition is specified without reference to self-disclosure, as one criterion with two ways of failing it, and is scored from recorded interaction (Section 3.1); I formalize the loop through which the configuration sustains itself and add path dependence as a conjecture the loop does not entail; I adopt the empathic accuracy paradigm (Ickes, 1993) as the in-vivo measurement pathway, leaving the self-report instrument the narrower role of measuring readiness and tracking change; and I make a discriminant prediction against the projection account of perceived responsiveness (Lemay et al., 2007) that turns on updating after defined disconfirming information.

## 2 Conceptual background

Four literatures carry the argument: the ELIZA effect in human-machine interaction (Section 2.1), anthropomorphism as its adaptive grounding (Section 2.2), the clinical accounts of mentalisation and projection and the gap they leave (Sections 2.3 and 2.4), and the empathic accuracy paradigm from which the measurement is taken (Section 2.5).

### 2.1 The classical ELIZA effect: Weizenbaum, Hofstadter, and the cognitive surprise of 1966

Weizenbaum’s (1966) ELIZA was, by its author’s later account (Weizenbaum, 1976), an exercise in demonstrating limits. Its DOCTOR script simulated a Rogerian psychotherapist, and pattern matching and substitution were the entirety of its method: the program held no model of meaning and no representation of the user, and stored only transformed user input.

Two properties of that design carry the argument of Section 3.1, and neither is the program’s reserve. Its returns put nothing on record about the person typing. The second property is not an absence of memory. A reserved keyword pushed a transformed copy of an input onto a stack, from which an input without keywords could later be answered with a line beginning EARLIER YOU SAID YOUR (Weizenbaum, 1966, p. 41). What outlasted the exchange was the user’s own sentence and never an attribution about him, and Weizenbaum drew the consequence himself: the test of understanding is not the ability to continue a conversation but to draw valid conclusions from what one is told, for which a program must at least be able to store selected parts of its inputs, the problem being more than one of storage (p. 43).

Weizenbaum did not anticipate the response. Users, his secretary among them, treated the program as though it had inner life and disclosed personal material to it. The source is his own (1976) retrospective report, not an observational record, and historical work has revisited elements of the standard account (Natale, 2019, 2021; Dillon, 2020; Lane et al., 2025). The episode is the origin of a question here and no evidence for an answer.

Hofstadter (1995, p. 157) named the disposition the ELIZA effect, the susceptibility to read far more comprehension into strings of symbols than the symbols themselves warrant. The Media Equation extended it (Reeves and Nass, 1996), Nass and Moon (2000) found social scripts applied mindlessly to entities that minimally meet their cues, and Shanahan (2024) sets out the vocabulary the responder side now needs; that the susceptibility on the perceiver side has not moved with it is my own reading and no finding of his.

### 2.2 Over-attribution as an asymmetric-cost default

Epley et al. (2007) hold that anthropomorphism is governed by the accessibility of human-related knowledge, by effectance motivation and by sociality motivation. Sociality rests on the narrowest experimental base of the three (Epley et al., 2008), and a later replication supported it in attenuated form (Bartz et al., 2016); it is carried here as a qualified finding.

The reason the default runs at all lies in decision costs. Where a judgment is made repeatedly under uncertainty and its two errors carry unequal costs, selection favors a biased threshold over an accurate one (Haselton and Buss, 2000). Judging whether another person’s attention is engaged with one’s own states is a judgment of that kind. The theory fixes no direction on its own, predicting over- or under-perception according to which error costs more. Which way the costs run for being understood is not established, and I assume an asymmetry rather than demonstrate one: missing a partner who does understand forfeits the relationship that would have followed, while crediting understanding that is absent costs an interval of misdirected effort. On that assumption a system that over-attributes is better designed than one that waits for proof, and the readiness of Section 3.1 is an individual setting of a threshold everyone carries.

An asymmetric threshold buys its advantage on credit, and interaction ordinarily settles the account. The cost of a false positive stays bounded because an inference gets stated, meets a correction, and the correction shows in what the partner does on later occasions. Where that return stops, an adaptive setting becomes a standing error that further experience with the same partner will not retire. What makes the configuration clinically significant is therefore not an unusually low threshold in the perceiver but an interaction that withholds recalibration.

### 2.3 Mentalisation and the theory-of-mind apparatus

Mentalisation, in the formulation of Fonagy and Target (1997) and Fonagy et al. (2002), denotes the capacity to interpret behavior in terms of underlying mental states; it is acquired in early caregiving relationships, selectively impaired by trauma, and varies within one person across emotional states, so that under relational stress even adults who mentalise well regress to lower-order modes of reading others.

Two patterns of impairment bear on the argument. Hypo-mentalisation is a failure to attribute mind where it is present. Hyper-mentalisation (Sharp et al., 2011; Sharp and Fonagy, 2015) is the attribution of mental states that go beyond the observable data, located primarily in borderline-spectrum adolescents, where it is associated with emerging borderline traits and declined over the course of inpatient treatment in one uncontrolled study (Sharp et al., 2013). Clinically it presents as reading motivation, intention and emotional depth into ambiguous behavior at high confidence and low evidentiary standards; related interpretive biases are documented in insecurely attached adult samples (Mikulincer and Shaver, 2007).

This apparatus supplies the perceiver-side machinery on which the IEE draws; the conditions under which its output goes untested lie elsewhere (Section 3.3, Table 1).

**TABLE 1:** Neighboring constructs, their locus and unit of analysis, and what the IEE would have to add to each.

Neighboring construct	Locus	Unit of analysis	What the IEE would have to add
Transference (Freud, 1912)	Perceiver	Biographical displacement	Present-interaction content
Hyper-mentalisation (Sharp et al., 2011; Sharp and Fonagy, 2015)	Perceiver	Individual difference	The maintaining condition, not the tendency
Idealization-devaluation (Kernberg, 1975)	Perceiver	Oscillating representation of the partner	Non-revision, where the cycle turns on inversion
Positive illusions (Murray et al., 1996a,b)	Perceiver	Dyadic perception (adaptive)	The failure-to-recalibrate boundary case
Perceived partner responsiveness (Reis et al., 2004; Crasta et al., 2021)	Perceiver	Continuous dyadic perception	Isolation of the decoupled sub-region; measure shared, no new operation
Projection of responsiveness (Lemay et al., 2007; Lemay and Clark, 2008)	Perceiver	Inference from one’s own responsiveness	The responder condition under which the projection is never corrected
Parasocial relationship (Horton and Wohl, 1956)	Perceiver	One-sided bond with a performer	A channel on which a correction can be delivered and is then re-asserted uncorrected
Barnum effect (Forer, 1949; Dickson and Kelly, 1985)	Perceiver	Acceptance of a generic description as self-descriptive	A standing relationship in which it is the recurring input
Projective identification (Klein, 1946; Bion, 1962)	Dyad	Unconscious evocation	The non-evocative variant
Traumatic bonding (Dutton and Painter, 1981; Effiong et al., 2022)	Dyad	Relational outcome	The upstream interpretive operation
Intermittent reinforcement (Skinner, 1953; Ferster and Skinner, 1957)	Dyad	Behavioral schedule	The content that is reinforced
Empathic accuracy (Ickes, 1993; Sened et al., 2017)	Dyad	Inferential performance on one occasion	Persistence across occasions, and the reversed direction of the discrepancy
Investment model (Rusbult, 1980; Rusbult et al., 1998)	Dyad	Commitment given satisfaction, alternatives, investment	The interpretive antecedent of investment; where alternatives are constrained, little
Narcissistic mirroring (Pincus and Lukowitsky, 2010)	Responder	Partner behavior	Perceiver-side interpretation, scored on contributions not on a diagnosis
Gaslighting and coercive control (Sweet, 2019; Stark and Hester, 2019)	Responder or dyad	Attack on the perceiver’s reality testing	Certainty from a vacuum rather than doubt from an attack; the two can overlap at the second route

### 2.4 Projection in romantic relationships and the conceptual gap

Psychoanalytic theory has the longest history with the phenomenon, from mirroring as a developmental need (Kohut, 1971) onward, and Table 1 sets out where three of its constructs sit: transference (Freud, 1912), projective identification, in which the content is evoked in the recipient rather than placed there (Klein, 1946; Bion, 1962), and the idealization-devaluation cycle (Kernberg, 1975). A second tradition documents traumatic bonding (Dutton and Painter, 1981, 1993; Casassa et al., 2022) and codependency (Carnes, 1997), in which attachment intensifies under intermittent abuse, with intermittent reinforcement as the behavioral account (Skinner, 1953). Effiong et al. (2022) report cross-sectional evidence that victims’ empathy was associated with traumatic bonding, which locates a perceiver-side variable in the pattern without establishing what it does there. The trauma literature supplies the clinical backdrop (Herman, 1992; van der Kolk, 2014).

Closest in territory is perceived partner responsiveness, the perception that a partner understands, validates, and cares for one (Reis and Shaver, 1988; Reis et al., 2004; Crasta et al., 2021). It measures felt understanding and makes no claim about what the responder demonstrates; Reis et al. (2017) distinguish feeling understood from being understood.

One line within that tradition bears directly on the claim made here. Lemay et al. (2007) and Lemay and Clark (2008) developed a projection account of perceived responsiveness, in which a perceiver’s own responsiveness toward the partner predicts what he perceives of the partner’s responsiveness, over and above the partner’s own report. Elevated attribution is therefore already accounted for, and an observed elevation tells against no projection model. What the IEE adds is the dyadic condition under which the projection goes uncorrected (Section 3.1). That condition is scored on the responder’s contributions, and the projection account leaves it open.

None of these literatures specifies a condition on the responder’s contributions under which an attributed understanding stops being tested. The claim made here is about maintenance: where the responder condition holds, early attribution constrains what later occasions are permitted to correct.

### 2.5 Measuring understanding: accuracy, bias, and the empathic accuracy paradigm

The account needs a measure of understanding that does not rest on the perceiver’s judgment. In the empathic accuracy paradigm (Ickes, 1993), partners are recorded in interaction, then each reviews the recording alone, reports the thoughts and feelings he had at marked points, and infers what the other reported at those points. Independent raters score each inference against the entry it targets (Ickes et al., 1990). The criterion is the target’s own report, collected separately and withheld from the person inferring. This is the in-vivo index the construct requires.

Four findings bear on the argument: accuracy is motivated and effortful, declining where an accurate reading would threaten the relationship (Simpson et al., 1995), with attachment organization governing how it is managed under those conditions (Simpson et al., 2011); its association with relationship satisfaction has been synthesized meta-analytically (Sened et al., 2017); and the procedure transfers to diary designs, so inference can be followed across the occasions on which relationships run (Howland and Rafaeli, 2010).

Correspondence alone does not settle the question, because a judgment can follow its object and still sit systematically above it. The truth and bias model separates tracking accuracy, the degree to which a judgment moves with the criterion, from directional bias, the tendency to sit above or below it, read at a stated criterion level (West and Kenny, 2011). That separation is the standard frame for perception in close relationships (Fletcher and Kerr, 2010; Overall et al., 2012), and the IEE makes a claim about both at once.

The criterion, the target’s own report, is obtained independently of the inference and is not thereby infallible; it is serviceable with its error documented (Supplementary Material 1, Section 9). And direction matters: how accurately A reads B, and how well founded B’s belief that A understands him, are separate questions, and the IEE turns on the second, so the version used here scores the responder’s inferences about the perceiver against the perceiver’s own report while felt understanding is rated by the perceiver, the pairing on which Section 3.5 and H5 rest.

A responder with an interest in appearing understanding can shape what he puts on record, which raises his scored demonstrated understanding and shrinks the predicted surplus, so the bias runs against the hypothesis.

## 3 Theoretical extension: the interpersonal ELIZA effect

### 3.1 Definition

The Interpersonal ELIZA Effect (IEE) denotes a dyadic configuration in which a perceiver’s readiness to be understood meets a responder whose contributions either set determinate attributions about him at a rate below a coded threshold, or set such an attribution again, uncorrected, after he has put it right, so that little in the interaction calibrates what the perceiver credits him with.

Two quantities carry the account, each measurable without the other. Felt understanding is the perceiver’s appraisal that the partner grasps his inner states, history, and concerns, assessed at the level of the relationship (Reis et al., 2004; Crasta et al., 2021) or occasion by occasion in interaction-based designs. Demonstrated understanding is what the responder puts on record about the perceiver’s states in a form that can be checked against the perceiver’s own account of them. It is not a measure of what he reveals about himself. Two quantities are kept apart under that heading: the attributions he sets about the perceiver within the interaction, from which the responder condition is coded, and his inferential accuracy about the perceiver’s states in the probe of Section 3.5, against which the surplus is computed.

**The responder condition.** The responder condition is met where, and only where, one of two things holds of the responder’s attributions about the perceiver: either the responder produces determinate content elsewhere on matters as intimate as the perceiver’s own reports while the assessable occasions on which his contributions set a determinate attribution about the perceiver fall below a rate provisionally fixed in Supplementary Material 1, or an attribution that has been explicitly and literally disconfirmed is re-asserted in its uncorrected form on later, independent occasions. There is one criterion, calibration by what the responder puts on record, and two ways of failing it. A single lapse does not meet the second route: Supplementary Material 1 provisionally requires two documented re-assertions. The condition is the claim, and the manual is one provisional way of scoring it (Section 6.3). The two routes need not select the same responders, and how far they overlap is an empirical question the coding study reports; H2 assigns them as separate conditions.

**Specificity is a threshold; accuracy is graded.** Specificity is the degree to which an attribution about the perceiver is determinate enough to be wrong. “You are someone who needs honesty” is true of nearly anyone, and no perceiver could truthfully reject it. “You went quiet because what was said about your brother landed harder than you let on” can be confirmed or rejected. It enters the criterion at that threshold only and is graded above it. Accuracy is the correspondence of such attributions to the perceiver’s own account of his states, scored by the empathic accuracy procedure (Ickes, 1993).

**Persistence rather than echo.** A responder who absorbs a correction in his next turn and carries no corrected attribution afterward has produced an echo, which is what ELIZA produced (Section 2.1).

**Corrections must have been available.** A re-assertion is scored only where the correction was available to the responder: explicit and literal, delivered in a register in which he demonstrably reads other content, and followed by an occasion on which the corrected content was in play. Where any of these is absent the occasion is not scored, so the criterion reaches only responders for whom a correction was available, and no judgment about motives enters the coding.

**Occasions are created rather than awaited.** Where a trigger in the coding manual is met, the perceiver states a determinate content about his own state as it arises, and the following occasions are scored for whether any contribution sets a determinate attribution about him. Neither acknowledgment nor disagreement is scored as a failed attribution, and whether the responder acted on what he read enters no score. A dyad without assessable contributions is not classified, and a component that cannot be judged never enters as a low value: failure to show understanding is not shown failure to understand.

**The test case.** A skilled person-centered practitioner discloses almost nothing about himself and satisfies any criterion built on self-revelation. Rogers (1957) requires that empathic understanding be experienced and communicated, a condition on what gets across that licenses no verbal form. Competent practice states the inference, offers it for correction and carries the correction forward, so the practitioner stands outside the responder condition by the criterion and not by exemption (Supplementary Material 1, Section 12).

**Two situations lie close to it.** General reserve, where determinate content is sparse throughout, does not establish the first route, which requires such content elsewhere and its absence about the perceiver only; where neither route can be established the dyad is not classified. ELIZA is such a responder: the script set no attribution about the user at all, so the dyads Weizenbaum described would be not classified under this criterion, like the near-silent partner of the second composite case (Supplementary Material 1, Section 15). The name is kept for what it has named since Hofstadter (1995), understanding read into a record that does not warrant it, and not for the program’s own contribution; the criterion is narrower than its namesake by design, since a rule reaching every sparse responder would classify the reserved partner with him (Section 6.3).

**Indicators.** Self-disclosure enters as a covariate of discoverability, how much material about the responder is available to be read at all, and not as a criterion. Three recurrent forms set no determinate attribution about the perceiver: non-attributive return of his own words, affiliative endorsement of a position he supplied, and indeterminate self-statement no observation could contradict. Repetition is scored by what it sets, since wording he supplied can still carry an attribution that can be wrong.

**What is decided and what is graded.** The two conditions the account joins are not of the same kind, and the difference governs everything downstream. The responder condition is decided: it is coded from recorded interaction by the manual of Supplementary Material 1, whose rules spread the assessable occasions of the first route across at least three encounters and require of the second repeated re-assertions on later, independent occasions under separation rules that differ by kind of material (its Sections 6.4 and 6.5); and a dyad meets it, fails to meet it, or is not classified. Perceiver readiness is graded, measured as set out in Section 6.4, and never cut into groups, with no threshold proposed or to be read into it. The configuration is therefore not a category a dyad falls into. It is the responder condition met, taken together with a level of readiness, and what the account predicts is that the surplus grows with that level. That surplus, a standing excess of felt over demonstrated understanding which does not decline as disconfirming episodes accumulate, is stated as a prediction in Section 3.4 and is no part of the definition. Where that surplus grows with readiness no more in dyads meeting the condition than in others, and the probe has met the evidential standard of Section 3.4, the account is wrong.

**Vocabulary.** No term introduced here names a kind of person; the responder condition is a property of contributions within an interaction. The name of the construct is not exempt from that rule: no one is an ELIZA, and the word marks a configuration and never a partner. The two routes mark the difference between a vacuum and a contradiction, and Section 3.3 separates the configuration from gaslighting on that line.

### 3.2 Mechanisms within the configuration

Both sides of the configuration are active: two classes of perceiver-side mechanism build felt understanding and then hold it against material that would correct it, while the responder’s uptake of a correction is performed or omitted on each occasion, so it has a rate and a course rather than a fixed value.

Which quantity each operation moves. The target quantity is felt understanding (Section 3.1). Attributed depth, the interiority credited to the partner, is a second quantity, and the two come apart: a partner can be credited with an inner life nobody reaches and with registering almost nothing of what the perceiver needs. Two of the five sub-operations work on depth and are marked accordingly; the bridge to it, that concealed interiority implies the resources to grasp, is a conjecture rather than a step that follows.

#### 3.2.1 Inflated meaning attribution

Three sub-operations differ in what the perceiver inflates.

##### 3.2.1.1 Non-attributive mirroring read as understanding (felt understanding)

The operation covers the return of the perceiver’s vocabulary and values in the non-attributive form of Section 3.1, which requires no inference about him and sets no determinate attribution; repetition that carries a correctable inference about him is scored by that content and falls outside this sub-operation. The return of his own material is read as evidence that his states have been grasped.

##### 3.2.1.2 Completion of minimal cues (attributed depth)

Where a contribution is indeterminate, the perceiver completes it from his own emotional repertoire and experiences the resulting depth as the responder’s. What rises directly is depth, and felt understanding follows only across the bridge.

##### 3.2.1.3 Attribution to ambiguous or absent feedback (felt understanding)

The perceiver infers where the responder supplies little or nothing, settling on the most generous reading, under which a silence or a brief message becomes evidence that his own state was registered.

#### 3.2.2 Protective re-attribution

The second class holds what has been attributed against contradicting material, and receives only a subset of it: communication is processed under a standing presumption of honesty abandoned only when a trigger forces reassessment (Levine, 2014). A contradicted reading is such a trigger, and the claim about the second route is that it is commonly absorbed rather than registered: a repetition after correction is read as poor expression rather than poor understanding. Where absorption fails the perceiver revises, and P2 predicts that outcome becomes rarer.

##### 3.2.2.1 Intermittent warmth renewing the attribution (attributed depth)

When attentive episodes alternate unpredictably with withdrawal, they gain salience, the operant pattern Skinner (1953) described with warmth in place of a discrete reinforcer. What is strengthened is an interpretation with its affective state: the warm episode shows the depth was there throughout. Only across that bridge does it restore what the perceiver credits the partner with having grasped, and the two are scored separately (Section 3.4).

##### 3.2.2.2 Retrospective re-coding of devaluation (felt understanding)

Over longer horizons, episodes that in isolation would be demeaning are re-coded as complexity, dark humor, or an unconventional way of caring, each reading leaving intact the claim that the partner knows what the perceiver is. Cost drives the re-coding: at face value the episode obliges him to revise the attribution and to act on the revision. Expectations formed early supply the template (Berne, 1964, 1972).

#### 3.2.3 The maintenance loop

Four paths are proposed as the core of a loop, and a fifth intensifies it without being required. Each has separate support, none has been observed in this sequence within a dyad meeting the responder condition, and Figure 1 marks R3 as the optional amplifier.

**FIGURE 1:**
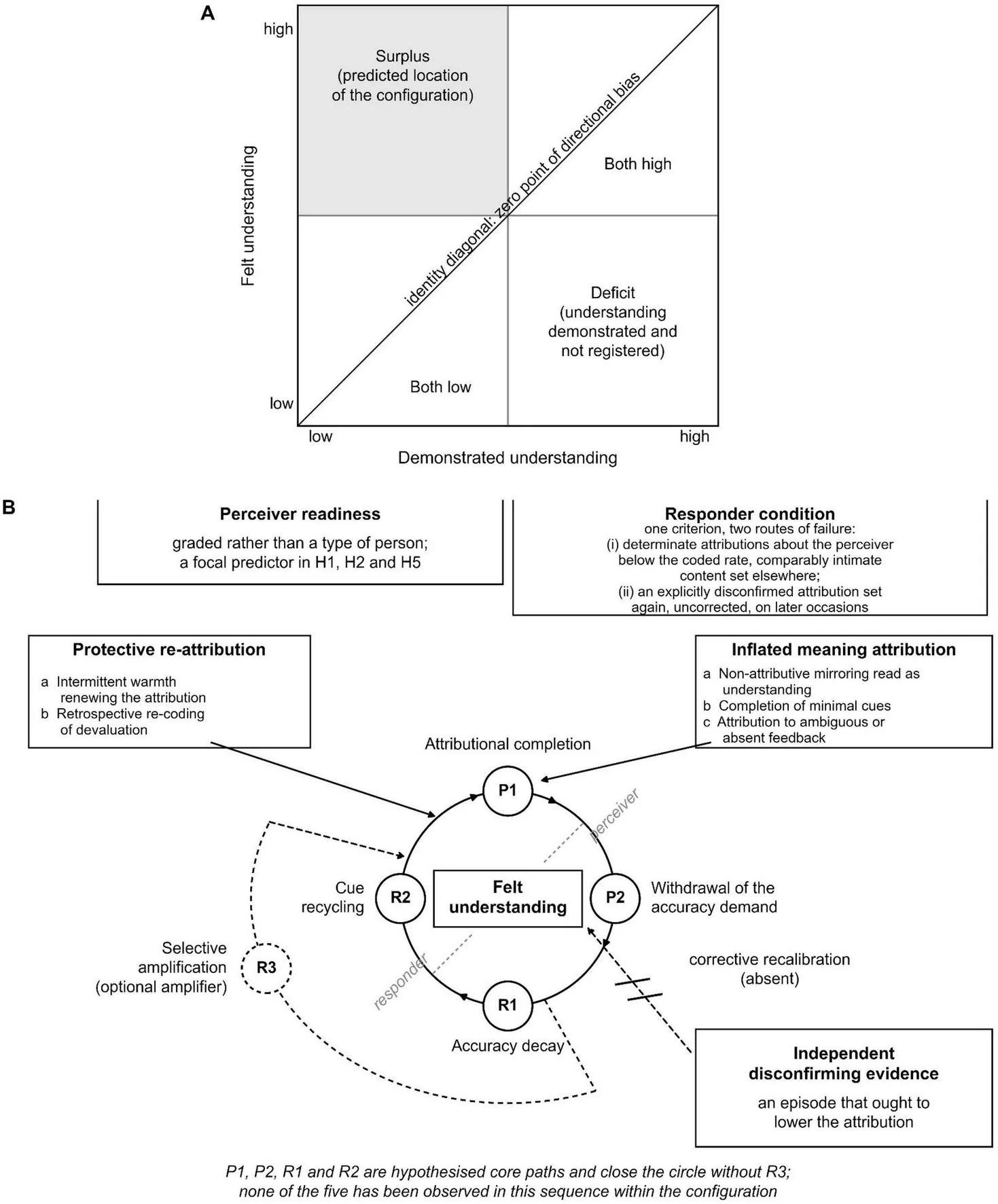
The Interpersonal ELIZA Effect. **(A)** Felt and demonstrated understanding on the shared correspondence metric. The diagonal marks equality of the two and is the zero point of directional bias. The upper right and lower left lie on both sides of it, so in those two the direction of bias is read from position relative to the diagonal and not from the quadrant. Of the four regions, the upper left is the predicted location of the configuration and not its definitional one, since the surplus is stated as a prediction in Section 3.4 rather than built into the definition. Section 5.4 treats the states lying near the configuration without meeting it. **(B)** The maintenance loop of Section 3.2.3. The two boxes at the top are the two conditions the account joins: the responder condition, which is coded and decided, and perceiver readiness, which is graded and never cut into groups. The loop maintains the configuration and does not define it. P1, P2, R1 and R2 are the hypothesized core paths, drawn solid, and close it without R3, dashed outside it as an optional amplifier. The struck-through arrow is the corrective recalibration held to be absent.

**P1. Attributional completion.** The perceiver supplies the inferential content the responder’s contribution leaves open.

**P2. Withdrawal of the accuracy demand.** A perceiver who supplies the missing understanding stops asking for it, and the rate of repair initiations, clarification requests and disconfirmation probes is recorded from the first occasion. Classification follows Section 3.1 and is not revised by the course a dyad takes: where that rate holds steady while felt understanding rises, P2 has failed and the dyad still meets the responder condition.

**R1. Accuracy decay.** Empathic accuracy is motivated and effortful rather than fixed, varying with what an interaction makes consequential (Simpson et al., 1995, 2011). My extension: where accuracy about the perceiver is neither requested nor consequential, it is not recruited and demonstrated understanding stalls.

**R2. Cue recycling.** The responder’s contributions come increasingly to consist of material the perceiver has supplied, scored as the proportion traceable to prior input. The MEMORY stack of Weizenbaum’s (1966) script did exactly this, returning the loop to P1 with less to constrain the inference.

**R3. Selective amplification.** Responders calibrate to what the perceiver rewards, so the distribution of warm episodes is partly an output of the loop. R3 intensifies the configuration without being required: with it removed, P1, P2, R1 and R2 still close. Self-fulfilling idealization is established in functional dyads (Murray et al., 1996b), and its operation under the responder condition is predicted, not shown.

**Path dependence is a further conjecture.** Mutual influence does not secure it: two quantities that act on each other can reach a common equilibrium from unlike starting points. That dyads facing identical later conditions differ by the early order in which accuracy was demanded and supplied, and that a configuration outlasts its producing conditions, are separate predictions entailed by none of the paths above.

**Two readings, and what separates them.** Both are bivariate autoregressive models in which felt understanding is predicted from perceiver readiness, demonstrated understanding at the same wave, their product and its own previous value; Model A stops there, and Model B adds two cross-partner paths, from felt understanding at t-1 to demonstrated understanding at t, where R1 and R2 would show, predicted negative, and from demonstrated understanding at t-1 to felt understanding at t, where P1 and P2 would show, predicted positive and smaller where the responder condition is met. A test on two degrees of freedom rejects Model A when either path alone contributes, so each path is reported with its own interval and the loop claim requires both; the field module of H5 carries it (Supplementary Material 1, Section 14), and where Model B fits better a dynamic account is needed, with no causal reading and no claim of uniqueness.

### 3.3 Differentiation from neighboring constructs

Table 1 sets each neighbor against the configuration on locus, unit of analysis, and what the present account would have to supply that the neighbor does not. Where that answer is thin, the cell says so, and four differentiations need more than a cell.

**Parasocial relationship.** Horton and Wohl (1956) described intimacy with a performer who composes his content for an audience and never receives what a viewer reports about himself. It is a closer structural neighbor than transference and the easiest to mistake for the configuration, since non-reciprocity holds in both; the separating line is the availability of a correction. A viewer supplies no scorable occasion, because nothing he says about his own states reaches the performer, and such bonds are not classified here. A companion system receives those statements and returns text conditioned on them, so a correction is available in the sense fixed in Section 3.1, and the second route reaches it as it reaches a human responder (Section 6.2).

**Investment model.** Rusbult’s model derives commitment from satisfaction, quality of alternatives and size of investment (Rusbult, 1980; Rusbult et al., 1998), with no appeal to attribution. Where alternatives are constrained by material dependency or by children, it accounts for persistence more economically, and the configuration claims nothing there. The two predict on different outcomes, commitment against surplus, and both can hold at once, so I mark this as a demarcation and not a test; only a common outcome such as persistence would put them at odds, and none is specified here.

**Gaslighting and coercive control.** The two routes separate a vacuum from a contradiction. A responder who meets the first disputes nothing: he produces determinate content elsewhere on comparably intimate matters, leaves what the perceiver says about himself unanswered, and the perceiver fills that space with unearned certainty. Gaslighting runs the other way, working on that account until the perceiver doubts it (Sweet, 2019; Spear, 2020), and coercive control organizes it into domination (Stark and Hester, 2019). The second route is where the two meet: a responder who re-asserts an attribution after explicit disconfirmation contradicts the perceiver, and such a re-assertion can occur within either. Meeting the second route establishes neither, since both rest on a pattern of domination or reality-undermining that the criterion does not assess.

**Shared measures.** Felt understanding is assessed with instruments from the responsiveness tradition (Reis et al., 2004; Crasta et al., 2021) and *in vivo* with the empathic accuracy procedure (Ickes, 1993). I claim no discriminant validity against either, since a construct cannot discriminate against its own measure: the claim is a sub-region of that distribution, read in the opposite direction.

### 3.4 Hypotheses

The predictions follow from the responder condition of Section 3.1 and from readiness as a graded quantity, and each falsification condition carries an equivalence bound fixed in advance (Lakens, 2017), stated in points of the three-point metric of Section 3.5. For a contrast of directional bias the bound is 0.3 points; for a quantity that scales with a continuous predictor it is 0.1 points per standard deviation of that predictor, a third of the first; H3 is stated on attributed depth and carries 0.2 standard deviations of the depth change score; proportions carry a bound in percentage points. The comparability criterion of the probe carries a bound of its own, 0.15 points, half the contrast bound, since a bound on measurement equivalence has to lie inside the smallest difference the hypotheses treat as substantive on that metric (Supplementary Material 1, Sections 9.5 and 14.1). All are planning values I propose and none is a threshold whose clinical relevance has been established. The neighboring values and the design detail of each hypothesis are in Supplementary Material 1, Section 14.

Felt understanding is the outcome in every prediction but H3; attributed depth, the second quantity of Section 3.2, is secondary where a mechanism predicts it.

**H1 (Perceiver readiness).** *Design*: the probe of Section 3.5 in established relationships, over a coding block that must reach the minima of Supplementary Material 1 before the condition is coded. *Independent variables*: the two readiness facets of Supplementary Material 2, entered separately and never as a composite, with attachment anxiety (Brennan et al., 1998) alongside; the responder condition is coded from the same material and entered with readiness and their product. *Dependent variable*: directional bias at the common level c of Section 3.5, with the tracking slope estimated alongside (West and Kenny, 2011). *Estimand*: the difference between the change in directional bias at c per standard deviation of the threshold facet in dyads meeting the responder condition and the same change in dyads not meeting it, each a model-implied expectation in points under Section 3.5, adjusted for the motivation facet and for attachment anxiety. *Prediction*: that product term is positive, since Section 2.2 locates the failure in the interaction and not in a threshold the perceiver carries; the main effect of the threshold facet is secondary and reported beside it, and alone would be consistent with a perceiver-side account this article does not claim to improve on. *Falsification*: that difference inside the per-standard-deviation bound, which is the case Section 3.1 names as the account being wrong, on condition (b) stated after Table 2. Unclassified dyads and codability are treated in Supplementary Material 1, Section 14.

**TABLE 2:** Design, estimand, bound, reach and withdrawal consequence of each prediction.

Prediction	Design	Estimand	Bound	What the test reaches	Failure withdraws the construct?
H1, perceiver readiness	probe in established relationships, condition coded over a block that meets the manual’s minima	difference between met and not-met dyads in the change in directional bias at c per SD of the threshold facet	0.1 points per SD	the coded condition as moderator of a perceiver-side slope, no causal claim	yes, on condition (b)
H2, responder condition	single-session analog, three conditions within person, blocks matched on obtained demonstrated understanding	directional bias at matched demonstrated understanding in re-assertion and low-rate blocks against carried-correction blocks, and its widening with readiness; as an additional prediction, the marginal level of felt understanding in those blocks (0.3 points)	0.3 points; 0.1 points per SD	assigned counterparts of the two routes, not the coded condition	no
H3, unpredictable warmth	vignette experiment, cyclic against random order of one multiset	difference between arms in the change in attributed depth from first to last rating	0.2 SD of the change score	a perceiver-side mechanism (Section 3.2.2.1), neither route	no
H4, failure to recalibrate	two blocks, feedback and no-feedback arms within each level of the condition	difference between the levels, within the feedback arm, in the change in directional bias at c from before to after the release	0.3 points	resistance to a correction arriving from outside the dyad	no
H5 (i), level	analog arm of H2	directional bias at matched demonstrated understanding, counterpart against comparison blocks, and its rise with readiness	0.3 points, and 0.1 points per SD	the assigned counterpart, absorbed where the projection and accuracy terms account for it	no
H5 (ii), tracking	as (i)	difference in the slope of felt on demonstrated understanding	0.1 points per point	as (i)	no
H5 (iii), updating	as (i), after a disconfirmation running toward the perceiver	within-person change in the felt component, counterpart against comparison blocks, over the projection and accuracy terms	0.3 points	the discriminant claim against projection, on the assigned counterpart	yes, on conditions (a) and (b)
H5 (iv), maintenance	field module, six waves, condition coded cumulatively, read by route	change in directional bias at cover a protocol-fixed horizon from entry wave in met dyads, and its difference from the same change in not-met dyads, both as non-inferiority	minus 0.3 points on each	the coded condition in standing relationships, per route, no causal claim	yes, on a route adequately tested, on condition (b)

Every estimand but H3’s is an expectation implied by the fitted model in points of the three-point metric of Section 3.5, H3’s on the six-point depth format of Supplementary Material 2, Section 6, each read with a 90 per cent interval as stated above; “per SD” is per standard deviation of the predictor named; the letters in the last column refer to the conditions stated below the table.

**H2 (Responder condition).** *Design*: a single-session analog with a scripted or model-driven responder and three conditions assigned within person across blocks, with output volume, responder self-disclosure and the third-party content that passes the selectivity gate held constant: determinate attributions set and corrections carried; set and re-asserted uncorrected; and set at a rate below the threshold. Specificity and persistence are not crossed, because they cannot be (Supplementary Material 1, Section 14). *Dependent variables*: felt and scored demonstrated understanding, block by block, on the shared metric, with blocks matched after the fact on the obtained level of demonstrated understanding. *Primary prediction*: at matched demonstrated understanding, directional bias is larger in the re-assertion blocks and in the low-rate blocks than where corrections are carried, on the contrast bound, and each gap widens with readiness, on the per-standard-deviation bound. *Additional prediction*: the marginal level of felt understanding is higher in those blocks, the stronger claim. *Falsification*: either difference inside the bound, or larger bias where corrections are carried.

**H3 (Maintenance under unpredictable warmth).** *Design*: a vignette experiment, daily diary secondary. *Independent variable*: the assigned order of warm and cold episodes, cyclic against random, one multiset serving both arms so that mean and dispersion are identical by construction and only the order differs; the limits on the random arm, the construction control on the two orders and the manipulation check are in Supplementary Material 1, Section 14. *Dependent variable*: attributed depth credited to the responder, on the four-item adaptation of Supplementary Material 2, Section 6, rated after each episode and analyzed as the change from first to last rating, which is what the mechanism of Section 3.2.2.1 acts on, with felt understanding alongside; scripted attributions hold demonstrated understanding constant. *Prediction*: lower predictability, greater increase in attributed depth. *What this hypothesis does not do*: the step from attributed depth to felt understanding is a conjecture (Section 3.2.1.2). *Falsification*: a difference between arms in the change score inside 0.2 of its standard deviations; arms the manipulation check cannot separate leave the hypothesis untested.

**H4 (Failure to recalibrate).** *Design*: two blocks, the first supplying the material the responder condition is coded from, which must meet the minima of Supplementary Material 1 since a single baseline session cannot code it, and the second carrying a probe in each of at least four episodes, with dyads assigned to a feedback arm and a no-feedback arm within each level of the condition; the single release and the strata either side of it are fixed in Supplementary Material 1, Section 14. *Dependent variable*: change in directional bias at fixed demonstrated understanding, with both components reported separately, since a narrowing on the demonstrated component records a responder who has learned. *Estimand*: within the feedback arm, the difference between the two levels of the condition in the change in directional bias at c from the episode before the release to the last episode after it, each a model-implied expectation in points. *Prediction*: comparison dyads move further toward the scored record than dyads meeting the responder condition; neither group is selected on its baseline discrepancy, which is entered as a covariate for both, and the no-feedback arm measures the regression directly. Feedback-related gains in empathic accuracy (Marangoni et al., 1995) motivate the design without establishing the predicted improvement. *Falsification*: equivalent movement across the two levels, inside the contrast bound.

**H5 (Discriminant prediction).** Design: the analog arm of H2, where the responder’s uptake of corrections is assigned, with a field module alongside it whose waves, lag, sampling and per-path bounds are specified in Supplementary Material 1, Section 14; readiness is entered continuously and never cut into groups. Assignment removes the range restriction that selection on a predictor would produce (Sackett and Yang, 2000); the field module’s handling of it is in Supplementary Material 1, Section 14. Measures: felt and scored demonstrated understanding on the shared metric, and the perceiver’s own rated responsiveness; felt understanding before the disconfirmation is an outcome in its own right, since (i) says it differs by condition, and (iii) is tested without adjusting for it (Supplementary Material 1, Section 14). Predictions: (i) at matched obtained demonstrated understanding, which is measured and not assigned, directional bias is higher in the blocks assigned the condition’s counterpart, on the contrast bound, and rises with readiness, on the per-standard-deviation bound; (ii) the tracking slope is lower by more than 0.1 points per point of demonstrated understanding; (iii) after the responder has stated an attribution about the perceiver at odds with what the perceiver reported, evidence running toward the perceiver, felt understanding falls in comparison blocks and holds in those assigned the counterpart; (iv) in the field module, directional bias at c does not decline across the waves that follow a dyad’s meeting the condition, and declines there no further than in dyads not meeting it. The estimate takes every dyad from its entry wave, the first at which it met the route in question within the module, over a horizon in waves since entry fixed in the protocol and shared with the comparison dyads, with a change of classification on either side recorded rather than excluded, since path P2 makes corrections rarer where the loop operates and a test conditioned on them would select on what the mechanism moves; the estimate on waves with an assessable correction is a sensitivity analysis, and (iv) is decided by route (Section 6.3; Supplementary Material 1, Section 14.13). The rival: projection of one’s own responsiveness (Lemay et al., 2007) yields elevation and loose tracking too and already carries an accuracy path, so (i) and (ii) settle nothing alone. What (iii) asks for is an increment on updating after disconfirmation over the projection and accuracy terms. Falsification: if the felt component falls as far in blocks assigned the condition’s counterpart as in comparison blocks, or if its effect on updating lies inside the bound with those terms entered, the discriminant claim fails. If (i), or (i) and (ii), is absorbed, its contrast falsified once the projection and accuracy terms are entered (Supplementary Material 1, Section 14.9a), the level claim is unsupported and the account stands on (iii). For (iv), each of its two estimands, the change within the dyads meeting the condition and the difference between that change and the same change in the comparison dyads, is a prediction of non-inferiority, supported where the lower limit lies above minus 0.3 points and failing where the interval lies below it; on a route, (iv) is supported only where both are supported and fails where either fails (Supplementary Material 1, Section 14). The construct is withdrawn where (iii) or (iv) is falsified, whether or not the other prediction is undecided, on the conditions stated after Table 2. The floor condition on reading (iii) is in Supplementary Material 1, Section 9.

**How each result is read.** Every reported quantity carries a 90 per cent interval, read against its bound and against zero, with one of four readings recorded: supported, where the interval lies entirely beyond the bound in the predicted direction; falsified, where it lies entirely inside the bound; opposite, where it lies entirely beyond the bound the other way, which also falsifies and is reported apart from a null; and undecided, where it covers the bound, in which case the prediction is reported as untested on that sample. H5 (iv), which predicts an absence of decline, is read the other way round. Table 2 states for each prediction its design, estimand, bound, what the test reaches and whether its failure withdraws the construct. Three failures withdraw it, H1 and H5 (iii) and (iv), on the conditions stated after the table; the other predictions bear on mechanisms and on what the configuration adds, and their failure narrows the account without withdrawing it.

**The conditions on withdrawal.** (a) For (iii), the manipulation met the implementation standard fixed in the protocol before collection (Supplementary Material 1, Section 9.8c). (b) For H1 and for (iii) and (iv), the probe had met its evidential standard, evidence supporting its intended use under Supplementary Material 1, Section 9.5. That standard is fixed in the protocol before collection, names its quantities, the value each must reach and who judges it, and is assessed before the confirmatory results for H1 and H5 are inspected; the comparability criterion against the modified video-cued procedure described there is a necessary part of it and not, on its own, sufficient; for H1 the readiness measure must also have passed Stage 1 of Supplementary Material 2, Section 10. Where a condition is unmet, the result, whichever way it runs, stands as a finding about the procedure run, and the mechanism is reported as not adequately tested, neither supported nor withdrawn.

### 3.5 Analytic strategy

**The demonstration probe.** As an episode occurs, the perceiver records a determinate content of his own, and the responder records, without access to it, what he took the perceiver to be conveying. The perceiver then rates each of his own contents, on the scale the responder’s record is scored on, for how far he takes the responder to have grasped it, which is what puts the two quantities on one metric. Blind raters score the responder’s record against the perceiver’s own (Ickes, 1993), adapted in that the content is stated as the episode runs rather than reconstructed from it. Corrections run in both directions: one addressed to the responder is stated to him in the interaction, elicited at the end of each observation period where none was spontaneous (Supplementary Material 1, Section 8.1), and its uptake scored on later independent occasions, while the scored record of what he missed goes back to the perceiver on designated occasions only. The full procedure and the treatment of releases are in Supplementary Material 1, Sections 9 and 14.

**The common metric.** Both are scored over the same contents on one three-point correspondence scale. Mean-level discrepancy is the average difference between felt and demonstrated understanding. Tracking, the within-dyad slope of felt on demonstrated understanding, is estimated separately (West and Kenny, 2011). Conditional discrepancy is the model-estimated difference at a stated criterion level. That level, c, is fixed before analysis at 1.0, the midpoint of the three-point metric, because H5 (ii) predicts that the slopes differ and a conditional difference taken where slopes differ depends on where it is taken and can change sign. Every other term is held at a value fixed in Supplementary Material 1, Section 14; a conditional comparison is a difference taken at those values, reported again at two neighboring levels of c, and one whose sign changes carries no directional claim.

**Both components.** Felt and demonstrated understanding are modeled as separate outcomes, since the discrepancy also narrows when demonstrated understanding rises, asking nothing of the perceiver. The perceiver’s own responsiveness is measured at baseline by self-report, on Lemay et al.’s (2007) own-responsiveness items, adapted in the analog, whose projection account predicts the same elevation. No reverse probe is run, so the term is a self-report covariate and not a scored quantity on the metric above.

**Multilevel structure.** Contents nest within episodes within dyads, and all three levels are carried; the middle level is the episode of Supplementary Material 1, Section 9, and not the occasion of its Section 3, since the perceiver’s probe contents are written and open none. Demonstrated understanding is centered at c and entered as its dyad, episode and content parts separately, because a slope fitted on the undecomposed predictor mixes all three and only the last is the quantity the tracking claim is about; the specification, with the random effects and their anchoring, is in Supplementary Material 1, Section 14. The metric is ordinal; the reported quantities are not. The three-point scale has ordered categories and no established spacing, so the primary models are cumulative-link mixed models, with linear models alongside as a sensitivity analysis. What those models report is not their coefficients but the expectations they imply on the observed metric: expected felt understanding at a stated level of demonstrated understanding, with covariates at stated values; directional bias at c is that expectation minus c, the tracking slope is its change per point of demonstrated understanding, and a contrast is the difference between two such expectations, all in points, with intervals by the delta method, by simulation from the fitted model or, where sets share dyads, by resampling dyads (Supplementary Material 1, Section 14.13). This is what keeps the bounds of Section 3.4 testable, since an interval on a latent logit scale cannot be compared with a bound stated in points; descriptive means treat the metric as though its steps were even, which they are not, and are reported as descriptions only. Those bounds are fixed in advance (Lakens, 2017); reading them on model-implied expectations rather than on coefficients is an extension of that procedure which I propose here and which has not been validated elsewhere.

## 4 Vulnerability factors and clinical manifestations

Perceiver readiness is one of the two conditions of Section 3.1, graded rather than present or absent. Three developmental routes raise it and hold it high, attachment insecurity (Section 4.1), complex post-traumatic stress (Section 4.2) and codependent scripts (Section 4.3); they differ in the evidence behind them and each is marked accordingly. Three composite cases in Supplementary Material 1, Section 15, one for each developmental route, illustrate them without being evidence for them; the second, a near-silent partner, is left unclassified and is the case the rule stands to misclassify (Section 6.3).

### 4.1 Insecure attachment styles

Adult attachment classifications (Bartholomew and Horowitz, 1991; Mikulincer and Shaver, 2007) distinguish secure, anxious-preoccupied, dismissive-avoidant and fearful-avoidant styles, on the theory Bowlby (1969) founded, measured dimensionally in the form H1 uses (Brennan et al., 1998). Readiness runs high in the anxious-preoccupied and fearful-avoidant ranges.

The anxious-preoccupied pattern carries a working model in which affection is precarious and the partner’s availability intermittent, with close monitoring for approach and withdrawal. That is readiness in the sense of Section 3.1: high motivation to be understood, with a low threshold for treating an ambiguous contribution as evidence of it.

The fearful-avoidant pattern combines that surveillance with suppression, and it bears on the coding rule. Such a perceiver withholds the determinate statement about his own state that would give the following occasions something to be scored against, so few occasions arise on which a correction could have been carried. The elicitation procedure of Section 3.1 exists for this case.

Secure classification is no exemption: where loss or isolation raises readiness, the configuration can develop in a securely attached perceiver. In my clinical experience such perceivers test an interpretation early, take a disconfirming answer at face value, and leave a dyad that does not change. The nearest work reports biased perception under insecurity (Overall et al., 2015) and licenses only the inverse inference. Section 3.2.3 gives it a testable form: secure classification should predict an accuracy-demand rate, in the sense of Supplementary Material 1, Section 9.9, that does not fall while felt understanding rises.

These readings mark the boundary against adaptive idealization. Positive illusions raise a partner’s standing on the traits the perceiver values (Murray et al., 1996a,b); what rises here is unflattering as often as not, as when the silence after a quarrel is read as shame he cannot speak of, which lowers his standing and credits him at once with having registered what the perceiver could not say, and it is felt understanding that the predictions turn on.

### 4.2 Complex post-traumatic stress and the threshold for attribution

Complex post-traumatic stress follows prolonged interpersonal trauma and alters affect regulation, self-concept and the relation to others (Herman, 1992; van der Kolk, 2014). What follows comes from clinical work, untested in the form I state it in and stated so that a study could find it absent.

The trauma-trained perceiver does not take in more than others; he settles sooner. A brief silence or a shift in vocal tone suffices to fix a determinate attribution about what the partner feels and why, at a confidence others keep for an explicit statement. The threshold is lowered, and the cues that move it are those that once carried danger.

A child whose caregiver alternates between warmth and danger has reason to decide early on minimal information, because deciding late is expensive. The nearest empirical work concerns that child: physically abused children identified facial anger correctly from less sensory input than controls, facilitated access rather than lost accuracy (Pollak and Sinha, 2002), and those children were right. What carries over is the lowered threshold and no claim about the judgment it yields; the step to an attribution of understanding between adults is an analogy I have not tested.

Readiness rises as the counterpart of chronic invalidation: being understood accurately is what such a perceiver most wants and least expects, and a contribution that sets no determinate attribution is the material a lowered threshold works on.

The therapeutic difficulty is that the operation was once protective: an instruction to read less into things asks the perceiver to surrender the skill that kept him safe (Section 5.2).

### 4.3 Codependent relational scripts

Codependency is a clinical heuristic rather than a validated construct, and I use it descriptively, as Carnes (1997) does: a pattern in which self-worth is drawn from the partner’s emotional functioning, organized by the proposition “I matter when I am needed.”

What such a script adds is specific: it converts the responder’s opacity into an opportunity. A partner who is unsettled gives the perceiver a task, and the depth credited to him is an over-attribution and a use at once, since it makes the perceiver necessary. The attribution thus draws support from outside the partner, and the loop of Section 3.2.3 runs on it.

In my clinical experience this profile is the hardest of the three to interrupt, because interruption puts the perceiver’s account of himself at stake with the relationship; that ordering is untested. Work here runs beside work on the autonomous sources of self-worth (Section 5.2).

## 5 Formulation, safeguards and questions for practice

The configuration has two uses here: naming it organizes presentations that otherwise resist formulation, and its components are separable enough that each names a target for research whose value is untested.

### 5.1 Diagnostic considerations

Recognition is a formulation task that can sit alongside established diagnoses, and no diagnostic category is proposed.

Three signals in the perceiver’s presentation raise the question: a conviction of being understood that outruns what the partner has put on record, arriving in report as attributed depth rather than the quantity scored; the sense that the relationship holds something profound the perceiver cannot demonstrate; and repeated hurt, each time re-coded as misunderstanding rather than read as information about the partner. Two further signals follow the two routes: a partner who speaks determinately about other people’s inner lives at the intimacy the perceiver has offered about his own and says little of the kind about him; and something the perceiver said plainly about his own state, datable, which the partner has since contradicted more than once. All are reports about a course of events, not findings about the partner.

The partner’s reasons enter the formulation and not the criterion. Where either route is met, they would bear on what is to be done, since safety and viability arise with one kind of responder and not another.

### 5.2 Intervention targets as questions for research

The structure of the configuration marks three targets, each served by an established tradition, and none is recommended here as a treatment: no trial has tested any of them against the configuration, and the loop they would interrupt is a model and not an observed sequence (Section 6.3). Each is stated as the question a trial would put; Section 6.4 states the outcome such a trial would carry.

**The perceiver’s own contribution.** Does making the perceiver’s contribution visible to him change what he credits the partner with? The candidate work locates the difference between what the partner put on record and what the perceiver supplied, with mentalisation-based treatment (Bateman and Fonagy, 2006) supplying the method of holding open what is in the other’s mind; whether marking which part of a belief was stated and which inferred reduces directional bias is untested.

**The attribution under procedure.** Does a procedure around the attribution do what insight alone is predicted not to? What is specific to the account is a test of carriage rather than of content: the perceiver says something determinate about his own state, notes the date, and looks for a trace of it in what the partner does weeks later rather than in the next reply. What that test returns is left open, and whether running it changes appraisal is the question.

**Readiness itself.** Does work on self-worth change readiness? Where being a person at all depends on being seen, needed or chosen by this partner, the configuration is fed from below, and schema therapy (Young et al., 2003) and trauma-focused approaches (van der Kolk, 2014) offer methods for rebuilding a self-worth not derived from relational function. That the three would have to run together is what the loop implies and not a finding.

What the account claims is narrow: the calibration of what a perceiver expects his partner to understand of him, and not traumatic bonding, whose affective and reinforcement dynamics run on their own terms (Dutton and Painter, 1981; Skinner, 1953). A perceiver can judge accurately what his partner takes in and remain bound to him, and a bond that persists is no evidence that over-attribution persists.

### 5.3 Prevention in early-stage relationships

Prevention would be the prospective use of Section 5.2 with perceivers whose partners have repeatedly met the responder condition, watching for the accuracy demand falling away as felt understanding rises (Section 3.2.3); it is as untested as the targets it rests on, and nothing in it shifts responsibility for a partner’s conduct onto the perceiver.

### 5.4 Boundaries of application and differential formulation

Four questions put to the perceiver alone route the presentation to one of five states. Is understanding low in both directions, his own included? Is the partner’s sparseness general, or confined to the perceiver? Do his attributions hold their shape across months? Was a correction ever available: explicit, literal, in a register he demonstrably reads, and followed by an occasion on which it could have shown?

Understanding that is low in both directions, the perceiver’s included, marks a problem of mutual legibility, and accommodation on both sides is the task. Milton (2012) locates such a breakdown between two differently disposed people rather than inside either one, and Mitchell et al. (2021) argue what it costs the party more often misread; in a registered report, information passed along mixed-neurotype chains as well as along matched ones while rapport still differed by chain composition (Crompton et al., 2025), so measured transfer and the experience of being met came apart along the line this account draws.

A partner whose inferences are sound and carried forward without being experienced as such presents unrecognized attunement, and treating that as this configuration aims the work at the wrong party; where his contributions cannot be judged, nothing is classified and a scorable occasion is sought. The two states that belong here are the two routes of Section 3.1, and uncertainty about a partner settles nothing either way: a perceiver who remains appropriately unsure can have a partner who meets the responder condition, and the account then applies to him. The questions stop there. The partner is absent, everything known about him comes from the party whose attributions this account calls uncalibrated, and the responder condition is entertained and never established; no formulation of him leaves the room.

Depressive disengagement, neurodivergent calibration of self-disclosure, and culturally formed reserve explain the same sparseness. Selectivity is the demarcation I propose against them, to be tested rather than relied on, and it has to show within a comparable communicative domain: a man who speaks in detail about his work and declines to formulate readings of anyone’s inner life is not selective in the sense the rule scores. Two distinctions hold whatever becomes of that rule: a partner who takes in what was said and then does otherwise has disappointed the perceiver without meeting the criterion, and repetition is weighed by what an utterance sets and not by its form, since a perceiver’s own words returned can be the most accurate contribution available.

The prevalence of the responder condition is unknown, and so are the sensitivity and specificity of the coding procedure of Section 3.1, so predictive values cannot yet be estimated (Meehl and Rosen, 1955); prevalence figures for individual characteristics do not establish the frequency of a dyadic configuration, and while both component quantities have been measured at scale (Reis et al., 2004; Crasta et al., 2021; Sened et al., 2017), their joint distribution has not. I therefore propose no cutoff and no decision rule, and none should be read into the instrument of Section 6.4.

The account can also be turned against the person it describes, since a partner’s depth recast as invention is an instrument of denial. Where there is violence, a reality test must not become a confrontation the perceiver has to survive.

Not classifying is a rule for the research design. In the consulting room the perceiver’s distress is the object of work whatever the partner’s standing, since what Section 5.2 asks about lies in what he supplies.

## 6 Discussion

### 6.1 Theoretical contribution

The claim I make for the IEE is maintenance. Over-attribution on the perceiver side is already described by the accuracy and bias tradition, and the operations of Section 3.2.1 add no term to it. What the configuration supplies is the condition under which such attributions stop being corrected, and two observables follow that the neighboring literatures do not record.

The responder condition of Section 3.1 supplies one. Empathic accuracy is scored occasion by occasion, so a responder wrong about the same content twice registers as two poor occasions rather than as a failure to carry a correction; the responder condition scores what happens between occasions, and no established scoring scheme extracts that quantity.

The accuracy demand supplies the other. Repair initiations, clarification requests, and disconfirmation probes leave a trace in the record, and path P2 predicts that their rate falls while felt understanding rises. Dyads in which the rate holds or rises while felt understanding rises, at the levels of readiness the account claims, are evidence against it, and no perception-based responsiveness instrument records the rate; observer-coded schemes have not been surveyed exhaustively here, so the claim is that the instruments this literature standardly uses do not extract the quantity, not that no scheme could be adapted to it. That rate and the recycling of the perceiver’s own material are given coding rules and denominators in Supplementary Material 1, Section 9.9.

The dyadic claim is decided elsewhere: Section 3.2.3 specifies cross-partner lagged paths whose removal returns the account to a moderated individual difference, and each carries the bound of Section 3.4 for a quantity that scales with a continuous predictor. The field module of H5 carries that test, and the non-decline of the surplus with it, on the specification of Supplementary Material 1, Section 14; the non-decline is decided by route (Section 3.4), and Section 6.3 states what the test reaches on each.

### 6.2 The IEE and generative systems

Assigning relational risk by product category, with a general-purpose assistant outside the account and a companion application inside it, does not survive the responder condition of Section 3.1: the same model can serve both, what a system does with a correction is a property of a deployment and a conversation, and the unit of assessment is the interaction. The discussion of AI companionship runs three questions together, whether the system delivers substantive information, whether it puts on record an understanding of this user determinate enough to be wrong, and what the user credits it with; a high mark on the first settles nothing about the second, the responder condition scores the second and the fate of the user’s corrections, and the discrepancy, where the probe can be run, is that score against the third.

What a system can infer about a user’s states could be measured by the procedure of Section 2.5, which this article does not adapt for deployed systems, and the criterion does not rest on it. A system that retains the user’s corrections and applies them falls outside the condition, and no appeal to the substrate reinstates it.

Treatments of machine memory collapse three horizons the criterion keeps apart: uptake in the next turn, where the content stands in the context of the exchange; return later in the session, which depends on retrieval; and continuity across sessions, which requires a store that outlasts the conversation and whose content is a matter of deployment rather than of architecture. The responder condition is aimed at the third: the criterion requires later, independent occasions and fixes them, for session material, as different sessions, the analog designs of H2 and H5 being the exception the manual fixes in advance (Supplementary Material 1). Storage settles nothing: a system that retains the user’s sentences across sessions can still carry no corrected attribution about him, and what he credits it with runs on none of the three clocks, one retrieved detail lifting it past anything the record supports.

The objection that presses hardest is that modern generative systems withhold nothing and produce depth in volume, which is fatal to a criterion built on self-disclosure and beside the point under the criterion of Section 3.1. A system producing determinate content of comparable intimacy about identified third parties, while setting determinate attributions about the user below the coded rate, meets the first route, and one re-asserting a corrected attribution on two later, independent occasions meets the second. A system that lets a correction lapse without re-asserting it is reached by neither route, a limit of the criterion and no verdict on the system (Section 6.3).

**What differs for the perceiver.** The criterion infers no motive in either case, so the coding does not mark the difference; a shared behavioral criterion does not make the generating mechanisms identical, and the difference lies there and in what the same record means to the perceiver and what he can do with it. A partner who sets an attribution again after correction has acted within a relationship that has a history, a future and a cost to him: the perceiver can put the correction to him again, read the lapse as information about the relationship, or leave. The same pattern from a system is a property of its deployment, of what is stored across sessions and retrieved, and says nothing about him; putting the correction again is not an act within a relationship that could answer for it. The criterion is held constant across the two cases for that reason, and no transfer of the loop is assumed: P1 and P2 have candidate counterparts on the user’s side, but their transfer is a hypothesis; the motivated recruitment of R1 is not attributed to a system; and the availability of prior input does not by itself establish the rise in cue recycling R2 predicts. The protective re-attribution of Section 3.2.2 reads a partner’s lapse as a wound or a hidden depth, and what it makes of a system’s lapse is open. With a partner, the carriage test of Section 5.2 is a question that can be put and his reasons enter the formulation (Section 5.1); with a system there are no reasons to formulate, and whatever work there is would address the attribution alone.

R3 has an evident analog in systems tuned to what users approve, and no claim about that is made here. Users of companion systems are characterized in terms close to those of Section 4: loneliness and attachment insecurity (Xie and Pentina, 2022); relationships described in romantic or familial terms and proceeding through stages (Pentina et al., 2023); harms following emotional dependence, including rupture when the system changed (Laestadius et al., 2024); and the support users describe drawing from them (Skjuve et al., 2021; Brandtzaeg et al., 2022; Ta et al., 2020). Two quantities follow for product assessment: the rate at which a system sets determinate attributions about the user, and whether explicit corrections survive into later sessions. Both are visible in interaction logs and neither requires a position on what the system understands (Shanahan, 2024).

### 6.3 Limitations

The article reports no primary data: the composite cases illustrate, and the targets of Section 5.2 are served by established methods untested against this configuration. The claim behind the responder condition is that calibration fails where a partner who sets such content elsewhere sets nothing about the perceiver that could be wrong, or sets it again after correction; the manual of Supplementary Material 1 is one candidate rule for scoring it, with untested anchors and thresholds. Should its indicators fail to cohere, or raters fail to agree, the rule is wrong and a different one is needed; the claim is then unscored and not refuted. What would refute it in the field is a coded condition, on whichever rule survives the coding study, that fails H1 or H5 (iv) on the conditions Section 3.4 states; the analog shared by H2 and H5 tests the mechanism on assigned counterparts of the two routes, and H5 (iii) within it can withdraw the construct under Section 3.4 whatever becomes of the manual.

The criterion reaches non-uptake in two forms and not in a third. A responder who sets determinate attributions freely, is corrected, and lets each correction lapse without ever re-asserting it is reached by neither route. Whether that pattern occurs is a question for the coding study, and if it is common the criterion needs a third route on the proportion of corrections not carried.

Exclusion of the reserved responder is an attempt at a boundary and not an accomplished one. The first route requires determinate content elsewhere on matters of comparable intimacy and the availability rule a correction the responder could read, both rules to be tested rather than guarantees; what counts as comparable intimacy is itself a coding decision, and a rival explanation of the pattern the gate scores is not excluded by it: a responder may speak readily about third parties’ inner lives and stay sparse about his partner’s because a norm of the relationship, or of the culture it sits in, reserves such readings for people who are not present. The gate cannot tell that apart from the configuration, and the comparison samples of the coding manual are where the difference has to show. The probe format shares that status: the original paradigm scores inferences about thoughts the target did not voice (Ickes, 1993), while the probe has the perceiver state the content in advance, an adaptation whose comparability is checked against a modified video-cued procedure, the standard one with its thought reports constrained to the probe’s form, and not against the unmodified paradigm (Supplementary Material 1, Section 9.5).

The probe has two further properties, and both would run against the account. It requests of the responder the accuracy that path R1 says goes unrecruited where it is neither requested nor consequential, so it may recruit accuracy not spontaneously displayed and reduce the observed surplus, most where the proposed Route-1 mechanism operates; and on the perceiver’s side, stating and locking contents of his own state during an episode is close to the first target of Section 5.2 and may interrupt the configuration it indexes. Neither is removed by design; what is fixed is what the design records, conduct under the probe set against conduct outside it in the same wave, and, before any test that carries a withdrawal consequence, evidence for the probe’s intended use, of which that comparability check is the necessary first part (Section 3.4; Supplementary Material 1, Section 9.5).

The maintenance claim is decided by route, and the two routes are not reached alike. Only the sensitivity analysis on waves with an assessable correction reads maintenance against a correction actually stated, and those waves come mostly from the second route, since a responder met on the first sets few attributions to correct; for the first route the primary estimate reads the course of a surplus that little disconfirmation has reached, which is weaker evidence of maintenance, and a route below the protocol’s minimum of dyads leaves the claim untested for it.

The maintenance loop is a model rather than a finding: the order in which P1, P2, R1 and R2 succeed one another has not been observed directly, and the hypotheses reach the two conditions and their consequences, not the sequence.

Cultural and gender variation in readiness, and in what counts locally as a determinate attribution, is unaddressed, and scope is limited to intimate partnerships and the conversational systems of Section 6.2.

### 6.4 Empirical programme and measurement development

The coding study comes first: the responder condition is not scored until a manual exists and two raters can apply it to unseen material and agree. The manual has to fix the anchor set for the specificity threshold, the rate below which the first route is satisfied, and the application of the availability rule; Supplementary Material 1 is a provisional manual fixing all three, and the corpus material raters would train on is built in the coding study. Recorded interactions from existing corpora, supplemented by sessions under the probe protocol of Section 3.5, are coded by raters blind to perceiver-side data, with reliability, the placement of the rate threshold and the behavior of the indicators as the targets. If the indicators do not cohere, or two trained raters cannot agree at a rate the field would accept (Supplementary Material 1, Section 11), the manual as written is wrong and nothing built on it is interpretable; the criterion of Section 3.1 is then unscored and not refuted (Section 6.3). Agreement is a condition of interpreting the code and not evidence that it measures the configuration.

The instrument is organized by content domain, with items and scoring in Supplementary Material 2. Readiness has two facets, the motivation to be understood and the threshold at which a contribution is taken to show understanding; they enter the designs of Section 3.4 separately and never as a composite, and the threshold facet is read off performance as well, so that readiness does not rest on self-report alone. Current appraisal records what the perceiver credits the partner with now and tracks change over treatment; retrospective recognition has a direction problem no wording repairs, since recognizing a past over-attribution requires the reappraisal the configuration prevents. The domains are scored separately, no total is formed, and no cutoff or case-identifying use follows (Section 5.4). Current appraisal is validated against the felt component of the probe of Section 3.5, which carries the construct; readiness is established on measurement grounds independent of the association H1 predicts, since that association is what H1 puts at risk; discriminant work against the ECR anxiety subscale (Brennan et al., 1998) removes attachment-anxiety variance by part correlation rather than leaving it inside a composite, and against the responsiveness instruments no discriminant claim is made (Section 3.3).

**What is specified here and what is not.** The coding manual is complete enough to be applied and Supplementary Material 1 carries it; the stimulus materials, the conversational task and prepared entries of the analog, the confederate’s script and the twelve vignette episodes of H3, follow as the appendix of a registered protocol, with the pilot that places the values Section 10 of that manual leaves open.

**Safeguards.** H4 is the only arm in which the scored record of what a responder missed is put in front of the perceiver, which, with the correction prompt of Supplementary Material 1, Section 8.1, is what Section 5.4 warns against where a relationship is unsafe. Screening for intimate partner violence and for coercive control at intake and again before every wave or observation period at the end of which that prompt may be put and before the release in H4, exclusion on a positive screen at intake from every arm in which a release is made and from the prompt, withholding of both from the point of any later positive screen, and debriefing of every perceiver who receives a release are set out in its Section 14, fixed before recruitment as part of the ethics application.

The therapy-outcome question carries a behavioral outcome: change in directional bias and in the rate at which the perceiver puts his readings up for correction, reported separately, since a discrepancy that closes because the partner improved is not the recalibration at issue.

## Data Availability

The original contributions presented in the study are included in the article/Supplementary material, further inquiries can be directed to the corresponding author.
