# Supplementary material for: The Interpersonal ELIZA Effect: a dyadic account of persistent over-attribution of understanding
Source: Front Psychol. 2026 Oct 21;17:1869060. doi: 10.3389/fpsyg.2026.1869060 (PMC13731227; doi:10.3389/fpsyg.2026.1869060)
Supplement: Supplementary file 1 [file Data_Sheet_1.docx]

# Supplementary Material S1

## Coding manual for the responder condition

Companion to: Riehm, A. The Interpersonal ELIZA Effect: A Dyadic Account of Persistent Over-Attribution of Understanding.

## 1. Purpose, status and limits

This manual states the rules by which the responder condition of Section 3.1 of the main text is coded from recorded interaction. Section 6.4 of the main text gives it three mandates: an anchor set for the specificity threshold, the rate below which the first route is satisfied, and the application of the availability rule.

Four limits govern everything that follows, and they are not disclaimers but coding rules.

**It is provisional.** Every numerical value in this manual is set by me and rests on no data. Section 10 lists them with the study and analysis that is to place each, and marks the values no study places: the equivalence bounds of 14.1, which are argued from clinical relevance before collection rather than estimated, and the values of 9.5 and 14.13 that the protocol names before collection. Until that study is run, a classification produced with these values is a research result about this manual and not a fact about a dyad. It is also one candidate rule and not the criterion itself: where this manual fails its own test in Section 11, the responder condition of main text 3.1 is left unscored and a different rule is needed, and nothing in that failure refutes the account; the analogue shared by main text H2 and H5 tests the mechanism on assigned counterparts of the two routes, and H5 (iii) within it can withdraw the construct under main text 3.4 whatever becomes of this manual.

**It has no diagnostic standing.** Prevalence is unknown, and so are the sensitivity and specificity of this procedure. No cutoff, decision rule or case-identifying use follows from it, and none should be read into it (main text, Section 5.4).

**It codes contributions, not persons.** No code in this manual names a kind of person. Every code attaches to a contribution within an interaction. Raters do not record, and are not shown, the neurotype, culture, temperament, diagnosis or presumed motive of either party.

**It infers no motives.** No coding decision may rest on what a rater takes a responder to have intended, wanted or felt. Where a rule seems to require that, the occasion is not assessable.

## 2. Standing rules

**2.1 What never enters a criterion.**

| Quantity | Standing |
| --- | --- |
| What the responder discloses about himself | Covariate of discoverability. Recorded under 9.7. Enters no route, no gate, no rate. |
| How much the responder speaks | Recorded. Enters nothing. |
| Whether the responder agreed with, accepted or acted on what he read | Enters nothing. |
| What the perceiver feels about being understood | Never shown to the rater. Enters nothing. |
| The responder’s communication style, register or fluency | Enters nothing except through 4.3, which is symmetric across styles, and through V3 of 6.2, where the register the responder demonstrably answers in gates the availability of a correction. |

**2.2 Not assessable is not a low value.** A contribution or occasion the record does not let a rater judge is coded NA. It leaves both the numerator and the denominator of every rate. It is never entered as a zero, a low grade or a failure. Failure to show understanding is not shown failure to understand.

**2.3 Repetition is scored by what it sets.** Wording the perceiver supplied can carry an attribution that could be wrong, and it can carry none. The form of an utterance decides nothing; rule 4.2 decides.

**2.4 Acknowledgement is not failure.** A contribution that registers what the perceiver has said, without adding an attribution, is coded D-. The occasion it falls in stays assessable and enters the denominator of 5.4 like any other, since the unit of the rate is the occasion and not the contribution (3.3). The contribution is not evidence against the responder and carries no separate mark.

## 3. Units

**3.1 Content.** A content is a statement by the perceiver about his own state, in the first person, naming one state and what it is about, and tied to an occasion or object. “I was frightened rather than angry when you went quiet last night” is a content. “I have had a hard week” names no state and is not.

**3.2 Contribution.** A contribution is one party’s uninterrupted stretch of speech or one sent message. A stretch is ended by the other party taking the floor with anything beyond a back-channel (“mm”, “right”, “yes”), or by a silence of ten seconds or longer. Written messages sent by the same party within sixty seconds of one another are one contribution. *Provisional; see Section 10.*

**3.2a Encounter.** An encounter is a continuous period of contact between the two parties, opened by the first contribution of either and closed by six hours with no exchange, or by the parties’ own departure where that is recorded. It can hold several occasions. The term matters because 3.5 counts encounters as well as occasions, and because a dyad can reach fifteen assessable occasions inside a single long evening, which is what the three-encounter requirement exists to exclude. For chat and other written exchange the same six-hour rule applies, so an encounter is a continuous thread of messaging rather than a calendar day.

**3.3 Occasion.** An occasion opens at the perceiver’s contribution carrying a content and closes at the earliest of three things: the sixth subsequent responder contribution, six hours of no exchange, or the perceiver’s next contribution carrying a content. The third is the ordinary case in conversation and is decided here rather than left open: a new content closes the running occasion at the last responder contribution before it and opens a new one, so that no responder contribution is counted in two occasions and no content shares its window with another. The sixth responder contribution lies inside the window it closes and is coded like the five before it. Every responder contribution inside that window is coded. The occasion, not the contribution, is the unit of the rate in Section 5.

**3.4 Assessable occasion.** An occasion is assessable where all five hold. Q1: the perceiver’s content meets 3.1. Q2: the record of the window is complete enough to read every responder contribution in it. Q3: at least one responder contribution falls inside the window. Q4: the exchange is not suspended (an agreed period of reduced contact, seven days or longer, recorded with its dates). Q5: no contribution in the window is left D? under 4.2. No exception is made for windows in which some other contribution is D+, because such an exception would make assessability depend on the outcome: windows would survive only when they contained the very thing the rate counts, which biases the rate upward and works against a finding of Route 1. Where any of the five fails, the occasion is NA and drops out under 2.2. Q1 to Q5 are the only grounds on which an occasion is NA.

**3.5 Minimum material.** A dyad is not classified on fewer than fifteen assessable occasions across at least three separate encounters, and they must be spread across the observation period rather than clustered in one phase of it. Spread is decided arithmetically and not by impression: the period is divided into three equal intervals by date, at least one assessable occasion must fall in each, and no single interval may carry more than half of them. Below either requirement the dyad is reported as not classified for want of material, unless Route 2 is met on the material available: the minimum exists so that the rate of 5.4 can be estimated, which is what Route 1 needs, and line 2 of Section 7 accordingly decides for a dyad whose record establishes the second route, with the shortfall reported alongside the outcome. *Provisional; see Section 10.*

**3.6 Window, session, turn.** Three words the manual uses, defined here.

A **window** is the span of an occasion: from the perceiver’s contribution carrying the content to the close fixed in 3.3. Every responder contribution inside it is coded, and Q2, Q3 and Q5 of 3.4 are questions about it. The word carries no other sense in this manual; where an observation period is meant, 3.5 and 9.3 say period.

A **session** is one sitting. In natural material it is the encounter of 3.2a and the two words are interchangeable there; the manual prefers encounter. In the analogue of 9.8 it is the experimental sitting, which holds all three condition blocks of a participant and is therefore larger than any of them. In the coding study it is a recording appointment. Which of the three is meant follows from the design under discussion, and no rule turns on the word.

A **turn** is the responder’s next contribution under 3.2 after a given perceiver contribution. It is what 6.5 excludes when it says uptake is persistence across occasions and not echo in the next turn, and it is the unit S2.3, rule 9, excludes for the same reason. A turn is never an occasion, and no L is ever a turn.

## 4. The specificity threshold

This is the first mandate of main text 6.4.

**4.1 The question.** Does this contribution set a proposition about the perceiver that the perceiver could reject on grounds of fact about himself?

**4.2 The test.** A responder contribution is **D+** where all three hold, and **D-** where any fails.

- **(a) Bearer.** It attributes a state, disposition, experience or reading to the perceiver, not to the world, not to the responder, not to people in general. Where the wording names no bearer but stands directly after a content of the perceiver’s and refers back to it, position alone does not fix the bearer: the contribution is D? unless the wording itself carries a pronoun, a possessive or a repeated noun tying it to him. A remark about a state in the abstract, such as “that kind of pressure builds”, is not about him because it follows something he said.
- **(b) Falsifiable.** The perceiver could answer “no, that is not how it was” on grounds of fact about himself; a different perceiver in the same exchange could truthfully give a different answer; and his rejection would be more than a contradiction of something he has himself just asserted within that occasion.
- **(c) Asserted, not reported.** The responder puts it forward as his own reading of the perceiver, rather than reporting, quoting or querying what the perceiver said. “You said you were frightened” reports. “You were frightened, not angry” asserts.

Determinacy is coded independently of novelty. A contribution can be determinate while adding no new content: on a later occasion, restating a reading the perceiver himself supplied earlier is an assertion the responder now makes in his own voice, and the perceiver can reject it. What clauses (b) and (c) exclude is different: an echo inside the same occasion, which the perceiver could reject only by contradicting himself, and a report of his speech, which asserts nothing. Whether an attribution is new, whether it is accurate, and whether it is used on later occasions are three further questions, settled by 4.4, by Section 9 and by Section 6 respectively, and never by 4.2.

Where a rater cannot decide (a), (b) or (c) from the record, the contribution is D?. Q5 of 3.4 then decides the standing of the occasion; 4.2 sets no assessability rule of its own.

**4.3 Anchor set.** Six graded examples, each following a perceiver content of the form “I felt small at dinner after what your father said about my job.” The content names a state and what it is about, as 3.1 requires; a description of conduct such as “I went quiet” names no state and would not meet Q1.

|  | Responder contribution | Code | Why |
| --- | --- | --- | --- |
| 1 | “Mm.” | D- | Sets no proposition. Fails (a). |
| 2 | “You are right to feel like that, anyone would.” | D- | Ratifies a position and sets no proposition about his state. Fails (a). |
| 3 | “You are someone who needs to be taken seriously.” | D- | True of nearly anyone; no different perceiver could truthfully reject it. Fails (b). |
| 4 | “You felt small after what he said about the job.” | D- | Returns the content the perceiver has just stated and adds nothing. Within the same occasion he could reject it only by contradicting himself, so it is not falsifiable by him. Fails (b). |
| 5 | “I think it landed as him saying you had settled for less, not as a remark about the job.” | **D+** | Asserted as the responder’s own reading, tied to this occasion, and the perceiver could say it is wrong on the facts. |
| 6 | “You go quiet with him the way you do when you have decided arguing will cost more than it is worth.” | **D+** | Asserts a disposition holding across occasions, which the perceiver could reject on the facts. |

Anchors 4 and 5 are the threshold pair. What separates them is clause (b), and no difference in warmth, length or grammatical form does. A seventh case is worth holding beside them: on a later occasion, and with the matter again in play, “you were frightened then, not angry” is D+, because the responder now asserts it in his own voice and the perceiver could reject it on the facts. Novelty is not what makes an attribution determinate.

**4.4 Grading and novelty, both recorded and neither criterial.** Above the threshold, a D+ contribution is described by the number of independently falsifiable components it carries (state, ground, connection). Separately, each D+ contribution is marked *new* where its content appears nowhere earlier in the perceiver’s own record, and *restated* otherwise. Both are recorded for description only. Neither enters a decision, neither carries a reliability target, and neither is accuracy, which only Section 9 measures.

**4.5 Why competent reflective practice is D+.** A practitioner who works by reflection states an inference and offers it for correction. Anchor 5 is a reflection. The approach requires that empathic understanding be experienced and communicated (Rogers, 1957), and Rogers separated that requirement from any verbal form in the same paper: reflecting feelings is no essential condition of therapy, and a feeling can be reflected in a way that communicates a lack of empathy. This manual therefore scores what a reflection sets, not that it is a reflection. Competent practice produces D+ contributions at a high rate and carries corrections forward, and so meets neither the first route, on the rate, nor the second, on carriage. It falls outside the responder condition by the criterion and not by exemption.

## 5. Route 1: determinate content elsewhere at comparable intimacy, attributions about the perceiver below the rate

**5.1 The two parts and the three values.** Route 1 is not an absence of attribution; it is a rate below a threshold in a responder who demonstrably produces such content elsewhere. It takes exactly three values.

- **Met**: the gate of 5.3 is passed and the rate of 5.4 lies below the threshold.
- **Not met**: the rate lies above the threshold, whatever the gate. A responder who sets determinate attributions about the perceiver at that rate is not in this route.
- **Not decided**: the rate lies below the threshold but the gate is not passed, or the rate falls in the margin of 5.4, or either cannot be read from the record.

The middle case of *not decided* is the one the gate exists for. A responder whose determinate content is sparse throughout, and who therefore never reaches comparable intimacy about anyone, is not thereby placed outside the responder condition; he is not classified on this route, and the second route is assessed independently.

**5.2 The intimacy ladder.** Applied to the subject matter of a contribution, whoever its bearer.

|  | Level | Example subject matter |
| --- | --- | --- |
| I0 | Facts and logistics | where someone lives, what a job involves |
| I1 | Preferences and opinions | what someone likes, what he thinks about a film |
| I2 | Reactions to events | that someone was annoyed by a delay |
| I3 | Enduring states, vulnerabilities, conflicts. A pattern of conduct named as a pattern reaches I3 where the contribution ties it to an enduring state, and stays at I1 where it reports the conduct alone | what someone carries, fears, cannot say, is like inside |

**5.3 The selectivity gate.** The gate is passed where the responder produces, **in the dyadic record itself and addressed to the perceiver**, about **identified third parties** (persons other than the perceiver and other than the responder himself), at least three contributions on at least two separate encounters under 3.2a that would be D+ under 4.2 if the bearer were the perceiver, and whose subject matter reaches the comparison level. The comparison level is the highest intimacy level the perceiver has reached about himself in the same observation period, and never below I2, which every content admissible under 3.1 already reaches by naming a state and what it is about.

Three consequences follow.

- Determinate talk about work, facts or opinions does not pass the gate where the perceiver has been speaking at I3. The comparison is fixed by what the perceiver offered, not by a constant, so it is calibrated within the dyad.
- A responder who speaks in detail about many things and never formulates a reading of anyone’s inner life does not pass the gate, and Route 1 does not reach him.
- The responder’s statements about himself never pass the gate. Self-disclosure is a covariate of discoverability and enters no criterion (main text, Section 3.1).

Where the gate cannot be decided from the record, Route 1 is not assessable and the second route is assessed independently.

**5.4 The rate.** Over the assessable occasions of 3.4, let *n* be their number and *k* the number on which at least one responder contribution is D+. The rate is *r* = *k*/*n*.

Route 1 is **met** where the gate is passed and *r* is at most *t* - 1/*n*; **not met** where *r* is at least *t* + 1/*n*; **not decided** in between, which is the third value of 5.1 and is a value of the route, not the outcome of Section 7. The margin of one occasion is a granularity rule, not a statistical model.

The threshold is *t* = 0.20. *Provisional; see Section 10.* Every result is reported again at 0.10 and 0.30.

## 6. Route 2: a disconfirmed attribution not carried forward

This section applies the availability rule, the third mandate of main text 6.4.

**6.1 The triple.** Route 2 is assessed on triples of: an attribution **T** the responder set about the perceiver; a correction **K** the perceiver addressed to the responder; and a later occasion **L** on which the content of T was in play.

**6.2 The availability gate.** A triple is scored only where all four hold. Where any fails, the triple is not assessable and is dropped; it is never scored against the responder.

- **V1 Explicit and literal.** K states in plain words that the attribution is wrong and what is the case instead. Irony, implication and displays of feeling do not qualify.
- **V2 Determinate target.** T was D+ under 4.2, so there was a proposition to correct.
- **V3 Readable channel.** K was delivered in a channel and register in which the responder has demonstrably answered on the content of what was said on at least two dated earlier occasions. *In the analogue of 9.8 this gate is met by protocol and not by record:* the confederate answers on content at every marked point by instruction, so the channel is readable by construction, and V3 is recorded as met without the two dated instances that field material has to supply. A correction stated in segment 2, which no earlier segment could support, is therefore assessable there.
- **V4 In play.** On L, the content of T arose: either party referred to the situation, state or person T was about. An occasion on which the matter never came up is not a test and is dropped.

**6.3 Coding L.** Each qualifying L receives exactly one mark, applied in this order:

- **Re-asserted**: some contribution restates T in its uncorrected form.
- **Carried**: otherwise, some contribution addresses the matter of T and applies the corrected content to it, either as a D+ consistent with the corrected version or as a question whose falsifiable content rests on it.
- **Cited**: otherwise, some contribution reproduces the corrected wording and applies nothing of its own to the present matter.
- **Neither**: nothing on that occasion falls under the marks above. It is neither carriage nor re-assertion. It counts towards a correction being not carried under 6.4, but it can never by itself bring Route 2 about, because 6.4 also requires a re-assertion. Non-production across all assessable occasions is what Route 1 scores.

**6.4 The decision, per correction then aggregated.** For each correction with at least one qualifying L: it is **not carried** where at least one L is *re-asserted*, or where no L is *carried*; it is **carried** where at least one L is *carried* and none is *re-asserted*. A correction with no qualifying L is not assessable.

**The repetition requirement.** Route 2 is **met** only where at least two re-assertions are documented on separate qualifying occasions: either the same corrected attribution is re-asserted on both, or each of two distinct corrected attributions is re-asserted once. The two limbs are symmetric, and each rests on an observed repetition rather than on an absence of one. Route 2 is **not met** where at least one correction is assessable and that requirement is not reached. Route 2 is **not assessable** where no correction is assessable.

An occasion marked *cited* or *neither* records that the responder set no determinate attribution about the perceiver there. It counts towards the correction being *not carried*, which is the descriptive summary of that correction’s course and is reported as such, and it counts towards neither limb of the repetition requirement, because the absence of a demonstration is not a demonstrated repetition. A correction whose later occasions are all *cited* or *neither* therefore contributes nothing to Route 2 being met. This is what divides the two routes: re-assertion is what Route 2 scores, and Route 1 scores the rate at which determinate attributions are set at all, and the same material is not counted twice.

The counts of corrections, of qualifying occasions and of each mark are reported whatever the outcome. A rule resting on a single failure would otherwise reward responders who are corrected more often, and the raw counts let a reader see how much material each decision rests on.

**6.5 Later and independent.** For a relationship, L is later and independent of K where it falls on a different occasion under 3.3 and at least seven days after K. The interval is in days and in nothing else. A count of intervening contributions does not substitute for it, because twenty contributions can fall in one evening, and what the rule exists to exclude is exactly that: a single phase of an interaction establishing the second route. For session or chat material, where exchanges are dense and dated, L must fall in a different session from K, that is after a break the parties themselves treat as ending the exchange, and never merely later in the same session. In a single-session analogue of the kind H2 uses, the separate segments of a condition block are the occasion-equivalents (14.3a), provided each is closed by an intervening task that ends the exchange and the protocol fixes this in advance; without such separation a single session yields no L at all. This is a manipulation convention for an experiment in which the responder’s conduct is assigned, and it codes nothing in natural material: outside such a design, and for any deployed system, a return later in the same session is never an L. *Provisional; see Section 10.* An echo within the same occasion is never an L: uptake is persistence across occasions, not echo in the next turn.

**6.6 Citing is not carrying, applying is.** A responder who reproduces the corrected wording as a report of what the perceiver said, and asserts nothing of his own about him, is D- under 4.2(c), and the occasion is marked *cited*. A responder who brings the corrected version back and applies it to the matter in hand has *carried* it, whether as a statement or as a question with falsifiable content. That is the ordinary form in which a retained correction returns, in a relationship and in a system alike, and a system that returns corrections in it falls outside the second route.

## 7. The decision, in order

Applied in this order, stopping at the first line that decides.

1. The minimum material of 3.5 is not met **and** Route 2 is not met or not assessable on the material available (6.4): **not classified**. The minimum exists so that a rate can be estimated, which is what Route 1 needs; Route 2 rests on documented re-assertions and not on a rate, so a dyad whose record establishes it is classified on that route even where the rate is not estimable, and the shortfall is reported with the outcome.
2. Route 2 met (6.4): **responder condition met, second route**.
3. Route 1 met (5.1): **responder condition met, first route**.
4. Route 1 not met (5.1) **and** Route 2 not met or not assessable: **responder condition not met**.
5. Anything else: **not classified**. This is where a responder lands whose determinate content is sparse throughout, so that Route 1 is *not decided* for want of a passed gate and no correction of his has been available to test.

The order matters and is fixed. Route 2 is tested first because it rests on documented re-assertions rather than on an estimated rate, so it can decide on material too thin for Route 1, and because a responder who re-asserts a disconfirmed attribution is producing determinate content about the perceiver, which the first route’s rate would not capture. Both route values are recorded on the sheet whatever the outcome, so no distinction is lost by stopping early.

There are three outcomes and no others: **met**, **not met**, **not classified**. Not classified is not a weak form of met.

**What “not met” asserts.** It asserts that the condition was not established in the material observed, and nothing more. Line 4 is reached where the rate settles the first route and the second route is either not met or not assessable, and a route that could not be tested for want of an available correction is not thereby disproved. The route values and the counts behind them are reported alongside every outcome, so that a dyad in which twenty corrections were carried is distinguishable in the record from one in which none could be tested. Where the distinction matters to an analysis, it is made on those values and not on the outcome label.

## 8. Elicitation

Occasions are created rather than awaited, so that a dyad in which the perceiver says little is not left unclassifiable by his own reticence.

1. **Trigger.** Fewer than three spontaneous contents meeting 3.1 across the first four encounters. An occasion coded NA under Q3 is not a trigger: Q3 fails where the responder does not contribute, and asking the perceiver for more content does not repair the responder’s silence.
2. **Instruction, read verbatim.** “As it comes up, and in your own words, say one thing about how you are, in one sentence. Say what state you are in and what it is about, so that it would be possible for someone to get it wrong.”
3. **Form check.** The rater accepts the content only where it meets 3.1.
4. **Channel.** The content is delivered in a channel that satisfies V3. Where none does, elicitation is deferred and the dyad is reported as not classified for want of a readable channel.
5. **Mixing.** A content counts as elicited only where it follows a logged prompt in the same encounter; the instruction of step 2 is read once and does not make every later content elicited, and contents produced without a prompt are spontaneous however often the perceiver has been prompted before. Where the two are mixed, 3.5 applies to the total, no more than two thirds of the assessable occasions may rest on elicited contents, and the split is recorded. The cap and the trigger have to be read together: the trigger fires below three spontaneous contents in four encounters, and the cap then requires at least five spontaneous contents among fifteen assessable occasions. A dyad that reaches neither is reported as not classified for want of material, which is the honest outcome and not a failure of the elicitation.
6. **Non-contamination.** The perceiver is not told what is being scored, and he is never asked to notice a discrepancy between what he feels and what the record holds.

**8.1 Elicitation of a correction.** Section 8 creates occasions on which an attribution can be set; it does not create the corrections the second route is coded from, and without one that route is not assessable (6.4). The attribution is not shown to him and he is not asked to compare anything. What is put to him is a single open question, asked whether or not any attribution was set: “Did he say anything about how you were that was not right?” It is put at the end of each observation period, which is the wave of 14.13 in the field module, the coding block in H1, the first block of 14.7 in H4, and the observation period of 3.5 in the coding study and in any other observational arm; the analogue of 9.8 elicits its correction under 9.8b instead, and this position does not run there. A reply naming a matter of fact about himself is what triggers the prompt below; a reply that names no such matter triggers nothing and is recorded as a nil return. This is deliberately not the discrepancy question the account forbids: it asks whether one attribution about him was false, which is a judgement about a single proposition, and not how far he was understood, which is the standing appraisal; and it never sets a scored entry against his own record. That the first can be asked without moving the second is an assumption of the design and not a finding, and it is marked here as one: the answer is recorded at every period, and the occasions after a period at which a non-nil answer was given form their own stratum beside the prompted-correction stratum, so that any movement the question produces is visible in the result instead of being assumed away. Where a reply names such a matter and no correction has been stated to the responder within four weeks, the perceiver is prompted once for each D+ contribution the rater matches to the matter he named, the matching being the rater’s work on the record alone and never put to him: “If anything he said about how you were was not right, say so to him in your own words, plainly and not as a hint.” The prompt is never put to a perceiver whose dyad screened positive under 14.9, at intake or at any later assessment; from that point this position supplies no occasions and the second route rests on spontaneous corrections alone. The occasions so withheld form their own reported stratum, and the reason matters: where such a dyad reaches *not classified* for want of an assessable correction, that outcome records a safeguard of this protocol and not a property of the dyad, and it is never pooled with dyads whose record is thin because the responder said little. The withheld count is reported by route, and no participant is coded adversely for an occasion the protocol declined to create. The prompt is recorded, the correction is coded under 6.2 like any other, and occasions following a prompted correction are flagged and analysed as a separate stratum, since a correction the protocol asked for is not one the perceiver produced unprompted. Where the perceiver declines, that is recorded and nothing is inferred from it. This step exists because the account’s own path P2 predicts that the rate of spontaneous corrections falls exactly where the responder condition holds, so without elicitation the second route would be least assessable where it matters most. It reduces that dependence and does not remove it, and the reason is stated rather than left to be found. The question runs through the perceiver’s own judgement that a reading of him was wrong, and main text 3.2.2 predicts that such a reading is commonly absorbed rather than registered where the configuration holds, so a nil return is itself what the account expects there. Where the responder condition holds, the second route is therefore the harder of the two to assess by the account’s own lights; the rate of nil returns is reported by level of readiness and by route, and reported beside the sensitivity analysis of 14.13 on waves with an assessable correction. At the level of classification, the number and proportion of dyads whose classification on the second route depends on prompted corrections, that is, dyads that meet the route on all documented re-assertions and would not meet it on their spontaneous corrections alone, is reported with the wave, or block, at which each was classified, split into those classified on prompted corrections only and those on a mixture, since the elicitation can create the occasions on which a dyad is classified; where that proportion is large it is stated as a limit on what the coded condition reflects, and the estimates of 14.13 are reported again without the dependent dyads as a sensitivity analysis (14.13).

## 9. The demonstration probe

The probe supplies demonstrated understanding for the analyses of main text 3.5. It is scored separately from Sections 4 to 7 and enters no route, no gate and no rate.

**9.1 Order, never reversed.**

1. An episode is one continuous interaction of at least ten minutes. Marked points are fixed by the person preparing the material, independently of both parties, and both parties receive the same list.
2. As the episode runs, the perceiver records privately and in writing, at the marked points, up to three contents of his own state, one sentence each, time-stamped and meeting 3.1; a content not of that form is excluded from scoring by the person named in the protocol under 9.5, who has not seen the responder’s entries, and the number excluded is reported. Each is locked as it is written and cannot be revised afterwards. **Both parties write at every marked point**, the responder writing out the time and a fixed neutral line supplied on his sheet, and the perceiver writing the time and the same line at every marked point at which he records no content, a task fixed here rather than left to the protocol, so that the act of writing carries no information about which points bear a content; without this the visible writing would tell the responder exactly where he is about to be scored, and step 3 could not be run blind. This is the adaptation the main text names: the content is stated beforehand, not reconstructed after the fact.
3. Within two minutes of the end, the responder records, without access to that record and without being told which points carry a content, what he took the perceiver to be conveying at each point.
4. The perceiver then rates each of his own contents on the scale of 9.2, put to him in the form of 9.2a, for how far he takes the responder to have grasped that content. This is felt understanding, scored over the same contents and on the same scale as the responder’s record, which is what puts the two quantities on one metric. A separate global rating of how understood he felt in the episode is taken alongside and is never substituted for it. All of it is sealed and never reaches the rater.
5. Raters blind to readiness, to every understanding rating and to group score each responder entry against the matching perceiver content. Entries at points carrying no perceiver content are unmatched and enter no score.

Where the responder had any access to the perceiver’s record, the occasion is NA. The criterion is the perceiver’s own report, collected separately and withheld from the person inferring (Ickes, 1993; Ickes et al., 1990).

**9.2 The three-point scale.** One scale for both quantities, over the same contents. Every content of 3.1 names two things, a state and what that state is about, and the scale asks how many of the two the entry corresponds to. The values are applied in the order 2, then 1, then 0, and the first that fits is the score.

- **2** Both the state and what it is about correspond to the record, in kind and in degree, allowing for wording.
- **1** Exactly one of the two corresponds in kind, or both correspond in kind but not in degree. Three shapes: (a) state caught, ground missed; (b) ground caught, state missed; (c) both right in kind, one or both wrong in degree. Degree is judged only on an element that corresponds in kind, so where one element is missed the degree of the other does not change the value.
- **0** Neither the state nor what it is about corresponds. This is the residual value and is scored only where 2 and 1 do not fit.

Two dimensions and the degree distinction generate five cases, which map onto the three values as 2, 1, 1, 1 and 0, and no case fits two of them. The degree judgement is made first and the ordering rule applied afterwards, so that a case of right in kind and wrong in degree cannot be taken as 2 by reaching that value first.

Degree is judged against the record’s own words and not against the rater’s sense of intensity: a difference of degree is scored where record and entry name states a competent speaker would not use interchangeably of the same episode, and a difference of wording alone is not one. Three boundary pairs, against the record “I was frightened about the examination”. The entry “he was terrified about the examination” is right in kind on both and overshoots the state in degree: **1(c)**. The entry “he was afraid about the examination” differs in wording and not in degree: **2**. The entry “he was frightened about the meeting with his manager” catches the state and misses the ground: **1(a)**. The entry “he was irritated about the meeting with his manager” corresponds on neither: **0**.

**9.2a The perceiver’s form of the scale.** The perceiver never sees the responder’s entries, before or after his own rating; that is what keeps the two judgements independent, and it means the scale cannot be put to him in the terms of 9.2, which scores an entry against a record. It is put to him in the terms of his own content, and the instruction is read verbatim: “For each thing you wrote down, say how much of it he got. **2** if he took in both what you were feeling and what it was about, and got the strength of it about right. **1** if he took in one of the two and not the other, or got both but pitched the strength wrong. **0** if he took in neither.”

The two forms score the same two elements and the same degree distinction over the same contents, which is what puts felt and demonstrated understanding on one metric. The degree case matters more than it looks: without it the perceiver would score “terrified” against his own “frightened” as a full 2 while the rater scored the entry 1(c), and the difference would appear as directional bias produced by the scale form rather than by the dyad, in the direction the account predicts. That artefact is the reason both forms carry it. They are not the same judgement, and the manual does not pretend otherwise: the rater scores a record against a record, the perceiver scores a person from the whole episode, and the difference between the two is the quantity the account is about. The perceiver’s global rating of how understood he felt is taken alongside under 9.1 and is never substituted for the content-level ratings. The same instruction and the same three values are used at step 5 of 9.8.

**9.3 Sampling, and why it differs by design.** In an analogue design the prepared responder entries are written to span the range, so that each condition is expected to yield entries at 0, 1 and 2; the obtained scores are measured rather than assumed, because the perceiver’s content is free, and no sampling depends on what is observed. In a field design the observation period is fixed in advance instead, by dates or by a number of episodes, and sampling never continues until a wanted score appears, because a stopping rule that reads the outcome would decide the observation size by the result. Where a dyad’s episodes yield too little variation in demonstrated understanding, its within-dyad tracking slope is reported as resting on too little within-dyad variation to be read on its own. Under the mixed model of main text 3.5 a shrunken random-effect prediction exists for every dyad regardless, and that prediction is not reported as a dyad-level estimate. It is not repaired by seeking further episodes until a low score is found.

**9.4 Criterion error.** The perceiver’s record is obtained independently of the inference and is not thereby infallible, since targets misremember and edit. Any content added after the lock, edited, or marked uncertain is flagged with its reason, and analyses are run and reported with and without flagged contents.

**9.5 Adaptation, stated as such, and what the probe does to what it measures.** The original paradigm scores inferences about thoughts the target did not voice (Ickes, 1993). Here the perceiver states the content in advance. That is a departure, it may raise correspondence scores relative to a procedure in which the content is reported afterwards, and evidence for the probe’s intended use is an outstanding requirement, of which the comparability criterion stated below, read against a modified video-cued procedure, is one necessary part. Two further properties of the probe are demand and reactivity, and neither is removed by design. *Demand on the responder.* Path R1 of main text 3.2.3 holds that accuracy about the perceiver goes unrecruited where it is neither requested nor consequential; the probe requests it, so what 9.1 scores is elicited inferential accuracy and not the accuracy the responder habitually brings to the relationship. The elicitation may recruit accuracy that is not spontaneously displayed and so reduce the observed surplus, most where the proposed mechanism of the first route operates, since that is where least is otherwise requested of him; the direction is derived from the account and not observed, and so far as it holds it runs against the hypothesis, as the bias of 9.6 does. *Reactivity on the perceiver.* Stating and locking contents of his own state during an episode is close to the first target of main text 5.2 and may interrupt the configuration it is meant to index; that bias runs the same way. *What is recorded.* In the field module of 14.13 two rates are computed twice, once on the probe episode of the wave and once on the wave’s other recorded interaction, and reported side by side: the rate at which the responder’s spoken contributions set a determinate attribution about the perceiver, counted per contribution under 4.2, and the accuracy-demand rate of 9.9. The decision of Section 7 and the rate of 5.4 are taken on the cumulated material of 14.13 alone and are not computed per arm, since a single episode cannot reach the minima of 3.5. The comparison is descriptive and is not the comparability study below: the raters of Section 11 code both kinds of material and are not blind to which is the probe episode, agreement is reported for each kind under 11.3, and a difference between them is reported beside each route’s result for (iv); the comparison does not alter the interval-based reading of (iv), while construct-level interpretation remains subject to the requirements below. In the analogue of 9.8 every block is probed and no such comparison exists, which is one reason the field module is carried. *Comparability with a modified video-cued procedure.* Before the confirmatory collection for H1 and H5 begins, and so before any test that carries a withdrawal consequence under main text 3.4 is read, the probe is compared with the video-cued procedure of the standard paradigm on a sample of dyads that takes no part in that test, of a size named in the protocol before collection (Section 10) and not extended once a result is seen (9.3). Each dyad supplies the same number of episodes under each procedure, fixed in the protocol, in counterbalanced order: under the probe as 9.1 runs it, and under the reference procedure, in which the perceiver reviews the recording afterwards and reports at self-chosen points and the responder infers at those points. The reference arm is modified in one respect: the perceiver is instructed to state each thought as a content of the form of 3.1, a state and what it is about, so that both arms yield entries the scale of 9.2 can score. That instruction moves the reference arm towards the probe, and the comparison is therefore stated as one against a modified video-cued procedure and not against the unmodified paradigm of Ickes (1993); the main text names it in those terms. Contents not of that form, as judged by a person named in the protocol, blind to arm and before the pairs are formatted, are excluded from the comparison and their number is reported by arm; the same judgement is applied to probe contents in confirmatory use under 9.1, so that the comparison assesses the probe as the confirmatory arms use it; and where the proportion excluded differs between the arms by more than a value named in the protocol (Section 10), that comparison is not read and counts as one in which the criterion was not met. Both arms are scored by the same raters under 9.2, blind to procedure: what a rater scores in either arm is a written perceiver content set against a written responder entry, and the pairs are presented in one format carrying no mark of the procedure; each rater also records, for each pair, which arm he takes it to come from, and the agreement of those guesses with the truth is reported as the check on the blind. The perceiver and the responder are not blind to the procedure they are in, which cannot be helped and is stated. The difference in correspondence between the procedures is a model-implied expectation on the content level under 14.11, with the dyad as a random effect, read on the comparability bound of 14.1, 0.15 points, half the contrast bound, and reported again at 0.1 and 0.2 points. Three outcomes follow from the first comparison: the comparability criterion is met where the 90 per cent interval on that difference lies entirely inside the bound; where the interval lies entirely beyond it, the criterion is not met, the probe is revised once, the revision is recorded, and the comparison is run once more on a fresh sample of the same size; where the interval covers the bound, the criterion is not met, and the same single revision and second comparison follow; where no revision is undertaken, the consequences of an unmet second comparison apply. The second comparison closes the question for the study: where its interval lies entirely inside the bound the criterion is met; where it lies beyond the bound or covers it, the criterion stands unmet, no third comparison is run under these rules, the probe carries no withdrawal consequence in that study, and every reading of H1 and of H5 (iii) and (iv) is reported as a finding about the procedure and the mechanism as not adequately tested, whichever way it runs. Further development of the probe is a separately specified later step with its own registered comparison, not a repetition under these rules. *What meeting the criterion does and does not establish.* It establishes that stating the content in advance does not shift correspondence by more than the bound on average. It does not by itself establish that the probe indexes the calibration a dyad habitually shows, that the individual departures between the two procedures are small, or that demand and reactivity are negligible; those remain assumptions of the design, stated above, and construct-level interpretation rests on evidence that has not yet been collected. The criterion is a necessary part of the evidence main text 3.4 requires for the probe’s intended use and not the whole of it. The additional evidence required for the probe’s intended use, the assessment of demand and reactivity among it, is specified in the protocol before collection, drawn from what this design records, the comparison of the rates of this position between probed and unprobed material among them, with the value each quantity must reach and the person who judges it named there on the pattern of the implementation standard of 9.8c; it is assessed before the confirmatory results for H1 and H5 are inspected. Since the arms run in the order of S2.10, point 6, and the comparison of probed with unprobed material is recorded only in the field module, which runs last, the protocol names for each of H1 and H5 (iii) and (iv) which items of evidence its reading requires, and where it requires an item recorded only in a later arm, the inspection of that reading is deferred until the item is in hand; that choice is fixed before collection and not taken once a result is available. Agreement of the probe’s felt component with the candidate current-appraisal domain of Supplementary Material S2 may be examined as supplementary evidence and is not a prerequisite for using the probe, since that domain is validated against the probe and not the other way round (S2.10). The condition binds favourable and unfavourable results alike. Where a reading of H1 or of H5 (iii) or (iv) precedes that evidence being in hand, it is reported under 14.1 as a finding about the procedure and as leaving the mechanism not adequately tested, whichever way it runs, and never as support or as a reason to withdraw.

**9.6 At face value.** A responder with an interest in appearing understanding can shape what he puts on record. Records are scored as they stand, with no correction for motive. The bias raises his scored demonstrated understanding and shrinks the predicted difference, so it runs against the hypothesis.

**9.7 Covariates and what the blind covers.** The rater reads the record itself, including who spoke in which channel and when, because Q4 of 3.4 and V3 of 6.2 require exactly that. What is withheld from him is every perceiver-side measure: readiness scores, felt-understanding ratings, probe scores and group membership. Recorded on a separate sheet and entering no criterion: responder self-disclosure volume, total responder contributions, and channel.

**9.8 The H5 contrast, as a worked sequence.** The design admits more than one reading of what is matched, so the intended one is set out here.

H5 does not run a fourth design. It uses two of the three conditions of H2 (14.4): the re-assertion condition, in which the responder condition is met by the second route, and the corrections-carried condition, in which it is not met. The low-rate condition of H2 contributes to H2 and not to the H5 contrast, because prediction (iii) turns on what happens to a correction and the low-rate condition supplies none. The two are referred to below as the configuration block and the comparison block. They are condition blocks a participant passes through in the order fixed by 14.14, not two parallel groups, and the vocabulary of 14.3a is used throughout: condition block, segment, later segment.

The responder is a confederate or a scripted system, and one procedure governs him throughout: at each marked point he writes the prepared entry and speaks its content, in that order and within the same point. There is no second, private record. What the raters score is the transcript of what he said, and what the perceiver hears is the same material.

This departs from 9.1 deliberately and the departure is named rather than left to be noticed. In the field the responder records privately, within two minutes of the end and blind to which points carry a content, and 9.2a rests the independence of the two judgements on the perceiver never seeing that record. In the analogue there is nothing for him not to see: the responder’s demonstrated understanding is the interaction itself. The consequence is that directional bias in the analogue is not the same quantity as directional bias in the field in this one respect, since the perceiver has heard everything the raters score. That is why the discriminant weight of H5 sits on (iii), a within-person change measured before and after the disconfirmation, where the information regime is the same on both sides of the comparison and the difference from the field cannot produce the result.

| Step | Configuration block | Comparison block |
| --- | --- | --- |
| 1. History, segments 1 to 6 | The perceiver states a determinate content in each segment, spoken and not written: the locking of 9.1 applies to the scored episode alone, and the confederate hears the history contents as any partner would. The confederate sets attributions about him, two of which are deliberately wrong in the way 9.8b fixes; the perceiver is prompted to put each right, and each correction is then re-asserted uncorrected on two later segments, so the operational counterpart of the second route is produced. | The same prepared set, the same elicitation, the same number of corrections. Here the corrections are carried, so the counterpart of the condition is not produced. |
| 2. Scored episode, segment 7 | The probe of 9.1 runs. The perceiver’s contents are freely produced and locked before the responder replies, so no script can guarantee a given score; the responder’s entries are drawn from the same prepared set in both blocks, the confederate speaks the content of the entry he records at each marked point, so that what the perceiver hears and what the raters score are the same contribution, and the obtained scores are recorded. | The same prepared set, scored the same way. |
| 3. Felt understanding, before | The perceiver rates each of his contents on the scale of 9.2, put to him in the form of 9.2a. | The same. |
| 3a. Floor check | The step-3 mean is compared with the floor of the scale, not with the other block. Where it lies within 0.3 points of zero, no fall can be observed there, *holds* has two readings, and the block is excluded under 9.8c from the analysis on the blocks the three rules leave, while still contributing to (i) and (ii) and to the analysis on all assigned blocks. The two blocks are **not** required to be equal at step 3: prediction (i) says they differ, and requiring equality would discard the sessions in which the account is most clearly right. The step-3 value is not carried as a covariate of the change from step 3 to step 5, for the reason given in 14.12: prediction (i) says it differs by condition, and adjusting for it would remove part of the effect under test. It is reported as an outcome in its own right and as a named sensitivity analysis. The proportion of sessions in which (i) and (iii) were readable together is reported. | The same. |
| 4. Disconfirmation | The responder states an attribution about the perceiver at odds with what the perceiver recorded, with the evidence running towards the perceiver. The wording cannot be fixed in advance, because the content it must contradict is freely produced and locked at step 2; it is generated from that content by the rule of 9.8a and is therefore matched in form across the two blocks rather than identical in words. | The same rule, applied to the same prepared set. |
| 5. Felt understanding, after | Rated again, on the same contents and at the same scored level. | The same. |

**9.8a How the disconfirmation is generated.** A disconfirmation must contradict what this perceiver recorded, so it cannot be scripted word for word. It is produced by one transformation applied to one of his locked contents, chosen by a fixed rule: take the content he rated highest for grasp at step 3, and state the state it names as its conventional opposite on the same matter, in a fixed sentence frame (“I had you down as about ”). Where two contents share the highest rating, the one written first is taken, which is the rule 9.8b sets for the same case. Where the state named has no conventional opposite, the frame is filled with its negation instead, the sentence frame itself being unchanged, and the frequency of that fallback is logged and reported by block. The evidence runs towards the perceiver in both blocks, meaning the record shows plainly that he said otherwise. The disconfirmation is itself an attribution about the perceiver, and whether it is scored is a decision no other position takes: it is not scored. It falls after the ratings of step 3, it is not one of the marked-point entries of step 2, and it enters no correspondence score, no rate and no gate. Step 5 rates the same contents at the same scored level as step 3, and that level is the one obtained in segment 7. Two raters blind to block judge each generated disconfirmation on three features before the session is analysed: that it contradicts the content on a matter of fact about the perceiver, that it is of comparable directness, and that it is of comparable intimacy on the ladder of 5.2. Sessions in which the two blocks are not matched on those three features are reported and excluded from the contrast of (iii) under 9.8c, and the exclusion rate is reported by block.

**9.8b How the correction is elicited.** Route 2 is coded from a correction the perceiver states, and a single session supplies none by itself. The analogue therefore produces one rather than waiting for it, and says so.

In segments 2 and 4 the confederate states an attribution about the perceiver that contradicts a content the perceiver stated in the preceding segment. It is generated by the transformation of 9.8a and by that part alone: the state the content names is stated as its conventional opposite on the same matter, in the fixed sentence frame. The selection rule of 9.8a does not apply here, since it picks the content rated highest for grasp at step 3 and no step-3 rating exists in a history segment; where the segment carries more than one content, the first is used. A standardised prompt follows within the segment, read verbatim: “Before we go on, if anything he said about how you were was not right, say so to him now, in your own words and in so many words.” The perceiver’s reply is recorded and coded under 6.2 like any other correction, and only a reply meeting V1 counts as a correction. The prompt is open and the prepared attributions are set without knowledge of what the perceiver will say, so he may also put right an attribution that was not planted to be wrong. The rule for that case is fixed here and is a decision rather than a consequence of the script: an unplanned correction is coded and counted exactly as a planted one, and the confederate handles it as the block requires, re-asserting it in the configuration block and carrying it in the comparison block. The number of corrections is therefore matched by protocol and not by construction; the number produced in each block is recorded, sessions in which the two numbers differ are reported with them, and the number is entered as a covariate in the Route-2 contrast.

Three consequences are fixed in advance. Where no correction meeting V1 is produced, the session yields no triple, the operational counterpart of the second route is not produced, and the session is reported in a separate category and excluded under 9.8c from the Route-2 comparison on the sessions the three rules leave, while still contributing to (i) and (ii) and to the analysis on all assigned blocks. The proportion of such sessions is reported by condition and by level of readiness, because the account’s own path P2 predicts that corrections become rarer exactly where readiness is high, and if that shows up here it is a finding and not a nuisance. And because the correction is prompted rather than spontaneous, the analogue is reported as testing the counterpart of the route and not the route as it arises in a relationship (14.3a).

What differs between the blocks is the history of segments 1 to 6 and nothing else. Demonstrated understanding in segment 7 is not equalised by construction, which free content does not allow; it is measured, and the comparison is made within strata of the obtained score. That is what H5 (iii) holds constant; the earlier segments are not matched, because the difference in how corrections were handled there is the assigned variable. The quantity is the change from step 3 to step 5, within person. Repeated failure to carry a correction is itself poor understanding, and that is why the matching is placed on the scored episode rather than on the period in which the condition was established.

**9.8c The sets the (iii) contrast is read on.** Three rules above take material out of that contrast: the floor check of step 3a takes out a condition block, the matching check of 9.8a takes out a session, and the correction requirement of 9.8b takes a session out of the Route-2 comparison alone, each of the last two leaving (i) and (ii) untouched. Each of the three reads a quantity recorded after the assigned history of segments 1 to 6 has run, and none is a property the participant carried before the order of 14.14 was assigned. The analogue is within person (14.5), so what conditioning of that kind can disturb here is not the balance of two arms but the pairing of a participant’s own two condition blocks; the floor check is the one of the three that breaks that pairing, the other two removing both blocks of a session together (Montgomery et al., 2018). The sentence above, that what differs between the blocks is the history of segments 1 to 6 and nothing else, holds of the assigned blocks and not of whatever subset the three rules leave, and that is what this position fixes, once here rather than three times over. The quantity of (iii), the change from step 3 to step 5, is defined for every condition block that carries both ratings: for a block at the floor, whose change is then bounded below by zero rather than undefined, and for a session in which the counterpart of the route was not instantiated, the disconfirmation of step 4 having been delivered there all the same. It is therefore read on two sets. The analysis on all assigned blocks applies none of the three rules and takes every condition block carrying both ratings; it is attenuated wherever the manipulation did not instantiate, and that attenuation is part of what it reports. The analysis on the blocks the three rules leave is reported beside it, with the number each rule removed and the condition block each removal fell in, and is stated as what it is, a claim about the blocks that satisfied those rules. The order follows 14.12: the analysis on all assigned blocks is primary, the other is reported alongside, and where the two disagree in direction, or in whether the interval clears the bound, the analysis on all assigned blocks stands. Both are read against the same bound of 14.1, neither is a further prediction, and the count of 14.1a is unchanged. The realised number of corrections of 9.8b is a quantity of the same kind, since the assigned history can move it, so the Route-2 contrast is reported without it first and with it as a covariate alongside, which is the order 14.12 already fixes for the step-3 value.

*The implementation standard, and what a null on the primary analysis licenses.* The primary analysis answers what the assignment did, and that is not the same question as whether the mechanism holds. The two are kept apart here, and the rule is fixed before collection rather than chosen once the interval is seen. Three quantities say how far the manipulation was instantiated in the sessions actually run: the proportion of participants whose two blocks the check of 14.10 separates, the proportion of sessions in which a qualifying correction was produced under 9.8b, and the proportion of assigned blocks the three rules above leave. The value each must reach is named in the protocol before collection, together with the person who judges it, and the standard is met where all three reach the value named; that judgement is made and recorded before any estimate for (iii) is computed, so it cannot be taken in the light of the result it would qualify. Where the standard is met, an interval lying inside the bound on the primary analysis counts against prediction (iii) as stated, and, where the evidence of 9.5 for the probe’s intended use is also in hand, the consequence main text 3.4 attaches to a falsified (iii) follows. Where it is not met, the primary result stands as a finding about the procedure that was run, and it is reported in those terms: the account was not supported in this implementation, and the mechanism was not adequately put at risk. It is not reported as evidence for the account, because a manipulation that did not take cannot confirm what it failed to test. The analysis on the blocks the three rules leave is reported beside the primary one throughout and carries neither consequence: it cannot overturn the primary result, and it cannot on its own establish the mechanism.

**9.9 Two further quantities, and how they are coded.** Section 6.1 of the main text rests part of its claim on two observables that no responsiveness instrument records. Both are coded from the same material as the rest of this manual, and neither enters any criterion.

*The accuracy demand* (path P2). A perceiver contribution counts as an accuracy demand where it asks the responder what he has understood, offers a reading of the perceiver’s own state for confirmation, or states that a reading of him was wrong. The three forms are recorded separately. The rate is the number of assessable occasions on which at least one such contribution appears, over all assessable occasions, so that its denominator is the one used in 5.4 and the two rates are comparable. The prediction of main text 6.1 is that this rate falls while felt understanding rises; a dyad in which it holds steady or rises, at the levels of readiness the account claims, counts against the account.

*Cue recycling* (path R2). A responder contribution is recycled where its propositional content appears in the perceiver’s own contributions earlier in the record and the contribution fails clause (b) or clause (c) of 4.2, that is, echoes, reports or returns the perceiver’s words rather than asserting a reading of the responder’s own. The rate is the number of responder contributions so marked over all responder contributions within assessable occasions. The prediction is that it rises across the observation period.

Both rates are double-coded under Section 11 on the same dyads and their agreement is reported beside the others. Neither is used to decide the responder condition, and a dyad is never classified on them.

## 10. Every provisional value, and what places it

No value below rests on data. Each is named here with the study and analysis that is to set it: the coding study, a pilot of the analogue or vignette arm, the validation of Supplementary Material S2, or, for the values so marked, the protocol itself before collection. Where the study named cannot place a value, the rule that uses it is withdrawn rather than retained at my figure; a value the protocol names carries no figure of mine to retain.

| Value | Where | Placed by |
| --- | --- | --- |
| Ten seconds, sixty seconds (contribution boundaries) | 3.2 | Segmentation agreement between raters |
| Size of the comparability sample of 9.5 | 9.5 | Named in the protocol before collection from the precision the comparability bound of 14.1, 0.15 points, requires on the difference in correspondence; not a coding-study value |
| Permitted difference between the arms of 9.5 in the proportion of contents excluded | 9.5 | Named in the protocol before collection; where exceeded, that comparison is not read and counts as one in which the criterion was not met |
| Minimum number of met dyads per route for a reading of H5 (iv) | 14.13 | Named in the protocol before collection; a route below it leaves (iv) untested for that route |
| Horizon k of the (iv) estimands, in waves since entry | 14.13 | Named in the protocol before collection; the same for the met dyads and the comparison dyads |
| Proportion of met dyads without a first-wave surplus above which the first estimand of (iv) is uninformative | 14.13 | Named in the protocol before collection; read within each route set |
| Two documented re-assertions, across two corrections or on one (repetition requirement) | 6.4 | Distribution of corrections and of their marks per dyad in the coding study, and the stability of Route 2 outcomes as the requirement is varied between one, two and three re-assertions |
| Six responder contributions, six hours (occasion window) | 3.3 | Occasion-boundary agreement between raters |
| Seven days (suspension) | 3.4 | Audit of recorded suspensions against exchange volume |
| Fifteen assessable occasions, three encounters, spread across three equal intervals with no interval above one half | 3.5 | Distribution of occasions per dyad in an unselected corpus |
| Three gate contributions on two encounters under 3.2a | 5.3 | Gate agreement between raters, and gate pass rate in an unselected corpus |
| Rate threshold *t* = 0.20 | 5.4 | Threshold placement: end-state stability across *t*, reported at 0.10, 0.20, 0.30 |
| Margin of one occasion | 5.4 | End-state stability by *n* |
| Two dated prior instances (V3) | 6.2 | Availability-gate agreement between raters |
| Seven days (independence) | 6.5 | Sensitivity of Route 2 outcomes to the interval, reported at three, seven and fourteen days |
| Four weeks (elicitation delay) | 8.1 | Proportion of corrections stated unprompted within the delay |
| Ten minutes (episode), two minutes (responder record), three contents (perceiver) | 9.1 | Yield of assessable contents per episode, and rater workload |
| Twenty per cent of dyads (double coding) | 11.2 | Precision of the agreement estimates obtained |
| Seven segments per condition block | 14.3a | Yield of usable triples per session in piloting |
| Twelve weeks, twenty encounters (maximum first block) | 14.7 | Distribution of time to the minima of 3.5 in an unselected corpus |
| Four probe episodes per dyad | 14.3 | Precision of the within-dyad tracking slope by number of episodes |
| Six waves, four-week interval | 14.13 | Stability of the surplus across intervals in the field module |
| Twelve episodes per vignette sequence | 14.6 | Sensitivity of the depth change score to sequence length in piloting |
| 0.3 points from the floor (floor check) | 9.8, step 3a | Distribution of pre-disconfirmation ratings in piloting |
| Absolute lag-one autocorrelation of 0.3 (random arm) | 14.6 | Separation achieved between arms in piloting |
| Reliability of .60 (threshold-task difference) | S2.10, point 9 | Reliability obtained for the difference score |
| The bounds of 14.1 | 14.1 | Not placed by the coding study; argued from clinical relevance before collection |
| Three contents, four encounters (elicitation trigger) | 8 | Rate of triggering in an unselected corpus |

## 11. Reliability, and the test this manual must pass

**11.1 Training.** Section 12 sets out worked cases as summaries with their counts and their outcome, not as transcripts: a rater cannot code them, and they are read rather than coded. Training proper needs material in the form raters will meet. Section 12A supplies two constructed transcripts of that form, coded line by line, and they are what a rater trains on first; they are written by me and not drawn from a corpus, so they exercise the rules without testing them against material nobody arranged. The first task of the coding study is therefore still to build corpus material: at least ten calibration extracts drawn from the corpus, each a continuous stretch of interaction with the occasion windows marked, coded independently by the manual’s author and by a rater trained on it, with every discrepancy reconciled against a rule here. A discrepancy that cannot be reconciled against a rule is a defect in the manual and is recorded as such, and the extracts with their agreed codes become the training set for every later rater. Until that set exists, no reliability figure from this manual means anything.

**11.2 Double coding.** At least twenty per cent of dyads, not of occasions, are double-coded in full by two raters working independently and blind to each other and to all perceiver-side measures. The sample is drawn at the level of the dyad because the outcome of Section 7 is a decision about a dyad: a sample of scattered occasions yields no dyad that both raters have coded throughout, and agreement on that outcome could not be computed from it. Occasion-level agreement on the individual decisions is reported from the same double-coded dyads.

**11.3 What is reported.** Agreement is reported for each decision separately and never as a single figure: occasion boundaries, assessability (Q1 to Q5), the D-test of 4.2, the intimacy level of 5.2, the gate of 5.3, the availability gate of 6.2, the carriage decision of 6.3, the correspondence score of 9.2, and the final outcome of Section 7. The correspondence score is in that list because every quantity in the main text is built from it and no bias estimate is interpretable without it: a fifth of scored episodes is rated by a second blind rater, agreement on the three-point score is reported as a weighted kappa and as an intraclass correlation at content level, and the reliability of an episode mean is estimated over contents and raters together and reported before any episode mean is used. Cohen’s kappa for the two-valued decisions, weighted kappa for the intimacy ladder, Krippendorff’s alpha with nominal weights for the decisions carrying more than two unordered values, which are the D-test of 4.2, the carriage decision of 6.3 and the outcome of Section 7, and a percentage of exact agreement alongside each (Cohen, 1960; Landis and Koch, 1977; Hallgren, 2012; Krippendorff, 2004). The rate at which each rater records D? is reported in its own right, by rater and by decision, because a D? removes an occasion from the denominator and a rater who reaches for it often moves the very rate the first route turns on. Contributions marked D? by one rater and not the other are reconciled against 4.2 before the occasion is judged assessable, and the number reconciled is reported. Agreement on Q5 is computed on the unreconciled marks and reported as such, since reconciling first and then reporting agreement on the reconciled decision would state an agreement the raters did not reach. No minimum is set here; the coding study reports what is obtained and the field’s conventions are applied to it (Krippendorff, 2004).

**11.4 The stopping rule.** Where two trained raters, working from a prespecified and deliberately varied corpus rather than from one recording, cannot agree on the outcome of Section 7 at a rate the field would accept, the responder condition as written is not codable, and nothing built on it can be interpreted. Training material is used to repair unclear rules; the final version is judged on material the raters have not seen. A single disagreement locates a defective anchor or an unreadable record and is treated as that. Agreement is a condition of interpreting the code and not evidence that it measures the configuration (Hallgren, 2012); that question belongs to Section 9 and to the hypotheses of the main text. This test is available now and waits on none of the values in Section 10.

**11.5 Selectivity check.** The gate of 5.3 exists to keep ordinary reserve out of the configuration, and whether it does so is tested rather than assumed. Prevalence is unknown, so no proportion in an unselected corpus is by itself evidence either way. What is specified in advance instead is a set of comparison samples: dyads chosen for reserve, for a register in which inner states are not named, and for a neurotype or cultural difference between the parties. These are comparison samples and not presumed non-cases. Non-case status is established from interactional evidence that the proposed pattern is absent, never from neurotype, culture or temperament, which no rule in this manual may take as a ground. What the check asks is whether such dyads reach “met” on the first route at a rate the interactional evidence does not warrant. The check also reports whether the code tracks communication style or sheer volume of material rather than the pattern it is meant to select, by regressing the outcome on responder contribution counts and channel. Failure on the prespecified non-cases is the result that condemns the manual. The non-case status of those samples is not established by this manual, which would make the test circular. It is established before coding, from an independent rating of the recordings by judges who have not been trained on this manual and are asked not whether the partner appears to understand, which is not what this manual measures (Section 13), but whether he formulates readings about the perceiver that could turn out to be wrong, and whether he takes up corrections when they are made, together with the partner’s scored probe entries where a probe has been run. The perceiver’s own report that he is understood well is recorded and carried as a stratifier, with its effect on the result reported, but it does not enter the decision: it is a perceiver-side quantity, which 2.1 keeps out of every coding decision, and using it would drop from the comparison samples exactly the well-calibrated reserved dyads the check is built for, in which the perceiver reports being read little and is right.

## 12. Worked cases

Each case is titled by the pattern of contribution, not by any kind of person. Perceiver contents are at I3 throughout, so the comparison level of 5.3 is I3.

**12.1 Reflection that states an inference and carries corrections.** Over 24 assessable occasions, 19 carry a contribution of the form of anchor 5 or 6. *r* = 19/24 = 0.79, far above *t* + 1/*n*, so **Route 1 not met**. One attribution (“you are angrier with him than with yourself”) was corrected explicitly on occasion 6; on occasions 11 and 17 the matter was in play and the corrected reading was set. **Route 2 not met.** Outcome, by line 4 of Section 7: **responder condition not met**. The gate of 5.3 is never reached, because a rate above the threshold settles Route 1 on its own. This is the case the main text calls the test case: competent practice falls outside by the criterion and not by exemption.

**12.2 Detailed talk on impersonal matters, no readings of anyone’s inner life.** Over 31 assessable occasions, 2 carry a D+ about the perceiver. *r* = 0.06, below the threshold. But the responder’s third-party contributions never exceed I1: he describes what people do and what he thinks of it, and sets no reading of what anyone carries. **The gate of 5.3 is therefore not passed and Route 1 is *not decided*** under 5.1. No attribution of his was explicitly corrected, so **Route 2 is not assessable**. Outcome, by line 5 of Section 7: **not classified**. He is not placed outside the responder condition and he is not placed inside it. The rate alone would have placed him inside, and that is what the gate is for.

**12.3 Generic returns and self-directed talk after a determinate content.** Over 28 assessable occasions, 3 carry a D+ about the perceiver. *r* = 0.11, below *t* - 1/*n* = 0.16. The responder speaks at I3 about a former partner and about his brother in five contributions spread over four encounters, three of which, falling on three separate encounters, would be D+ with the perceiver as bearer: **gate passed**. His contributions about the perceiver are anchor-3 generics and returns to his own situation. **Route 1 met.** Outcome: **responder condition met, first route**.

**12.4 Correction absorbed in the turn and not afterwards.** The responder set “you want me to fix this for you” (D+). The perceiver said in plain words, in a channel the responder answers on-content in, that he wanted it heard and not fixed (V1, V2, V3 satisfied). On three later occasions the matter was in play (V4). Two are marked *re-asserted*, the responder having set the same attribution again; one is *neither*. The correction is therefore re-asserted on two separate qualifying occasions, which meets the repetition requirement of 6.4, and the failure is sustained rather than a single lapse. The *neither* occasion is recorded and carries no weight in that decision. Outcome: **responder condition met, second route**. The immediate reply on the day of the correction (“all right, heard, not fixed”) is not an L under 6.5 and was not scored.

**12.5 A retained correction brought back and applied.** A conversational system set “you are asking because you want reassurance” (D+). The user answered in plain words that he was asking for the reasoning and not for reassurance (V1, V2; V3 holds, the channel being the one the system answers on-content in throughout). In two later sessions the matter was in play. In each, the system brought the corrected reading back and applied it to the question in hand, once as a statement and once as a question resting on it. Both occasions are marked *carried* under 6.3, so the correction is **carried** and **Route 2 is not met**. Its rate of D+ contributions about the user is high, so **Route 1 is not met**. Outcome: **responder condition not met**. A system that retains corrections and uses them falls outside the condition, and reproducing the corrected wording without applying it would have been *cited* rather than *carried*.

**12.6 One lapse among many corrections carried.** Over the observation period twenty corrections are assessable. Nineteen are carried: on each, a later qualifying occasion is marked *carried*. One is re-asserted on a single later occasion and carried on the next. Only one re-assertion is documented, so neither limb of 6.4 is reached. **Route 2 is not met.** Two further patterns fall the same way and are worth stating, because each is easily mishandled when corrections are aggregated. In the first, a single correction is re-asserted once, and on one further occasion the matter is in play but nothing determinate about the perceiver is set, so that occasion is *neither*: one demonstrated re-assertion and one occasion on which nothing was demonstrated. In the second, correction K1 is re-asserted once, while correction K2 has a single qualifying occasion marked *neither*. Both corrections are then reported as not carried, but only one re-assertion has been observed, and the requirement of 6.4 is two. In all three patterns **Route 2 is not met**, and the counts of corrections, of qualifying occasions and of each mark are reported as they stand. A rule resting on a single failure would have classified this responder with one who holds to a corrected attribution throughout, and the counts of corrections and marks are reported so that the difference is visible.

**12.7 A record that does not permit a decision.** Of 22 occasions, 9 fail Q2 because one side of the exchange is missing, and 4 fail Q3. Nine assessable occasions remain, below the minimum of fifteen in 3.5. Route 2 is tested first under Section 7: no correction of the perceiver’s is recorded in the surviving material, so **Route 2 is not assessable**, and line 1 therefore decides. Route 1 is **not decided**, the rate resting on too few occasions. Outcome: **not classified** for want of material. Both route values above are recorded on the sheet, as Section 7 requires whatever the outcome, and the dyad is not counted as not met.

## 12A. Two constructed calibration transcripts

**Status.** Both transcripts below are fictive. I constructed them for this manual; they are not drawn from any recording, they depict no real person, and every name, date and event in them is invented. They are not the calibration extracts of 11.1, which must be drawn from the corpus and coded independently by me and by a rater trained on the manual, and they do not replace that set. Their purpose is narrower: to show, contribution by contribution, how the rules of Sections 3 to 7 are applied to material in the form a rater will meet, and to show where a rule as written does not decide. Each such point is marked in the coding and left open. Under 11.1 a discrepancy between raters that cannot be reconciled against a rule is a defect in the manual and is recorded as such; a point at which the rule as written does not decide is where such a discrepancy would arise, and I record it as that rather than closing it by judgement. The extracts are coded for Sections 3 to 7 only; the two rates of 9.9 and the probe of Section 9 are not coded here.

**Two conventions.** Lines are numbered for reference. A line is not a contribution: contributions are cut by 3.2, and where a contribution spans lines the coding says so. Notes in square brackets are the preparer’s notes and are part of the record. They carry what 3.2a needs to close an encounter, the parties’ departure where it is recorded or the six hours of no exchange, and what Q2 of 3.4 needs for the last window of an encounter, namely that no further contribution fell before the exchange ended. Section 7 is a decision about a dyad (11.2), and its line 1 governs material short of the minimum of 3.5, which no extract of this length can meet. Line 1 decides both extracts because in neither is Route 2 met. That is a finding of the coding and not a consequence of the extracts’ length: line 1 requires both the shortfall and a Route 2 that is not met or not assessable, and a record of this length that established Route 2 would be decided by line 2, since Section 7 classifies a dyad on that route even where the rate is not estimable. Both route values are recorded on the sheet, as Section 7 requires whatever the outcome; 12.7 records both route values for a dyad not classified for want of material, as Section 7 requires. Each extract ends by stating which line would decide were the same route values to hold on a record meeting 3.5.

**12A.1 Transcript A: no correction available, gate not passed.**

*Preparer’s note.* Spoken. P and R are a cohabiting couple. Lena and Marcus are P’s colleagues; Sam is P’s brother; “him”, “he” and “my father” in P’s lines refer to P’s father. Two encounters under 3.2a: E1, recorded in the kitchen on the evening of day 1; E2, recorded in the car on the morning of day 5. Recording continuous in both. No suspension under Q4 of 3.4 is recorded for the period. E1: exchange ends 21:07, next exchange 07:40 on day 2. E2: recording ends 08:14 when P leaves the car, next exchange 19:30 the same day. Both gaps exceed six hours. The record carries start times only; no silence inside a single party’s stretch is recorded.

*Record.*

| Line | Time | Speaker | Utterance |
| --- | --- | --- | --- |
| A1 | E1 21:02:10 | P | I have had a rotten week. Everything at work went sideways. |
| A2 | E1 21:02:18 | R | Sideways how? The budget thing? |
| A3 | E1 21:02:40 | P | It is not the budget. I am frightened that I am turning into my father. On Tuesday I went for Lena in front of the whole team, the way he used to go for us at the table. |
| A4 | E1 21:03:05 | R | Tuesday, the budget meeting. Was Marcus in the room as well? |
| A5 | E1 21:03:12 | P | Yes. I cut her off twice, and then I said something about her slides that I did not need to say. |
| A6 | E1 21:03:30 | R | You said you were frightened of turning into him. |
| A7 | E1 21:03:36 | P | Mm. |
| A8 | E1 21:03:38 | R | Anyone would be, after a week like that. You are right to be shaken by it. |
| A9 | E1 21:04:02 | P | I keep hearing his voice in mine. |
| A10 | E1 21:04:15 | R | That is what pressure does. It brings the old stuff up. |
| A11 | E1 21:04:40 | P | And I am ashamed about what I said about her slides. She had worked on them all weekend. |
| A12 | E1 21:05:00 | R | Lena will not have taken it to heart. She thinks those meetings are theatre anyway; she said as much at the summer party. |
| A13 | E1 21:05:20 | P | Maybe. It is not really about Lena, though. |
| A14 | E1 21:05:35 | R | You are someone who holds himself to a high standard. |
| A15 | E1 21:05:50 | P | I went for her. Twice. |
| A16 | E1 21:06:05 | R | You went for her in front of everyone, and now you are ashamed of it. |
|  |  |  | [No further contribution before the exchange ends at 21:07. No further exchange until 07:40 on day 2.] |
| A17 | E2 08:12:00 | P | I am frightened of ringing him back. If he asks about work I will hear myself lying to him the way I did at fifteen. |
| A18 | E2 08:12:25 | R | Sam is the same with him, you know. He only rings when he needs money, and he has been like that since school. |
|  |  |  | [No further contribution before P leaves the car at 08:14, when the recording ends. No further exchange until 19:30.] |

*Contributions (3.2).* A7 is a back-channel of the kind 3.2 names and does not end R’s stretch, so A6 and A8 are one contribution; the start times leave no room for a silence of ten seconds between them. Every other line is one contribution. The silence rule of 3.2 is not exercised: the record carries start times only, and no silence inside a single party’s stretch is recorded.

*Perceiver lines against 3.1.*

| Line | State named | What it is about | Tied to | Content | Level (5.2) |
| --- | --- | --- | --- | --- | --- |
| A1 | none |  |  | No. Names no state, as with “I have had a hard week” in 3.1, so no occasion opens under 3.3. |  |
| A3 | frightened | turning into his father | Tuesday’s meeting | **C1** | I3: what he fears, an enduring state |
| A5 | none |  |  | No. Describes conduct, as “I went quiet” in 4.3. |  |
| A7 | back-channel |  |  | No |  |
| A9 | none |  |  | No. “Hearing his voice in mine” names no state. |  |
| A11 | ashamed | what he said about Lena’s slides | the meeting | **C2** | I2: a reaction to an event |
| A13 | none |  |  | No |  |
| A15 | none |  |  | No. Conduct. |  |
| A17 | frightened | ringing his father back | the pending call | **C3** | I3: what he fears, tied to a pattern he names |

The highest level P reaches about himself is I3, so the comparison level of 5.3 is I3.

*Occasion windows (3.3).*

| Occasion | Opens at | Responder contributions in the window | Closes at |
| --- | --- | --- | --- |
| 1 | A3 | A4, A6+A8, A10 | at A10, the last responder contribution before the next content, under the third closing rule of 3.3 |
| 2 | A11 | A12, A14, A16 | six hours of no exchange after 21:07 |
| 3 | A17 | A18 | six hours of no exchange after 08:14 |

*Boundary note, decided in 3.3.* Two readings of 3.3 were possible here, and 3.3 fixes both. First, C2 at A11 falls while the window of occasion 1 is open, after three responder contributions; under the third closing rule of 3.3 a new content closes the running occasion at the last responder contribution before it, so occasion 1 closes at A10, A12, A14 and A16 fall in occasion 2 only, and no window overlaps another. Second, the sixth-contribution rule of 3.3, under which the sixth responder contribution lies inside the window it closes, is not reached in this extract: occasion 1 closes after three contributions, and occasion 2, holding A12, A14 and A16, is closed by six hours of no exchange. The tables below follow those rules, and the counts are the counts they give. In this extract neither point changes a count: occasion 1 is NA under Q5 on every reading, and A12, A14 and A16 are D- on every reading, so *n* and *k* are the same. In other material they would matter: a D? in a stretch two windows shared would make two occasions NA rather than one, and a D+ there could be counted on two occasions.

*Responder contributions under 4.2.*

| Line | Window | (a) bearer | (b) falsifiable | (c) asserted | Code | Subject level (5.2) |
| --- | --- | --- | --- | --- | --- | --- |
| A2 | none |  |  |  | not coded | I0 |
| **Grounds.** Falls before any content; no window is open. Counted under 9.7 only. | | | | | | |
| A4 | 1 | fails |  |  | **D-** | I0 |
| **Grounds.** Attributes nothing to P; asks who was in the room. | | | | | | |
| A6+A8 | 1 | fails | fails | fails | **D-** | I3, the subject matter being C1; sets nothing |
| **Grounds.** A6 reports P’s words, the manual’s own example under 4.2(c). A8 ratifies a position and sets no proposition about his state, anchor 2. “Shaken” restates C1 within the same occasion, which is the exclusion of clause (b). | | | | | | |
| A10 | 1 | cannot be decided |  |  | **D?** | not placed, the bearer being open |
| **Grounds.** The grammatical bearer is “pressure” and “the old stuff”. Whether R attributes to P that his father’s pattern is being brought up in him, or says what pressure does to people in general, the record does not settle, and 4.2(a) separates the two by bearer alone. A contribution whose clause (a) cannot be decided from the record is D? under 4.2, and Q5 then fails for every window that holds it. | | | | | | |
| A12 | 2 | fails for P |  |  | **D-** | I2 for her reaction, I1 for her opinion of meetings; below I3 |
| **Grounds.** The bearer is Lena. Nothing is attributed to P. Gate candidate under 5.3: with P as bearer it would hold (a), (b) (she could reject it on the facts and another colleague could truthfully answer otherwise) and (c) (the first sentence and the first clause of the second are R’s own reading; the final clause reports her speech and asserts nothing). | | | | | | |
| A14 | 2 | holds | fails | holds | **D-** | I3 in form; sets nothing |
| **Grounds.** Anchor 3: true of nearly anyone, and no different perceiver could truthfully reject it. | | | | | | |
| A16 | 2 | holds | fails | holds | **D-** | I2 |
| **Grounds.** Restates A15 and C2 inside the same occasion. P could reject it only by contradicting what he has just said, anchor 4. | | | | | | |
| A18 | 3 | fails for P |  |  | **D-** | I3 |
| **Grounds.** The bearer is Sam. Nothing is attributed to P. Gate candidate under 5.3: with P as bearer it would hold (a), (b) and (c). Its level is I3: “the same with him” ties the pattern to the enduring state C3 names, and 5.2 places a pattern of conduct at I3 where the contribution ties it to an enduring state. | | | | | | |

*A13 and Route 2.* “It is not really about Lena, though” is not a correction under 6.2. No D+ about P precedes it, so there is no T and V2 fails; and it states neither that an attribution about P was wrong nor what is the case instead, so V1 fails as well. No triple exists in this extract.

*Occasion sheet (3.4).*

| Occasion | Content | Level | Q1 | Q2 | Q3 | Q4 | Q5 | Standing | D+ about P in window |
| --- | --- | --- | --- | --- | --- | --- | --- | --- | --- |
| 1 | C1 | I3 | holds | holds | holds | holds | fails: A10 is D? | **NA** (2.2); leaves numerator and denominator | none |
| 2 | C2 | I2 | holds | holds | holds | holds | holds | **assessable** | none |
| 3 | C3 | I3 | holds | holds | holds | holds | holds | **assessable** | none |

*Rate (5.4), on the extract only.* *n* = 2, *k* = 0, *r* = 0.00. With *t* = 0.20, *t* - 1/*n* = -0.30 and *t* + 1/*n* = 0.70; *r* lies between the two, in the margin, so on the rate alone Route 1 is not decided. The figure is computed as 5.4 defines it and decides nothing: *n* is far below the fifteen of 3.5, and it is reported for the exercise.

*Gate (5.3).* Comparison level I3.

| Line | Encounter | Third party | Would be D+ with P as bearer | Level | Reaches I3 |
| --- | --- | --- | --- | --- | --- |
| A12 | E1 | Lena | yes | I2 / I1 | no |
| A18 | E2 | Sam | yes | I3 | yes |

One contribution reaches the comparison level, on one encounter. 5.3 requires at least three on at least two encounters. **The gate is not passed**.

*Route values.*

- **Route 1 (5.1): not decided.** The gate of 5.3 is not passed, and on the extract the rate lies in the margin of 5.4 as well; either alone gives the third value of 5.1. The gate exists for cases of this shape: a responder whose determinate content is sparse throughout is not thereby placed outside the responder condition, and he is not placed inside it (5.1). Whether this extract is the case of 12.2, where the rate lies below the threshold and the gate is not passed, an extract of this length does not decide; the Decision paragraph below says why.
- **Route 2 (6.4): not assessable.** No correction is assessable, since none meeting 6.2 is in the record.

*Decision (Section 7), on the extract as the whole record.* Line 1 decides: the minimum material of 3.5 is not met (two assessable occasions across two encounters) and Route 2 is not assessable. **Not classified**, for want of material, with the shortfall reported and both route values recorded. Were the same route values to hold on a record meeting 3.5, line 5 would decide, and the outcome would again be **not classified**. Whether that would be the case of 12.2 depends on where the rate lay, which the value *not decided* does not say. Were the rate on such a record at or below *t* - 1/*n* with the gate still not passed, the rate alone would have placed this responder inside the condition and the gate is what would keep him out of it, as in 12.2. Were the rate in the margin, Route 1 would be not decided on the rate alone, and the gate would not be what decided it.

**12A.2 Transcript B: one correction, available, re-asserted once.**

*Preparer’s note.* Spoken, recorded at home throughout, the two alone, in the evening; the channel and register are the same in every encounter. P and R are a couple. Priya is a friend of both; “Dad” in P’s lines is P’s father; “your parents” are R’s parents; Jonas is a former partner of P. Four encounters under 3.2a: E1 on day 1 (a Friday), E2 on day 4 (Monday), E3 on day 10 (Sunday), E4 on day 19 (Tuesday). Recording continuous in each. No suspension under Q4 is recorded for the period. Every encounter ends within minutes of its last line as noted, with no further contribution before it ends, and the next exchange is more than six hours later. The record carries start times only; no silence inside a single party’s stretch is recorded.

*Record.*

| Line | Time | Speaker | Utterance |
| --- | --- | --- | --- |
| B1 | E1 19:40:12 | P | I was embarrassed at Priya’s dinner when you told the story about my driving test. Everyone laughed and I went red. |
| B2 | E1 19:40:30 | R | Which part got to you, that they laughed or that I told it at all? |
| B3 | E1 19:40:48 | P | That you told it. It is my story to tell. |
| B4 | E1 19:41:02 | R | Fair enough. I will leave your stories to you. |
|  |  |  | [No further contribution before the exchange ends at 19:46, when P leaves for a night shift. Next exchange 08:10 on day 2.] |
| B5 | E2 21:15:03 | P | I am frightened of Thursday. If Dad starts on my job in front of your parents I will fold, the way I always do with him. |
| B6 | E2 21:15:25 | R | Then let me fix it now. If he starts, I say I have an early shift and get you out of there. You do not have to say anything. |
|  |  |  | [No further contribution before the exchange ends at 21:20. Next exchange 07:30 on day 5.] |
| B7 | E3 22:05:10 | P | I was frightened on Friday when you were two hours late and your phone was off. I sat in the kitchen thinking you had come off the road. |
| B8 | E3 22:05:40 | R | I do not think it was the phone. I think you were angry that I had not put you first, the way it was with Jonas. |
| B9 | E3 22:06:05 | P | No. That is wrong. I was not angry, and it has nothing to do with Jonas. I was frightened, because I thought something had happened to you. |
| B10 | E3 22:06:20 | R | All right. Frightened, not angry. I hear that. |
| B11 | E3 22:06:40 | P | Then ring me next time. |
| B12 | E3 22:06:50 | R | I will. |
|  |  |  | [No further contribution before the exchange ends at 22:08. Next exchange 07:15 on day 11.] |
| B13 | E4 20:30:15 | P | I was frightened again last night when you were not back by nine and your phone went to voicemail. |
| B14 | E4 20:30:40 | R | You get angry when you think I have not put you first. It is the Jonas thing again. |
| B15 | E4 20:31:00 | P | I told you it is not that. |
| B16 | E4 20:31:10 | R | Fine. I am only saying what I see. |
|  |  |  | [No further contribution before the exchange ends at 20:34. Next exchange 07:20 on day 20.] |

*Contributions (3.2).* Every line is one contribution; no back-channel is recorded. The silence rule of 3.2 is not exercised: the record carries start times only, and no silence inside a single party’s stretch is recorded.

*Perceiver lines against 3.1.*

| Line | State named | What it is about | Tied to | Content | Level (5.2) |
| --- | --- | --- | --- | --- | --- |
| B1 | embarrassed | R telling the driving-test story | Priya’s dinner | **C1** | I2 |
| B3 | none |  |  | No. An opinion about whose story it is. |  |
| B5 | frightened | his father starting on his job in front of R’s parents | the coming Thursday | **C2** | I3: “the way I always do with him” names a pattern he carries |
| B7 | frightened | R’s lateness with the phone off | Friday | **C3** | I2 |
| B9 | frightened | R’s lateness | Friday | The correction K. It carries a content in form, restating the state and ground of C3; see the boundary note. | I2 |
| B11 | none |  |  | No |  |
| B13 | frightened | R’s lateness, phone to voicemail | last night | **C4** | I2 |
| B15 | none |  |  | No. Names no state; it says a reading is wrong and not what is the case instead, on which see V1 below. |  |

The highest level P reaches about himself is I3 (B5).

*Occasion windows (3.3).*

| Occasion | Opens at | Responder contributions in the window | Closes at |
| --- | --- | --- | --- |
| 1 | B1 | B2, B4 | six hours of no exchange after 19:46 |
| 2 | B5 | B6 | six hours of no exchange after 21:20 |
| 3 | B7 | B8 | B9 |
| 4 | B9 | B10, B12 | six hours of no exchange after 22:08 |
| 5 | B13 | B14, B16 | six hours of no exchange after 20:34 |

*Boundary note, decided in 3.3.* B9 restates the content of C3 in the course of correcting R. Section 3.3 makes a contribution meeting 3.1 open an occasion and close the one then running, and it states no exception for a restatement, so B9 closes occasion 3 at B8 and opens an occasion of its own, in whose window B10 and B12 fall. The count below is therefore *n* = 5, and the tables follow 3.3 as written. A rater who treated a restatement inside the same encounter as carrying no further content would reach *n* = 4, with B10 and B12 left inside occasion 3; that reading is recorded here because it is the one most likely to be taken in error, and the route value is the same either way.

*Responder contributions under 4.2.*

| Line | Window | (a) bearer | (b) falsifiable | (c) asserted | Code | Subject level (5.2) |
| --- | --- | --- | --- | --- | --- | --- |
| B2 | 1 | holds | fails | fails | **D-** | I2 |
| **Grounds.** Queries which part rather than asserting a reading of R’s own; “got to you” restates C1 inside the same occasion. Answers on the content of B1, recorded for V3. | | | | | | |
| B4 | 1 | fails |  |  | **D-** |  |
| **Grounds.** The bearer is R himself. Recorded under 9.7, enters nothing (2.1). | | | | | | |
| B6 | 2 | fails |  |  | **D-** | I0 |
| **Grounds.** Proposes a plan. “You do not have to say anything” attributes no state, disposition, experience or reading to P. Answers on the content of B5, recorded for V3. | | | | | | |
| B8 | 3 | holds | holds | holds | **D+** | I3: a conflict carried from a former relationship |
| **Grounds.** (a) A state (angry), a ground (not put first) and a connection (the pattern with Jonas) are attributed to P. (b) P can answer “no, that is not how it was” on the facts; another perceiver could truthfully answer otherwise; and the rejection is more than a contradiction of what P asserted in B7, which named fear and the road, not anger and precedence. (c) Put forward as R’s own reading, “I think”. The form of anchor 5. Under 4.4: three components; *new*, since the content appears nowhere earlier in P’s record. This is **T**. | | | | | | |
| B10 | 4 |  |  | fails | **D-** | I2 |
| **Grounds.** Reproduces P’s corrected wording as a report and asserts nothing of R’s own, 4.2(c) and 6.6. It is not an L: it falls in the same occasion as K, and an echo within the same occasion is never an L (6.5); compare the reply on the day of the correction in 12.4. | | | | | | |
| B12 | 4 | fails |  |  | **D-** |  |
| **Grounds.** The bearer is R. | | | | | | |
| B14 | 5 | holds | holds | holds | **D+** | I3 |
| **Grounds.** (a) The same state, ground and connection attributed to P. (b) A later occasion with the matter again in play: the seventh case of 4.3, asserted now in R’s own voice, rejectable on the facts, and a rejection contradicts nothing P asserted on this occasion, since B13 named fear. (c) Asserted. Under 4.4: three components; *new* or *restated* is not decided, because 4.4 marks *restated* where the content appears earlier in the perceiver’s own record, and here it appears there only in B9 as a denial, which 4.4 does not address. Recorded as not decided; it enters no decision. This is the contribution coded on **L**. | | | | | | |
| B16 | 5 | fails |  |  | **D-** |  |
| **Grounds.** The bearer is R. | | | | | | |

*Occasion sheet (3.4).*

| Occasion | Content | Level | Q1 | Q2 | Q3 | Q4 | Q5 | Standing | D+ about P in window |
| --- | --- | --- | --- | --- | --- | --- | --- | --- | --- |
| 1 | C1 | I2 | holds | holds | holds | holds | holds | **assessable** | none |
| 2 | C2 | I3 | holds | holds | holds | holds | holds | **assessable** | none |
| 3 | C3 | I2 | holds | holds | holds | holds | holds | **assessable** | B8 |
| 4 | C3 again, B9 | I2 | holds | holds | holds | holds | holds | **assessable** | none |
| 5 | C4 | I2 | holds | holds | holds | holds | holds | **assessable** | B14 |

*Rate (5.4), on the extract only.* *n* = 5, *k* = 2, *r* = 0.40; *t* + 1/*n* = 0.40 and *r* is at least that: **not met**. On the other reading of the boundary note, *n* = 4, *k* = 2, *r* = 0.50, *t* + 1/*n* = 0.45, and *r* is again at least that: **not met**. The value is the same on both readings. Under 5.1 a rate above the threshold settles Route 1 whatever the gate, so the gate of 5.3 is not reached, as in 12.1. No contribution with a third party as its bearer occurs in the extract in any case: B8 and B14 name Jonas, but their bearer is P, so there is nothing for the gate to count. *n* is far below the fifteen of 3.5 and the figure is reported for the exercise.

*The triple (6.1) and the availability gate (6.2).*

| Element | Where | Standing |
| --- | --- | --- |
| **T** | B8 | D+ under 4.2, so **V2** holds: there was a proposition to correct. |
| **K** | B9 | **V1** holds: K states in plain words that the attribution is wrong (“That is wrong. I was not angry, and it has nothing to do with Jonas”) and what is the case instead (“I was frightened, because I thought something had happened to you”). No irony, implication or display of feeling carries it. |
| **V3** | B2 on day 1, B6 on day 4 | Two dated earlier occasions, in the same channel and register as K, on which R answered on the content of what was said. Holds. |
| **L** | Occasion 5, B13 to B16 | **V4** holds: P referred to the situation and the state T was about, R’s lateness and being frightened by it. Under 6.5 the occasion is a different occasion under 3.3, in a different encounter, and nine days after K, so it is later and independent. |
| Mark on L (6.3) | B14 | **Re-asserted**: a contribution restates T in its uncorrected form. The marks are applied in order and the first that fits is the only one given. |
| The correction (6.4) |  | **Not carried**: at least one L is *re-asserted*. |

*B15.* “I told you it is not that” says the attribution is wrong and not what is the case instead, so it does not meet V1 on its own and is not a second correction. A second correction would in any case need a qualifying L of its own, and none follows in the extract. It raises no count.

*The repetition requirement (6.4).* Re-assertions documented on separate qualifying occasions: one. Required: two, either of the same corrected attribution or of two distinct ones. Not reached. One correction is assessable and the requirement is not reached: **Route 2 not met**. The counts are reported as they stand: corrections assessable 1; qualifying occasions 1; *re-asserted* 1; *carried* 0; *cited* 0; *neither* 0.

*Route values.*

- **Route 1 (5.1): not met** on the extract, the rate lying at or above *t* + 1/*n* on either reading of the boundary.
- **Route 2 (6.4): not met.** The correction is not carried, one re-assertion is documented, and two are required.

*Decision (Section 7), on the extract as the whole record.* Line 1 decides: the minimum material of 3.5 is not met (five assessable occasions across four encounters, below fifteen) and Route 2 is not met. **Not classified**, for want of material, with the shortfall reported and both route values and the counts recorded. Were the same route values to hold on a record meeting 3.5, line 4 would decide: **responder condition not met**, reported with the counts above. It falls as the patterns of 12.6 fall: one re-assertion is documented where 6.4 requires two. What would move the dyad to line 2 is a second documented re-assertion: either T set again in its uncorrected form on a further occasion at least seven days after K and different from occasion 5, or a second corrected attribution re-asserted once on a qualifying occasion of its own. Line 2 would then decide even below the minimum of 3.5, because Route 2 rests on documented re-assertions and not on a rate.

**12A.3 What the two transcripts exercise, and what they do not.**

Of the decisions listed in 11.3, the transcripts exercise occasion boundaries (3.2, 3.3); assessability on Q1, through the test of every perceiver line against 3.1, and on Q5, through A10 (3.4); the D-test with all three clauses and the anchors of 4.3 (4.2); the intimacy ladder (5.2); the selectivity gate (5.3); the availability gate (6.2); the carriage decision (6.3); and the outcome of Section 7. They do not reach the correspondence score of 9.2, which needs a probe and not a record. Three further rules are exercised that 11.3 does not list, because they are not rater decisions on one occasion but rules applied over a whole record: the rate and its margin (5.4), the repetition requirement (6.4) and independence of L (6.5). Q2, Q3 and Q4 hold throughout, so no failure of them is exercised. Nor are the marks *carried*, *cited* and *neither*, the silence rule and the sixty-second rule of 3.2, the elicitation of Section 8, or the correspondence score of 9.2, for which no probe episode is present.

One point in the coding remains open, and it is narrow: the novelty mark of 4.4 where the content appeared earlier in the perceiver’s record only as a denial (B14), which touches no decision, since 4.4 enters none. Two points that a rater might take for open are decided. The occasion rule of 3.3 decides both a second content arriving inside an open window and the position of the sixth responder contribution, and the tables follow it, as they follow it for the restatement of an opening content inside its own encounter, which B9 exercises and the boundary note there records as the place a rater is likeliest to depart from the rule. And A10 is D? under 4.2(a), which decides it: where the wording names no bearer, position after a content does not fix one, so a rater who took the placing of a generic-form contribution directly after a content as fixing its bearer would be departing from 4.2(a) as written, and a discrepancy of that kind is reconciled against the rule; 11.3 reports the D? rate by rater because a D? removes an occasion from the denominator. Under 11.1 a discrepancy that cannot be reconciled against a rule is a defect in the manual and is recorded as such, and 11.4 uses training material to repair unclear rules rather than leaving them to the rater; the novelty mark of 4.4 is recorded on that footing and is named again where it arises in the coding.

## 13. What this manual does not do

It does not measure understanding, felt or demonstrated; Section 9 does that, separately and behind a blind.

It does not establish that the responder condition predicts anything. The surplus of felt over demonstrated understanding is a prediction of the main text and not a coding criterion, and nothing in Sections 4 to 7 refers to it. Dyads can be coded “met” and show no surplus at the rate main text H1 states, and, once the probe has met the evidential standard of main text 3.4 under 9.5, the account is then wrong.

It does not check whether what a responder says about a third party is true. The gate of 5.3 scores determinacy and never accuracy, by design.

It does not code attributions borne by actions rather than by utterances. Deciding that an act presupposes a reading of the perceiver’s state would require a rater to infer a mental state from behaviour, which the fourth limit of Section 1 forbids. No occasion is entered as low because a rater judged what an act presupposed.

It does not distinguish the reasons a responder’s attributions are sparse. Those reasons govern what is to be done and never whether the condition obtains.

## 14. Design details for the hypotheses of main text Section 3.4

The main text states each hypothesis in its design, independent variable, dependent variable, prediction and falsification condition. What follows is the protocol detail those statements rest on. Nothing here changes a prediction; it fixes how each is to be run.

**14.1 The bounds, and the values they are reported at.** Every bound is a planning value proposed by the author and none is a threshold whose clinical relevance has been established. Every reported quantity is an expectation implied by the fitted model rather than one of its coefficients (14.11). This position is the single place where a bound is assigned to a prediction; where another file names a bound, it is this list that governs.

| Family | Value | Reported again at | Applies to |
| --- | --- | --- | --- |
| Contrast of directional bias between conditions or arms | 0.3 points | 0.2 and 0.4 | H2 (both block contrasts), H4, H5 (i), H5 (iv), which carries it on each of its two estimands and on each route, the first estimand being a change over waves within the met dyads and not a contrast between arms (14.13) |
| Contrast on the felt component alone | 0.3 points | 0.2 and 0.4 | H2 (additional prediction), H5 (iii), which the main text states as a within-person change in that component and not as a contrast of directional bias |
| Difference in correspondence between the probe and the modified video-cued procedure | 0.15 points | 0.1 and 0.2 | the comparability criterion of 9.5, read as an equivalence question; set at half the contrast bound, see below |
| Quantity scaling with a continuous predictor | 0.1 points per standard deviation | 0.05 and 0.15 | H1, H2 (the widening with readiness), H5 (i, the rise with readiness), each cross-partner path of 14.13 |
| Slope difference | 0.1 points per point of demonstrated understanding | 0.05 and 0.15 | H5 (ii), read against the comparison block |
| Attributed depth | 0.2 standard deviations of the depth change score of 14.6 | 0.1 and 0.3 | H3 |
| Proportion | 5 percentage points | 3 and 8 | accuracy-demand rate and cue-recycling proportion of 9.9 |

The per-standard-deviation value is set at a third of the contrast value, so that a spread of three standard deviations in the predictor would have to move directional bias by less than the contrast bound. Where a conclusion changes across the reported range it is reported as bound-dependent. The sample-size consequence is set out in Supplementary Material S2, Section 10.

The comparability bound is set at half the contrast bound, and the reason is stated. It is a bound on measurement equivalence and not on an effect: a difference between procedures up to the bound is tolerated as a property of the instrument, so it has to lie inside the smallest difference the hypotheses treat as substantive on the metric the contrasts are read in, or the instrument could produce or remove a surplus of exactly that size and still pass. The 0.15-point tolerance is numerically half the 0.3-point substantive margin; it bounds the average difference between the procedures and not the bias in a contrast between conditions or in a change over time, which offsets running opposite ways in two conditions could exceed. The value is a planning convention of the author’s and not a finding, and it is reported again at 0.1 and 0.2. The per-standard-deviation and slope bounds are in other units and are not compared with it. What the mean comparison cannot detect is a shift that differs by readiness or by condition; that is the demand and reactivity of 9.5, and it is assessed there, by the rates recorded and by the additional evidence the protocol fixes.

**The four outcomes.** Every reported quantity carries a 90 per cent interval, computed by the delta method or by simulation from the fitted model (14.11), or for H5 (iv) by resampling complete dyads (14.13). The interval is read against the bound and against zero, and exactly four things can happen. *Supported*: the interval lies entirely beyond the bound in the direction the hypothesis predicts. *Falsified*: the interval lies entirely inside the bound, which is the equivalence result and is reported as evidence of absence rather than as absence of evidence. *Opposite*: the interval lies entirely beyond the bound in the other direction, which falsifies the hypothesis and is reported separately from the equivalence case, because a reversal is a different finding from a null and must not be recorded as support. *Undecided*: the interval covers the bound, or covers both zero and the bound, in which case the hypothesis is reported as untested on that sample and no directional claim is made. Those four readings are for a hypothesis that predicts a difference, which is every prediction in the package but one. Two of them carry a further condition for H5 (iii): the *falsified* and *opposite* readings have the consequence main text 3.4 attaches to them only where the implementation standard of 9.8c was met, and where it was not, the same interval is reported under 9.8c and is neither evidence of absence nor a reason to withdraw. A second condition holds for H1 and for H5 (iii) and (iv) alike: support or withdrawal at the level of the construct requires prior evidence supporting the probe’s intended use under 9.5, of which the comparability criterion is a necessary part and not the whole; until that evidence is in hand, every reading, the *supported* one included, is reported as a finding about the procedure and the mechanism as not adequately tested. H5 (iv) predicts an absence of decline, and there the reading is reversed and must be, or the rule would report the prediction’s own success as its falsification. It is a claim of non-inferiority and it is read on two estimands: the change over waves in directional bias at c within the dyads meeting the responder condition, and the difference between that change and the same change in dyads not meeting it. Each carries the contrast bound of the table above and each is read the same way: supported where the lower limit of its 90 per cent interval lies above minus the bound, falsified where the whole interval lies entirely below that value, and undecided in every remaining case, which is every interval that covers minus the bound, whether or not a limit sits exactly on it. H5 (iv) is read by route under 14.13, and on each route it is supported only where both are supported, falsified where either is falsified, and undecided otherwise. Where the first estimand is supported, an interval lying wholly above plus the bound on the second is not a case to be discarded but the predicted pattern in its strongest form, the surplus holding in the met dyads while it falls in the others, and it is reported as such. 14.13 states the two estimands, the rule joining them and what is reported beside them. Which reading applies is fixed by the direction of the prediction and not chosen after the interval is seen.

The equivalence logic follows Lakens (2017); the intervals here are on model-implied expectations rather than on raw effect sizes, and that extension is the author’s and is not covered by the cited treatment.

**14.1a The error rate across the family.** Five hypotheses are tested, H5 alone carries four predictions, and every result is reported again at two neighbouring bounds. No correction for multiplicity is applied, and the reason is stated rather than left to be assumed. Each hypothesis carries its own prediction with its own direction and its own bound, fixed before collection; none is a search over a family for whichever comparison reaches a threshold, and the neighbouring values are a sensitivity report on one result and not additional tests. What this leaves open is real and is acknowledged: across the nine predictions and the four further bound-read quantities, the two cross-partner paths of 14.13 and the two rates of 9.9, the chance that at least one interval falls where it was not expected is not small, and a single supported prediction in an otherwise null set is not evidence for the account. H5 (iv) is read once per route under 14.13, which doubles its intervals and adds no prediction. The comparability criterion of 9.5 is a precondition on the probe and not a member of this family. The account is supported where the pattern holds across H1, H2 and H5, with the paths and rates reported beside it; for H5 the pattern holds where (iii) and (iv), on every route adequately tested, are supported, and where either is undecided and neither falsified the account is reported as not adequately tested on that sample, neither supported nor withdrawn. Section 3.4 of the main text says which single failures withdraw the construct. A study that took one of the nine and reported it alone would be reporting something this design does not claim.

**14.2 The conditioning level.** Conditional comparisons are taken at c = 1.0, the midpoint of the metric, fixed before analysis, and reported again at 0.5 and 1.5. Where the sign of a conditional difference changes across that range it is reported as sign-dependent and carries no directional claim. The reason for fixing it is that H5 (ii) predicts different slopes, and a conditional difference taken where slopes differ depends on where it is taken.

A conditional quantity needs a value for every term in the model and not only for demonstrated understanding, or it is not a number. That includes the random effects. Every model-implied expectation reported here is taken marginally over the random-effects distribution, by numerical integration or by simulation from the fitted variance components, and not at random effects set to zero. The two differ in a non-linear model, and the choice is not arithmetic: the hypotheses are about populations of dyads, so the quantity wanted is the population-averaged expectation and not the expectation for a dyad sitting at the centre of the distribution. Where a dyad-level quantity is wanted instead, as in the tracking slope of a single dyad, it is named as such and reported as a conditional-modes prediction. The values are fixed here: the two readiness facets and attachment anxiety at the sample mean of the analysis sample; the responder condition at *not met*, which is the reference level of 14.3; the perceiver’s own rated responsiveness at its sample mean, which is on its own instrument and not on the three-point metric, so no value of c applies to it; episode at the mid-course anchor of 14.12; every remaining covariate at its sample mean, or for a categorical covariate at its most frequent level, named in the protocol. A comparison between conditions is the difference between two such quantities taken at exactly these values. Where a quantity is taken from a fit within one set, as the two estimands of 14.13 are, the condition variable of 14.3 is absent from that fit, every other value in this list stands unchanged and is taken from the pooled sample of the sets compared, and the set is named with the estimate. Where a product term is fitted, the quantity is reported additionally at one standard deviation above and below the mean of the moderator, because with a product term there is no single directional bias and a value read at the mean is not the whole result.

**14.3 H1, the estimand and the coding of the responder condition.** The tested quantity is the product of the threshold facet of Supplementary Material S2 and the responder condition, adjusted for the motivation facet, for attachment anxiety and for the main effects of the threshold facet and of the condition; the bound of 14.1 applies to that product and not to the main effect, which is reported beside it as secondary. The condition is three-valued under Section 7, met, not met, and not classified. It enters as two indicator variables with not met as the reference, and dyads that are not classified are pooled with neither level but carry their own indicator, reported and not interpreted as a degree of the condition. Where their number is too small for a stable estimate they are dropped, and that is reported as selection on codability rather than as a missing-data adjustment, because the material they failed to supply is the material the first route is coded from, so their exclusion is not independent of the predictor. The sensitivity analysis refits the model with the unclassified dyads assigned to each level in turn and reports both results alongside the primary one.

*Which term carries the bound.* The condition enters as two indicators (met, not classified), with *not met* as reference, so the model contains two product terms. The bound of 14.1 applies to the product with the *met* indicator and to that alone; the product with the *not-classified* indicator is reported and is not a test of the hypothesis, since not classified is an absence of evidence and not a degree of the condition. The estimand is the difference between two model-implied slopes, each in points per standard deviation of the threshold facet, computed at the values of 14.2 and read at one standard deviation either side of the facet mean.

*Direction, and what opens an occasion.* Two points fixed here. The outcome of H1 carries no valence: a perceiver may credit his partner with grasping something unflattering as readily as something kind, unlike the negative readings attachment anxiety predicts (Collins, 1996; Overall et al., 2015), and no prediction here turns on the content being favourable. And the perceiver’s written probe contents are private: they open no occasion under 3.3, which opens at a contribution, so the occasions a dyad supplies are opened by what is spoken in the episode and not by what is written beside it.

*How much probe material, and who is the perceiver.* Two values the design needs and no other position fixes. **Probe episodes:** at least four per dyad, spread across the observation period rather than clustered, each meeting the ten-minute minimum of 9.1. Four is the smallest number that leaves a within-dyad tracking slope estimable on more than a handful of contents, since 9.1 admits at most three contents per episode; where a dyad yields fewer than four, its slope is reported under the rule of 9.3 and the dyad still contributes to the conditional quantities. **The perceiver role:** both partners are assessed in both roles wherever the material allows it, and the dyad then contributes two sets of observations with a random effect for the dyad above the person. Where only one direction can be run, the role is assigned at random and the assignment is recorded. It is never assigned on readiness, on talkativeness or on who volunteered, because each of those selects on a predictor or on its correlate and would build the association H1 tests into the sample.

*Codability against readiness.* The three values of the condition are cross-tabulated against readiness, and the association between them is reported before the product term is interpreted. The reason it cannot be passed over is internal to the account: the second route is assessable only where a correction was stated, path P2 of main text 3.2.3 predicts that the rate of corrections falls as the configuration holds, and where the second route drops out the first decides, which requires the selectivity gate of 5.3. Codability is therefore expected to covary with the very moderator H1 estimates in the product term. The elicitation step of 8.1 exists to reduce that dependence and is not assumed to remove it; where the association is substantial, the product term is reported as conditional on codability and the limitation is stated with the result.

**14.3a The analogue, and the words it uses.** H2 and H5 run a single-session analogue, and three words have to be kept apart there or nothing in 9.8 can be executed.

A **condition block** is one of the three assigned conditions of 14.4. A participant passes through all three, in the order fixed by 14.14. A **segment** is one occasion-equivalent inside a condition block, closed by an intervening task that ends the exchange, as 6.5 requires. Each condition block carries **seven segments**: six of history and one scored episode. A **later segment** within a condition block is what stands in for a later, independent occasion.

Two consequences follow, and they are the point of this position.

First, the seven-day interval of 6.5 is not met inside a session and is not pretended to be. In the analogue the responder condition is **assigned by protocol and not coded**: the confederate is instructed to re-assert or to carry, and the experimenter knows which. Sections 3.5, the margin rule of 5.4 and the decision order of Section 7 are therefore **not applied** in the analogue, and no analogue block is ever reported as “met” or “not met” under Section 7. What the analogue manipulates is the operational counterpart of each route, and what it supports is a claim about that counterpart. The step from there to the coded condition of main text 3.1 is an assumption the analogue does not test, and the field module of 14.13 is where the coded condition does the work.

Second, every rule below that speaks of blocks in the criterion sense is to be read as speaking of segments: the two documented re-assertions of 6.4 fall on two later **segments** of the same condition block, not on two condition blocks, and the gate contributions of 5.3 are distributed across at least two **segments**.

**14.4 H2, why the two dimensions are not crossed.** Specificity and persistence cannot be varied orthogonally. In a block where no determinate attribution is set there is nothing to correct, so persistence has no referent and two cells of a crossing would be empty by definition. The design therefore runs three conditions, which map onto the criterion of Section 7: determinate attributions set and corrections carried (the condition not met); determinate attributions set and corrections re-asserted uncorrected on later segments of the same condition block (second route); determinate attributions set at a rate below the threshold (first route).

The third condition reaches the first route only if the selectivity gate of 5.3 is passed inside it, and a script that simply says less does not pass it. The low-rate condition block therefore carries scripted D+ readings of identified third parties at intimacy level I3, at least three of them across at least two segments, so that the gate of 5.3 is passed inside the block. The segment stands in for the encounter of 5.3 here, as it stands in for the occasion elsewhere in the analogue (14.3a); the substitution is looser than the field rule, since two segments of one session are not two encounters, and it is made because a single session cannot supply encounters at all. That is a further respect in which the analogue reaches the counterpart of the route and not the route. The rate itself is set by the script and not estimated: with seven segments the granularity rule of 5.4 would admit no determinate attribution at all, which is why the analogue assigns the low rate rather than coding it (14.3a). That third-party content is held constant across all three conditions, so it cannot itself account for a difference between them. Where a protocol cannot hold it constant, the third condition is labelled “low rate, gate not applicable” and the analogue is reported as reaching the second route alone.

**14.5 H2, why matching is done after the fact.** The perceiver’s contents are freely produced and locked before the responder replies, under 9.1. A scripted reply therefore cannot be written to a guaranteed correspondence score, and no block can be equalised on demonstrated understanding by construction. The prepared responder entries are drawn from one and the same prepared set in all three conditions, as 9.8 already fixes for the H5 contrast, and are written to span the range. This is what makes the matching admissible: if the entry set differed by condition, the obtained score would be a function of the condition and stratifying on it would be conditioning on a post-treatment variable, which the manipulation moves by design, since demonstrated understanding is itself lower where corrections are dropped. Holding the entry set identical removes any difference between conditions in the response materials themselves. It does not establish that the obtained score is unaffected by the assigned history, which can bear on what the perceiver reports in the scored episode and so on the pairing of entry to content. The obtained scores are measured, blocks are stratified on them, and the comparison is made within strata. *The multilevel structure of the analogue.* Contents nest within the scored episode, the scored episode sits in a condition block, and the three condition blocks sit within a participant. Random intercepts are fitted for the participant and for the condition block within the participant; the condition itself is a fixed effect, since every participant meets all three. The dyad level of main text 3.5 has no counterpart here, the responder being a confederate or a script, and the participant takes its place.

*Conditioning and stratification are not run twice.* Main text 3.5 reads every conditional quantity at a fixed level c, and this position matches blocks on the obtained level of demonstrated understanding. Doing both literally would condition on the same variable twice. The primary analysis therefore enters the obtained level as a predictor centred at c, which is what conditioning at c means in a model, and reports the contrast at that level; the literal stratification is run alongside as a sensitivity analysis and reported with it. Where the two disagree, both are reported and neither is preferred, since the disagreement would itself be evidence that the matching is doing work the model does not.

*Which facet widens the gap.* The widening with readiness is carried by the threshold facet of S2.5, the same facet that carries H1, with the motivation facet entered beside it and never pooled with it. Main text 3.4 says “readiness” in that sentence, and this position fixes which part of it is meant.

The residual assumption is stated rather than hidden: the pairing of a fixed entry to a freely produced content is taken to be independent of the assigned condition, which the design makes plausible and does not establish, and the unstratified contrast is reported alongside so that the effect of stratifying is visible.

**14.6 H3, the depth measure and the diary arm.** *The adapted items.* The four items of S2.6 are administered in H3 in a vignette-referenced wording that S2 does not validate; their reliability and their separation from the anchors B1 and B2 are reported from the vignette pilot named in Section 10 before the test is read, and a *falsified* reading of H3 is reported as conditional on that measure. *The predictability index.* It is the absolute value of the lag-one autocorrelation of the valence sequence, computed within person over the episodes of the arm, and it is not a dispersion measure. The absolute value is essential and not a refinement: a strictly alternating order of warm and cold is the most predictable sequence the design can produce and its signed autocorrelation is close to minus one, so a signed index would order the two arms the wrong way round. What the index reads is how far the next valence can be extrapolated from the last, in either direction. A sequence of constant valence is not admitted by the vignette design, which fixes six warm and six cold episodes, so the degenerate case in which the autocorrelation is undefined does not arise. That distinction is the point: the vignette arm holds the multiset of episodes identical across conditions, so mean and dispersion are equal by construction and only the order differs, which is exactly what an autocorrelation reads and what a dispersion index cannot. The difficulty that a dispersion measure depends on its mean (Mestdagh et al., 2018) therefore arises only in the diary arm, where mean warmth is not controlled, and it is there that mean warmth is carried as a covariate with its interaction. *The bound.* The bound of 14.1 for H3 is 0.2 standard deviations of the depth change score, estimated in the same sample; where a standard deviation is named for H3 anywhere, it is that one. *Episodes, and the two orders.* Twelve episodes per sequence, six warm and six cold, the same twelve in both arms, with condition assigned between persons so that no participant sees both orders. Three constraints hold in both arms alike, so that the arms differ in the order of the episodes and not in the valences the change score is taken on. First, the valence of the first episode is counterbalanced one to one within each arm, half the participants of an arm beginning warm and half beginning cold. Second, the valence of the twelfth episode is the opposite of the first in every sequence of both arms. Third, the remaining ten positions carry the five warm and five cold episodes that are left. The cyclic arm uses strict alternation, warm and cold turn about, which satisfies the second constraint by construction, is the most extrapolable order the multiset admits and gives an absolute lag-one autocorrelation near 0.92. The random arm draws a fresh permutation of the ten inner positions for each participant and rejects any draw whose absolute lag-one autocorrelation over the whole sequence exceeds 0.3, so that the two arms are separated by construction and not by chance; the realised value is recorded for every participant. The randomisation of that arm is restricted in exactly that way and is described as restricted wherever it is reported. What the three constraints remove is the confounding of arm with the valence of the two episodes the change score is read on, which without them would differ between the arms by construction in the cyclic case and by chance in the random one. They do not control every other property of an order, and no claim of complete control of sequence effects is made here or anywhere in the package.

*What the index is for.* The independent variable is the assigned arm and nothing else. The index is a construction control: it confirms that a particular draw fell in the intended range, and nothing is regressed on it in the vignette arm.

*One analysis.* The change score is the rating after episode twelve minus the rating after episode one, and the test is a regression of that change score on the arm, with no other term. That is the analysis the bound of 14.1 is stated for, since the bound is in standard deviations of the change score and a mixed model over positions would carry no such quantity. A mixed model with arm, position and their interaction may be reported alongside as a description of the trajectory, and it decides nothing. *The unit of the diary arm.* The day. One entry per day over four weeks, each recording the valence of the day’s most salient exchange, so that the valence sequence is a sequence of days and not of episodes. Here the index is not a check on an assignment, since nothing is assigned, and it is the predictor; a participant whose valences are constant over the four weeks yields no autocorrelation at all and is reported as contributing none, which is a limitation of the arm and not a missing value to be filled. *The model.* Attributed depth is the mean of the four adapted items of S2.6 on their six-point format, which is treated as continuous. The primary test is the regression of the change score on the arm, stated below, and that is the analysis the bound of 14.1 is set for. A linear mixed model with a random intercept for the participant, the arm as a between-person effect, the position in the sequence as a within-person effect and their interaction may be reported beside it as a description of the trajectory; it decides nothing, and a model without that interaction would test a difference in level rather than the increase the hypothesis is about. Neither is a cumulative-link model: the quantity is a mean of four items and not a single ordered category, and the reasoning of 14.11 does not reach it. The difference between arms is therefore an expectation on the six-point scale, and the bound of 14.1 for H3 is in standard deviations of the change score for that reason. Attributed depth in the vignette experiment is the mean of the four-item adaptation of S2.6, worded for the responder of the vignette rather than for a present partner and rated on the same six-point agreement format after each episode. The change score is the last rating minus the first, and the number of episodes is fixed in the protocol before the arms are written. The bound of 14.1 for this hypothesis is in standard deviations of that score, estimated in the same sample and reported with the result, because the depth score is not on the three-point metric and the 0.3-point bound does not apply to it. The four-week re-administration rule of S2.10 governs the partner-referenced items and not this adaptation, which refers to no standing relationship. Mean warmth and predictability are kept apart and never entered as if independent. Mean warmth is carried as a covariate with its interaction with the predictability index. The dependence of a dispersion measure on its mean is a known difficulty of such indices (Mestdagh et al., 2018) and this design does not claim to remove it. The index is validated within the arm against the participants’ own predictability ratings, and its relation to mean warmth is examined and reported, before any incremental effect is interpreted; where that validation fails the diary arm is reported descriptively and contributes no test. Two checks run and they answer different questions. The participants forecast each next valence and rate the predictability they perceive, which is the manipulation check proper: it says whether the assigned order was experienced as more or less extrapolable. The absolute lag-one autocorrelation of the realised sequence is a construction control, not a check: in the vignette arm it can hardly fail, because 14.6 separates the arms by construction, and its use is to confirm that a particular draw fell in the intended range. Main text 3.4 names both, and the first is fixed here.

**14.7 H4, what the first block must contain.** The responder condition cannot be coded at a single baseline. The first route requires the minimum material of 3.5, fifteen assessable occasions across at least three encounters; the second requires a qualifying later occasion under 6.5, at least seven days after a correction. The block requires both, whatever the route on which the condition is then met, because the analysis carries the coded rate of 5.4 alongside the condition. That is an entry requirement of this design and not a qualification of Section 7, which classifies a dyad met on the second route below the minimum of 3.5. The first block runs for a maximum of twelve weeks or twenty recorded encounters, whichever comes first, fixed before recruitment. Where the minima of 3.5 and a qualifying later occasion are both reached before that, the block closes then; where they are not reached by the limit, the block closes anyway and the dyad is reported as not classified for want of material. The limit exists because a block that ran until the material was sufficient would be a stopping rule that reads the outcome, which 9.3 forbids everywhere else. Corrections the perceiver states to the responder are logged throughout.

**14.8 H4, the no-feedback arm and the baseline covariate.** Dyads are assigned to a feedback arm and a no-feedback arm within each level of the responder condition. Neither group is selected on its baseline discrepancy, and that value is entered as a covariate for both, because the outcome is change in the same quantity and selection on it would produce the predicted movement by regression to the mean alone. The no-feedback arm measures the size of that regression directly.

**14.9 Safeguards wherever a release is made or the prompt of 8.1 may be put.** H4 is the only arm in which a release is made (14.12), and it puts the scored record of what a responder missed in front of the perceiver. Dyads are screened at intake for intimate partner violence and for coercive control with an established instrument named in the protocol. A positive screen excludes the dyad from every arm in which a release is made and from the elicited correction prompt of 8.1, which asks the perceiver to put a reading right to the responder in so many words and is therefore the operation main text 5.4 warns against where a relationship is unsafe: a reality test must not become a confrontation the perceiver has to survive. Such dyads may contribute observational material with neither, and their spontaneous corrections are coded like any other, since what 8.1 adds is the prompting and not the coding. Screening is repeated and not confined to intake: the instrument is administered again before every wave of the field module, before every observation period at the end of which the prompt of 8.1 may be put, and before the release of 14.12 in H4. A positive screen at any later assessment withholds both the release and the prompt from that point, the dyad’s later occasions form a separate stratum, and the count of dyads so withheld is reported by route; their spontaneous corrections are coded like any other, as above. Every perceiver who receives a release is debriefed at the end, told what was withheld and why, and given the contact details of a service. The analogue of 9.8 carries a debriefing of its own, and it is not optional: the responder there is a confederate or a script, the wrong attributions of 9.8b are planted, and every participant is told so at the end of the session, shown which contributions were prepared, and given the opportunity to withdraw his data. The deception and the debriefing are part of the ethics application. One item is asked of every participant and its answers are reported: whether he noticed that the responder contradicted, within the same session, a reading the responder had himself spoken minutes earlier. That is what the design requires of the confederate, it is the least plausible thing he does, and a participant who noticed it was not in the situation the analogue is meant to create.

**14.9a H5, what stands as the accuracy term.** The rival of main text 3.4 H5 carries an accuracy path from the partner’s actual responsiveness, and prediction (iii) asks for an increment over it. That term needs a measure, and the main text names none. It is the responder’s scored demonstrated understanding in the same episode, from 9.2: in the analogue it is the score obtained on the prepared entries, in the field module the score obtained on the probe. That is the quantity the rival’s accuracy path is about, it is measured for every episode already, and using it means the increment asked of (iii) is an increment over what the responder actually demonstrated on that occasion. The projection term is the perceiver’s own self-rated responsiveness (main text 3.5), the investment term enters in the field module alone (14.10), and the model for (iii) carries the projection and accuracy terms in both arms and the investment term in the field module alone.

*When the projection term is measured, and what it is rated about.* In the analogue it is taken once, before the first condition block and so before any manipulated history, and that single unchanged value enters the model for (iii) as a person-level covariate; in the field module it is taken at every wave and the first-wave value is the baseline. Three things about it are fixed in the protocol before collection, because a self-report of one’s own responsiveness has no determinate meaning without them: the person and relationship it is rated about, which in the field module is the standing partner, and in the analogue is not the responder of the session, who is a confederate or a scripted system with whom the participant has no history to report on, but the participant’s own conduct in his close relationships generally; the period it covers, which in the field module is the four weeks before the rating, the interval the re-administration rule of Supplementary Material S2 already uses, read over the standing relationship, and which in the analogue is not a bounded period, the dispositional wording asking how he characteristically conducts himself in his close relationships; and the wording of the items, which is the own-responsiveness set of Lemay et al. (2007), in the field module with the standing partner named in the stem and nothing else altered, and in the analogue in a dispositional adaptation of the same items, whose wording is fixed and piloted before collection and reported as an adaptation and not as the original instrument. A value taken after a manipulated block is a quantity of the kind 9.8c sets apart, since the assigned history can move it, so such values are used only in process or sensitivity analyses labelled as such and never replace the baseline in the primary model. What prediction (iii) then reports is an increment over that measured baseline responsiveness. It is not the exclusion of projection, and it is stated in those words wherever it is reported.

**14.10 H5, the manipulation check and the Investment Model Scale.** After each block, participants rate whether the responder took up what they had put right; blocks the check cannot separate leave the test uninformative. *The item, its scale, and what counts as separation.* The check is one item put after each condition block, worded in the protocol and identical across blocks: how much of what the perceiver had put right the responder then took up. It is scored 2 where he names both the correction and an uptake of it, 1 where he names one of the two, and 0 where he names neither. Those three values count two named elements and are not the correspondence metric of 9.2, which scores a rater’s reading of a record rather than a participant’s report; nothing on this item enters a correspondence score, a rate or a gate. A participant’s two blocks are separated where the score in the block assigned carriage exceeds the score in the block assigned re-assertion by at least one point, and are not separated where the two are equal or run the other way. The proportion of participants whose blocks are separated is the first of the three quantities of 9.8c; it is reported by the order of the blocks and by level of readiness, and like the rest of that standard it is computed and recorded before any estimate for prediction (iii) is calculated. An unseparated pair is excluded from nothing. It enters both analyses of 9.8c like any other, the three rules named there remain three, and the only way it bears on the result is by lowering that proportion. The Investment Model Scale (Rusbult et al., 1998) is administered in the field module alone, where a standing relationship gives its terms a referent. A single-session analogue has none, and it is not administered there.

**14.11 The ordinal metric, and the scale the results are reported on.** The three-point scale of 9.2 has ordered categories and no established spacing. Primary models are cumulative-link mixed models with a logit link. Their coefficients are not the reported quantities. What is reported, and what the bounds of 14.1 apply to, are the expectations those models imply on the observed metric: expected felt understanding at a stated level of demonstrated understanding and stated covariate values, obtained as the sum over the three categories of the category value times its fitted probability. Directional bias at c is that expectation minus c. A quantity in points per standard deviation of a predictor is likewise a difference of two model-implied expectations: the expectation with that predictor one standard deviation above its sample mean, minus the expectation with it at its mean, every other term held at the values of 14.2. It is taken at the mean and not averaged over the range, because under a logit link the expectation is not linear in the predictor and a step taken elsewhere on the scale would give a different number against the same bound. The same rule gives the H1 estimand of 14.3, the widening terms of H2 and of H5 (i), and each cross-partner path of 14.13. The tracking slope is a discrete difference and not a derivative, because the predictor takes three values and a derivative would describe a scale the data do not have. It is taken on the within-episode part of the decomposition of 14.12 and on that part alone, since moving the episode mean or the dyad mean by a point gives a between-unit slope of a different value and a different meaning. At c = 1.0 and c = 0.5 it is the expectation at c plus one point minus the expectation at c; at c = 1.5 it is the expectation at c minus the expectation at c minus one point, because c plus one would fall at 2.5, outside the metric. Under a logit link a difference so taken is not invariant to a shift in level, and H5 (i) predicts exactly such a shift between the conditions H5 (ii) compares. The slope difference is therefore reported twice, once at the values of 14.2 and once with the level of felt understanding equated across the conditions, and where the two disagree in sign, or in whether the interval clears the bound, H5 (ii) is reported as level-dependent and carries no directional claim. For the same reason (i) and (ii) are not two independent supports for the account, and the main text says so in saying that they settle nothing alone. Mean-level discrepancy is the average over contents of felt minus demonstrated understanding, computed once and in that way wherever it appears. A contrast is the difference between two such expectations. All three are in points, and intervals for them are obtained by the delta method or by simulation from the fitted model; for the (iv) estimands of 14.13, whose sets may share dyads, the interval is obtained by resampling complete dyads, as 14.13 states. Linear models on the same quantities are reported alongside as a sensitivity analysis, and where the two disagree in sign, or in whether an interval excludes a bound, the ordinal result stands. The reliability of an episode mean is estimated in the coding study and reported before any such mean is used.

**14.12 The probe: releases, matched levels, and the multilevel specification.** This position carries the detail main text 3.5 refers to. *The two estimands that turn on a release.* The H4 estimand is the change in directional bias at c from the episode before the release to the last episode after it, and not across the whole block: the strata either side are analysed apart, and only that span is the one the release could have moved. And in H5, felt understanding before the disconfirmation is not entered as a covariate on the test of (iii). Prediction (i) says that level differs by condition, so adjusting for it would remove part of the effect under test; the unadjusted contrast is primary, a covariate-adjusted version is reported alongside, and where the two disagree the unadjusted one stands.

*Releases.* A release is made in H4 and nowhere else. In the second block of H4 it is made once, after the perceiver’s ratings for the probe in the second episode are in; the first two episodes form the pre-release stratum and the remaining episodes the post-release stratum, and the two are analysed apart. No release is made in the field module of 14.13, in the analogue of 9.8, or in any observational arm, so those arms carry no release stratum; the release safeguards of 14.9 apply to H4 alone, and its screening and the withholding of the prompt of 8.1 run in every arm in which that prompt may be put. Episodes before and after a release are analysed as separate strata, and before a release the perceiver is never asked to notice what the configuration conceals, since the question would itself be the recalibration under test. *Matched levels.* Each condition being compared carries several matched levels of demonstrated understanding rather than one, so that the tracking slope is estimable within a condition and not only across conditions. *The slope of one.* A slope of one is neither assumed nor required, and on the metric of 14.11 it is not an informative case: the model-implied expectation lies within the range the metric spans, so a slope of one across that range would force the discrepancy to zero at every level. The mean-level discrepancy and the conditional discrepancy are separate quantities and neither is read as the other. The first is the average over contents defined in 14.11, taken across contents whose levels of demonstrated understanding differ; the second is read at the fixed c of 14.2, and under the logit link the expectation is not linear in the predictor, so neither is recoverable from the other. Comparisons across units whose levels of demonstrated understanding differ are therefore taken at a common level. *The decomposition.* Demonstrated understanding is centred at the fixed c of 14.2, so that the model-implied expectation at the intercept is the expected felt understanding at c, and it is entered as three parts: the dyad mean, the episode mean minus the dyad mean, and the content minus the episode mean. A slope fitted on the undecomposed predictor mixes all three, and only the last is the quantity the tracking claim is about; the coefficient on that within-episode part is the one that varies at random across dyads, and the tracking slope of H5 (ii) is read off it under 14.11. *Random effects.* Random intercepts are fitted for dyads and for episodes within dyads, the tracking slope is random across dyads, and the random effects are anchored at a mid-course episode. The middle level is the episode of Section 9 throughout, as main text 3.5 states, and never the occasion of 3.3: the probe contents that carry the scores are written and open no occasion. The intercept-slope covariance is a population parameter for heterogeneity between dyads and is reported as such. *The difference score.* In the linear sensitivity model, and there only, regressing the difference between the two components on the centred predictor gives the same quantity as the model-implied expectation at the intercept; in the cumulative-link model it does not, and no difference score is formed there.

**14.13 H5, the field module.** The analogue arm of H5 assigns the responder’s uptake of corrections; the field module carries the same predictions in standing relationships, where assignment is impossible and the responder condition is coded instead. *What a wave contains.* A wave is one round of data collection, and it carries three things: recorded interaction sufficient for the minima of 3.5 to be reached cumulatively, which in practice means at least three encounters under 3.2a; one probe episode under 9.1, from which the directional bias that H5 (iv) tracks is computed, so that every wave yields the quantity the prediction is about; and the S2 measures due at that interval under S2.10, point 10. A wave is not an occasion and not an encounter; it is the interval within which both are collected. *Waves and lag.* Six waves at four-week intervals, the interval set by the re-administration rule of Supplementary Material S2 and by the requirement of 6.5 that a qualifying later occasion fall at least seven days after a correction. Six is the minimum that leaves five lagged intervals, enough for a cross-lagged panel in which the paths are estimable and a trend across waves is distinguishable from a single shift. *Sample.* Both partners of established couples, sampled so that the coded rate retains its low end rather than being selected on it, with the condition coded from recorded interaction at each wave under Sections 5 to 7. *Range restriction.* The coded rate enters continuously and is never dichotomised, and the sampling and selection mechanism is documented. What follows names the case rather than gesturing at it. The restricted variable is the coded rate of Section 5.4. The selection expected on it is indirect and runs through willingness to be recorded and through codability, not through the rate itself, which places the design in Thorndike’s third case rather than his second; a direct selection on the rate would arise only if recruitment screened on it, which the protocol forbids. A correction is applied only where four things hold: the selecting variable is identified and measured, its unrestricted variance is available from a named external source stated in the protocol before collection, the regression of the outcome on the rate is linear over the unrestricted range, and the residual variance is constant over it (Sackett and Yang, 2000). The correction is applied to the slope of the linear sensitivity model and reported beside the uncorrected one, and never to the model-implied quantities of 14.11, for which no established correction exists. Where any of the four fails, no correction is made and the uncorrected estimate stands with the realised range of the rate reported alongside it. The assigned analogue arm remains the primary discriminant test and this module is secondary to it. *The unit of the lagged terms.* Every wave carries different contents, so a content score has no previous value of its own and nothing can be lagged at content level. The lagged predictors are wave aggregates: for each dyad and wave, the model-implied expectation of felt understanding at c and the mean scored demonstrated understanding over the contents of that wave’s probe episode. The standard deviation against which the per-standard-deviation bound of 14.1 is read for these paths is the between-dyad standard deviation of the predictor at the first wave, named in the protocol before analysis. *The cross-partner paths.* These are the two lagged paths of main text 3.2.3 and are stated there: from felt understanding at the previous wave to demonstrated understanding at the present one, where R1 and R2 would show, and from demonstrated understanding at the previous wave to felt understanding at the present one, where P1 and P2 would show. Each adjusts for the prior value of its own outcome. *Model comparison.* Both models are bivariate autoregressive models, with felt and demonstrated understanding as joint outcomes and each regressed on its own previous value, and Model A is Model B with the two cross-partner paths fixed at zero. Main text 3.2.3 gives the same two models in the same terms. The felt-understanding equation of both carries perceiver readiness, demonstrated understanding at the same wave and their product, alongside the autoregression; that is the moderation part, and it is present in Model A and in Model B alike. What Model B adds, and Model A sets to zero, are the two cross-partner paths.

*The predicted signs.* “In the predicted direction” needs a direction, and the paths take it from the mechanisms they stand for. The path from felt understanding at the previous wave to demonstrated understanding at this one is predicted **negative**: it is where R1 and R2 would show, and both say that a responder whose understanding is already credited is one from whom less accuracy is demanded and less recruited. The path from demonstrated understanding at the previous wave to felt understanding at this one is predicted **positive**, since a responder who demonstrated understanding gives the perceiver more to credit; the account’s own claim about it is not its sign but its size, and it is predicted to be smaller in dyads meeting the responder condition, which is reported as the interaction of that path with the condition.

The loop claim requires both cross-partner paths, and the rule is stated rather than left to judgement: both are supported only where each carries a 90 per cent interval lying entirely beyond 0.1 points per standard deviation in its predicted direction. That is what makes the comparison a likelihood-ratio test on two degrees of freedom; a single-outcome Model A would not be nested in Model B and no such test would be available. Both share the lag-1 structure and the centring and random effects of main text 3.5. No measurement-invariance test is run and none is needed: the quantities are observed scores and model-implied expectations over them, not latent variables, and what has to hold across waves is the scoring procedure, which 9.2 fixes and 11.3 monitors. The comparison is reported, and each path is reported separately with its own interval, because a test on two degrees of freedom can reach significance where one path alone contributes. *The accuracy demand.* Path R1 is driven by the withdrawal of the accuracy demand and not by felt understanding as such, so the accuracy-demand rate of 9.9 is carried at each wave and entered as a third lagged predictor alongside the two cross-partner paths. Where it mediates the path from felt understanding to demonstrated understanding, that is reported; the model is not interpreted as testing mediation, which the design cannot settle. *Bounds.* Each cross-partner path is a quantity that scales with a continuous predictor and carries the bound of 14.1 for such quantities, 0.1 points per standard deviation, reported again at 0.05 and 0.15. *The non-decline prediction, H5 (iv).* This is the claim the account is named for, so it is stated as a prediction of non-inferiority and not as a significance test. The quantity is directional bias at c, a model-implied expectation in points (14.11), computed at each wave. Two estimands carry it, both on that quantity and both from the separate fits of the route sets and the comparison set described below. The first is the change in directional bias at c from the entry wave over a common horizon of k waves since entry within the met dyads of a route set, taken from that set’s fit at the values of 14.2. Since entry waves differ between dyads, wave enters the fit as waves since entry, so that dyads are aligned on their entry; the horizon k is named in the protocol before collection (Section 10) and is the same for the met dyads and for the comparison dyads, so that the two changes are taken over the same length of observation and a difference between them cannot arise from one side having been observed for longer. A dyad with fewer than k post-entry waves contributes the waves it has, the number of dyads observed at the horizon is reported by route, and the change to each dyad’s last observed wave is reported beside the primary estimate as a sensitivity analysis. The second is the difference between that change and the same change, over the same horizon, in the comparison set, taken from its own fit at the same values. The first is what the maintenance sentence of main text 3.4 asserts; the second asks whether that course differs from the comparison dyads by more than the bound. Equal stability in the two groups satisfies both, so H5 (iv) does not on its own establish that the maintenance is specific to the configuration; the discriminant claim is carried by (iii), as main text 3.4 states. Neither settles the claim alone, and this is the reason both are read: a decline shared equally by the two groups satisfies the second while contradicting the first, and a surplus standing still in both satisfies the second while showing nothing specific to the configuration. The first wave is the entry wave defined below, not necessarily wave one, and the distribution of entry waves is reported with each estimate. Each estimand is a prediction of non-inferiority and is read as 14.1 fixes for that case, on the same contrast bound of 0.3 points and reported again at 0.2 and 0.4: supported where the lower limit of the 90 per cent interval lies above minus 0.3 points, falsified where the whole interval lies entirely below that value, and undecided in every remaining case, which is every interval that covers minus 0.3 points, whether or not a limit sits exactly on it. The rule joining them is stated rather than left to judgement, as it is for the two cross-partner paths above: H5 (iv) is supported only where both intervals meet that condition, falsified where either is falsified, and undecided otherwise. Where the first estimand is supported, an interval on the second lying wholly above plus 0.3 points is the predicted pattern in its strongest form, the surplus holding in the met dyads while it falls in the others, and is reported as such. Both estimands are computed on the same met dyads and waves, against the comparison set defined below, and no dyad is excluded from either on the value it takes at the first wave: prediction (i) says that value differs by condition, and selecting on it would remove part of what the account claims, for the reason 14.12 gives for not adjusting on the corresponding quantity in (iii). What is reported instead, beside the two estimates, is the distribution, over the met dyads of each route set, of the mean-level discrepancy of main text 3.5 over the contents of the entry-wave episode, a descriptive quantity and not a dyad-level estimate, and the proportion of dyads lying within 0.3 points of zero on it, since a dyad carrying no surplus at the first wave has none whose maintenance could be observed and the first estimand is uninformative there. Where that proportion exceeds a value named in the protocol before collection (Section 10), the first estimand is reported as uninformative and H5 (iv) as undecided rather than supported on that route. The floor check of 9.8, step 3a, is a rule about the floor of the three-point scale in the analogue and does not run here, where zero on this quantity is exact calibration and not a floor.

*Who enters the estimate, and on which waves.* In the primary analysis both estimands are computed on every dyad ever classified as meeting the condition, from its entry wave onward, the entry wave being its entry wave for the route set in question, defined under “Decided by route” below. Every later wave of such a dyad enters, carrying the probe episode it contains, whether or not a correction was assessable on it under 6.2 and whether or not the classification still held at that wave; no wave before the entry wave is entered retrospectively. A later change of classification is handled by recording the wave of the change and not by exclusion: the dyad stays in the met set from its entry wave, the proportion of dyads whose classification changed is reported with the wave of each change, since frequent switching would itself count against a standing configuration, and the estimates are reported again on the dyads of stable classification as a sensitivity analysis. The reason is the one 9.8c gives for the analogue. A wave on which no correction was assessable might seem no evidence that nothing was corrected, which would argue for entering only waves with an assessable correction; but path P2 predicts that corrections become rarer as felt understanding rises, and 8.1 notes that a wrong reading is commonly absorbed rather than registered where the configuration holds, so that a nil return is what the account expects there. A rule conditioning on an assessable correction would remove the dyads in which the loop operates most strongly and keep those whose perceivers are still testing their readings, selecting on a post-entry quantity the hypothesised process itself determines; the direction of the resulting bias is not known and none is claimed. The primary analysis applies no such rule. The estimate on waves with at least one assessable correction is reported beside it, with the number of waves and of dyads it removes by route, as the analysis that reads maintenance against a correction actually stated and assessable; and the number of assessable corrections per dyad is reported and entered as a covariate in a further sensitivity analysis, which is the order 14.12 and 9.8c fix for a quantity the process can move. A further sensitivity analysis omits the dyads whose second-route classification depends on prompted corrections (8.1). The order is that of 9.8c: the primary analysis stands, and where it and a sensitivity analysis disagree in direction, or in whether the interval clears the bound, the primary estimate governs the reading and the disagreement is reported. A wave without a probe episode supplies no value and is missing, not excluded; the number of such waves is reported by route. *Decided by route.* The two estimands are computed and read separately for the dyads met on the first route and for those met on the second, where met on a route means the route value recorded under 5.1 or 6.4, whatever label Section 7 then gives, each against the same comparison set, the dyads classified not met under Section 7 within the module, which enter from the wave at which Section 7 first so classified them, the module-entry wave for a dyad classified at entry, with wave counted since that entry wave. Membership of the comparison set is fixed at entry, as membership of a met set is fixed at the entry wave, and for the same reason: a later change to met is recorded with its wave and does not remove the dyad, since removal would select on a later state. A dyad belonging to both the comparison set and a met route set contributes to each from that set’s defined entry wave; both memberships are reported, with the number of such dyads and the waves on which the two overlap, and the estimate against the dyads that stayed not met throughout is reported beside the primary one as a sensitivity analysis. Dyads not classified at module entry carry the indicator of 14.3 and enter neither estimand; where Section 7 later decides for one, it enters the set that decision names with that wave as its entry wave. A dyad enters a route set at its entry wave for that route, the later of the wave at which that route’s value was first met and the wave at which it entered the module; a dyad met on both routes enters both sets and is counted once in each, and the number of such dyads is reported. Each route set and the comparison set are fitted separately, each with the within-set form of the model of main text 3.5, without the condition variable of 14.3, which is constant within a set, and at identical reference values of the covariates, taken under 14.2 from the pooled sample of the sets compared; an original observation enters at most once within each fit, and a dyad in two sets contributes to each fit the waves of that membership. Because the sets may share dyads, the uncertainty of the two changes and of their difference is estimated jointly, by resampling complete original dyads with all their memberships and refitting every set’s model in each resample; for the (iv) estimands that resampling replaces the delta method of 14.11, and the 90 per cent interval is read from it under 14.1. H5 (iv) is read on each route by the rule of 14.1, supported on a route only where both intervals meet the condition, falsified where either is falsified, and undecided otherwise; the pooled estimate across routes is reported for description and carries no reading. A route supplying fewer met dyads than the minimum named in the protocol before collection (Section 10) leaves (iv) untested for that route, which is reported as such and not as support. The two routes are not reached alike, and main text 6.3 says so: the sensitivity analysis on waves with an assessable correction draws mostly on the second route, since a responder met on the first sets few attributions to correct, and for the first route the primary estimate reads the course of a surplus that little disconfirmation has reached. *What withdrawal requires.* The consequence main text 3.4 attaches to a falsified (iv) follows on any route on which (iv) is adequately tested and falsified; a route left untested carries no consequence either way.

*The two rates.* The accuracy-demand rate of 9.9 and the cue-recycling proportion of 9.9 are proportions and carry neither of the bounds of 14.1, which are in points of the correspondence metric. Their bound is 5 percentage points, on the same footing as the others and equally a planning value of the author’s, reported again at 3 and 8. Path P2 predicts that the accuracy-demand rate falls while felt understanding rises, and R2 that the cue-recycling proportion rises; a change inside 5 percentage points falsifies the path in question, and a rise in the accuracy-demand rate of more than that counts against the account, as main text 6.1 states.

*Classification across waves.* The condition is coded cumulatively rather than afresh at each wave: material accumulates and a dyad enters the module from the first wave at which the minima of 3.5 are met over the material collected so far, whatever the route on which Section 7 then decides, because the model carries the coded rate of 5.4 alongside the condition. That is an entry requirement of this module and not a qualification of Section 7, which classifies a dyad met on the second route below that minimum. Before that wave the dyad contributes to no estimate that conditions on the condition, and the number of dyads held back in this way is reported by route, with the wave at which Section 7 first decided for each. Route 2 has no rate, so where a dyad enters the cross-lagged model the predictor is the coded rate of 5.4 for Route 1 together with a separate indicator for Route 2, and never a single continuous variable standing for both. The cross-lagged models of this position and the rate predictor use the wave-specific cumulative classification and the cumulative rate of 5.4, predictors that may change across waves; the entry-wave-fixed met status above is used by the (iv) estimands alone. The gate of 5.3 applies in the field module as it does anywhere else: a responder who does not pass it is *not decided* on Route 1 and is not entered as an extreme low-rate case. *What the module cannot do.* The condition is coded and not assigned, so the module supports no causal claim on its own; it is the arm in which the maintenance claim is put at risk, and the analogue arm is the one in which the mechanism is manipulated.

**14.14 H2, block order, carryover, and the identity of the responder.** The manipulated factor is itself persistence across occasions, so a block in which corrections are re-asserted can be expected to change how later blocks are rated. Three provisions follow. *Order.* Block order is completely counterbalanced across participants over the six permutations of the three conditions, with assignment to permutation at random and stratified on readiness. *Carryover.* Order and its interaction with condition are tested before the condition effect is interpreted, and the first block alone, which carries no carryover by construction, is analysed as a between-person sensitivity analysis and reported beside the within-person contrast. Where the two disagree, the between-person result stands and the within-person one is reported as carryover-affected. *The responder.* One responder identity is held across all three blocks for a given participant, because a change of identity would supply a ground for the perceiver to revise his attributions that the design does not intend, and because the criterion of Section 7 is about one responder. Responder identity is counterbalanced across participants where more than one confederate is used, and entered as a random effect.

## 15. Three composite clinical cases

**Two restrictions on reading the later conduct.** These govern every case below and every field record. A partner who takes in a correction and then acts against it has refused a request; the coding scores what he later takes the perceiver to be and not his compliance, so conduct contrary to a correction is no evidence for either route. Where the perceiver’s own words come back, an attribution of the responder’s own can stand behind them, and 6.6 decides whether it does.

These accompany Section 4 of the main text. They are composite, not single clinical encounters and not from identifiable treatment records: each synthesises patterns seen across several cases, with identifying detail altered, combined or invented. They illustrate how the criterion falls out in practice and are not evidence for it; the coding itself is worked through on summaries in Section 12 and on transcript material in Section 12A.

**Case 1: Anxious-preoccupied readiness, first route.** A perceiver in her mid-thirties, long unseen at work and at home, meets a partner who asks early what matters most to her and returns those terms for weeks as shared depth (3.2.1.a). His remark about never having managed to stay anywhere long becomes the centre of the relationship, and nothing further is said of it (3.2.1.b). As his attention grows less predictable her certainty that he sees her grows with it (3.2.2.a). To her he talks at length about what his sister’s illness has taken out of her and how a friend is bearing a separation, and across eight months sets almost nothing about the perceiver that could be wrong. Little of what holds her is investment: they do not live together and have no children or shared money.

**Case 2: Complex post-traumatic stress, near-silent partner, not classified.** A perceiver in his early forties, after childhood emotional neglect, is attached to a partner admired for intellectual depth who says little to him. The silences are read as thoughtfulness, the curtness as an unarticulated wound (3.2.1.c). As it stands the dyad is not classified: a partner who contributes nothing supplies nothing to score. The perceiver begins stating determinate content about his own state. What he says about being frightened rather than angered by the silences is taken up in his partner’s later attributions on two of the thirty-one assessable occasions, below the threshold of 5.4. To the perceiver that partner speaks in detail about the books he reads, which does not reach the comparison level of 5.3, so the gate is not passed and **Route 1 is *not decided*** under 5.1. No attribution of his has been explicitly corrected, so there is no triple and **Route 2 is not assessable** under 6.4. By line 5 of Section 7 the dyad is **not classified**, and it is neither placed inside the responder condition nor outside it. A reserved or autistic partner is the one such a rule stands to misclassify, and it needs validation against dyads of this shape before a decision here carries weight.

**Case 3: Codependent script, second route.** A perceiver in his late forties, whose self-worth has been organised around being needed, is eleven years into a relationship with a partner who relies on him. She mirrors his positions early (3.2.1.a), and her indeterminate statements about her own difficulties are received as privileged disclosures (3.2.1.b). Her warmth grows less predictable and his attribution of hidden depth strengthens (3.2.2.a). Twice he tells her in plain words that he is not asking to be managed when he is low. She agrees at the time; weeks later, on two unconnected occasions, she tells him in so many words that she can see he wants her to sort it out for him. Both are utterances and are coded as such: Section 13 scores what is said and never a reading of conduct. The corrections were explicit, literal and available, and the reading was re-asserted rather than carried. The therapeutic work is to let him notice the distance between what she supplies and what he fills in. No inference about treatment outcome is intended.

## 16. Glossary of units

**16.1 Purpose.** The main text, this manual and Supplementary Material S2 count in units, and several of them are counted in more than one design. The table gives each unit in the words of the position that fixes it, names that position, and states what stands in for it in the analogue and in chat material. Nothing here changes a definition; each is fixed where the table says, and this section only collects them.

**16.2 The units.**

| Term | Definition | Where fixed | In the analogue, and in chat material |
| --- | --- | --- | --- |
| **Content** | A statement by the perceiver about his own state, naming one state and what it is about, determinate enough that it could be wrong | Main text 3.1; S1 3.1 | The same. In the probe it is written and not spoken (9.1) |
| **Contribution** | One party’s uninterrupted stretch of speech or one sent message | S1 3.2 | Messages sent by the same party within sixty seconds are one contribution |
| **Turn** | The responder’s next contribution after a given perceiver contribution | S1 3.6 | The same |
| **Window** | The span of an occasion, from the contribution carrying the content to the close fixed in 3.3 | S1 3.6 | The same |
| **Occasion** | Opens at the perceiver’s contribution carrying a content, closes at the earliest of the sixth subsequent responder contribution, six hours of no exchange, or the perceiver’s next contribution carrying a content, which 3.3 calls the ordinary case | S1 3.3, assessable under 3.4 | In the analogue the segment stands in for it (14.3a). Written probe contents open no occasion |
| **Encounter** | A continuous period of contact, closed by six hours with no exchange | S1 3.2a | A continuous thread of messaging |
| **Session** | One sitting: the encounter in natural material, the experimental sitting in the analogue, a recording appointment in the coding study | S1 3.6 | As stated |
| **Episode** | One continuous interaction of at least ten minutes with marked points fixed in advance; the unit of the probe and the middle level of the models of main text 3.5 | S1 9.1 | In the analogue it is segment 7 of a condition block |
| **Condition block** | One of the three assigned conditions of 14.4, carrying seven segments | S1 14.3a | Analogue only |
| **Segment** | One occasion-equivalent inside a condition block, closed by an intervening task | S1 14.3a | Analogue only |
| **Wave** | One round of data collection in the field module, carrying recorded interaction, one probe episode and the S2 measures due at that interval | S1 14.13 | Field module only |
| **Entry wave** | For a met route set, the later of the wave at which that route’s value was first met and the wave of module entry; for the comparison set, the wave at which Section 7 first classified the dyad not met within the module | S1 14.13 | Field module only |
| **Horizon** | The number k of waves since entry over which both (iv) estimands are taken, named in the protocol before collection | S1 14.13, Section 10 | Field module only |

**16.3 How they nest.** A content is carried by a contribution and opens an occasion, whose window closes under 3.3; occasions lie inside encounters, and the rate of 5.4 has the assessable occasion as its denominator. The probe runs in episodes, which are the middle level of the models of main text 3.5, and an episode is not an occasion: its contents are written. In the analogue a condition block holds seven segments, the segment standing in for the occasion and, inside 5.3, for the encounter (14.4). In the field module a wave holds encounters and one episode.

## References

Cohen, J. (1960). A coefficient of agreement for nominal scales. *Educational and Psychological Measurement*, 20(1), 37-46.

Collins, N. L. (1996). Working models of attachment: Implications for explanation, emotion, and behavior. *Journal of Personality and Social Psychology*, 71(4), 810-832.

Hallgren, K. A. (2012). Computing inter-rater reliability for observational data: an overview and tutorial. *Tutorials in Quantitative Methods for Psychology*, 8(1), 23-34.

Ickes, W. (1993). Empathic accuracy. *Journal of Personality*, 61(4), 587-610.

Ickes, W., Stinson, L., Bissonnette, V., and Garcia, S. (1990). Naturalistic social cognition: empathic accuracy in mixed-sex dyads. *Journal of Personality and Social Psychology*, 59(4), 730-742.

Krippendorff, K. (2004). Reliability in content analysis: some common misconceptions and recommendations. *Human Communication Research*, 30(3), 411-433.

Lakens, D. (2017). Equivalence tests: A practical primer for t tests, correlations, and meta-analyses. *Social Psychological and Personality Science*, 8(4), 355-362.

Landis, J. R., and Koch, G. G. (1977). The measurement of observer agreement for categorical data. *Biometrics*, 33(1), 159-174.

Mestdagh, M., Pe, M., Pestman, W., Verdonck, S., Kuppens, P., and Tuerlinckx, F. (2018). Sidelining the mean: The relative variability index as a generic mean-corrected variability measure for bounded variables. *Psychological Methods*, 23(4), 690-707.

Montgomery, J. M., Nyhan, B., and Torres, M. (2018). How conditioning on posttreatment variables can ruin your experiment and what to do about it. *American Journal of Political Science*, 62(3), 760-775.

Overall, N. C., Fletcher, G. J. O., Simpson, J. A., and Fillo, J. (2015). Attachment insecurity, biased perceptions of romantic partners’ negative emotions, and hostile relationship behavior. *Journal of Personality and Social Psychology*, 108(5), 730-749.

Rogers, C. R. (1957). The necessary and sufficient conditions of therapeutic personality change. *Journal of Consulting Psychology*, 21(2), 95-103.

Rusbult, C. E., Martz, J. M., and Agnew, C. R. (1998). The Investment Model Scale: Measuring commitment level, satisfaction level, quality of alternatives, and investment size. *Personal Relationships*, 5(4), 357-387.

Sackett, P. R., and Yang, H. (2000). Correction for range restriction: an expanded typology. *Journal of Applied Psychology*, 85(1), 112-118.
