# Supplementary material for: The Interpersonal ELIZA Effect: a dyadic account of persistent over-attribution of understanding
Source: Front Psychol. 2026 Oct 21;17:1869060. doi: 10.3389/fpsyg.2026.1869060 (PMC13731227; doi:10.3389/fpsyg.2026.1869060)
Supplement: Supplementary file 2 [file Data_Sheet_2.docx]

# Supplementary Material S2: Candidate Item Pool for the Perceiver-Side Domains

**Manuscript:** The Interpersonal ELIZA Effect: A Dyadic Account of Persistent Over-Attribution of Understanding **Author:** Ashok Riehm **Referenced from:** Sections 3.4 and 6.4 of the main text

## S2.1 Status and use

This is a candidate item pool, not a validated instrument. After the validation of S2.10, three uses are permitted and no others: research use as a graded covariate in the designs of Section 3.4, always alongside the probe; within-person course tracking, reported beside the demonstrated-understanding term and never alone; and, for the four vignette-referenced items of S2.6 alone, the dependent variable of H3, on the condition Supplementary Material S1, Section 14.6 attaches to it.

**Standing rules, wherever the pool is used.** No norms, no cutoff, no decision rule, no bands, no interpretive ranges, and none derivable from an S2 sample; S2.11 gives the reason. No total and no difference between scores (S2.9). No screening, which is a classification act and needs a decision rule: the pool classifies no one and selects no one into or out of a sample. No name and no acronym, so that it cannot travel as a finished scale ahead of its evidence. No statement about the absent partner, every response coming from the perceiver, whose attributions this account calls uncalibrated; every item is worded as what he credits, and where an item could be read as a claim about the partner it was reworded or dropped. The two dated occasion reports O1 and O2 of S2.6 are the exception: they record events, form no score and enter no analysis in which the responder condition is a predictor.

**What carries what.** The configuration is constituted by the two conditions of Section 3.1. The responder condition is coded from interaction under Supplementary Material S1 and never from this pool; S2 data may not serve as input to that coding, since perceiver-supplied material contaminating the responder rating would make the coding circular. Perceiver readiness is graded, and the subscale of S2.5 with the threshold task of S2.2 is at present its only operationalisation, which is why its validation is a precondition of every use. The probe of Section 3.5 indexes the predicted surplus and not the configuration, since a dyad can meet the responder condition at high readiness and fail to show it. There is no level of readiness that a dyad must reach; readiness is graded throughout and is never cut. No S2 score is evidence that a dyad’s responder meets the condition of Section 3.1. The vulnerability routes of Section 4 are measured elsewhere (Brennan et al., 1998) or used descriptively and given no items (Carnes, 1997).

**Dependency and scope.** The coding study comes first: S1 fixes the specificity anchors, the rate threshold of the first route and the availability rule, so if the S1 indicators do not cohere these scores are uninterpretable. The word determinate carries the S1 standard, anchored for the respondent by the worked examples of S2.6, and S2 sets up no second, softer standard. The pool is written for intimate partnerships; no item is offered for friendships, mentoring, treatment relationships or companion systems, and the case of Section 6.2 is assessed from interaction logs rather than from items.

## S2.2 Why self-report at all

A self-report measure of a phenomenon marked by absent self-awareness is a severe psychometric limitation, and the objection is a fair one. The reply is not that the limitation is smaller than it looks, but that three quantities are at issue and each is measured where it can be measured.

**The discrepancy is never asked of the perceiver.** While the configuration operates it conceals the discrepancy from him: he experiences felt understanding and not the difference between felt and demonstrated understanding, because seeing that difference is the recalibration the configuration prevents. No item asks for it and no difference between S2 scores may be formed. It is computed from the probe, as the responder’s scored record against the perceiver’s own ratings on the shared three-point correspondence metric.

**Readiness is a disposition, and its facets stand differently to the objection.** Facet a asks about motivation and requires no insight into a bias. Facet b asks how much evidence is required before a contribution counts as understanding, which is a report of a standard and not of an error. Because that report may itself be shaped by the disposition it asks about, the standard is also read off performance, and the task is part of the pool rather than a fallback within it: eight brief dialogue extracts, four falling below the specificity threshold of S1 4.3 and four above it, pre-coded against that anchor set, each rated on a five-point scale of its own for how well the respondent thinks the speaker was understood. The scale carries five points and not the six of S2.4 because a judgement of how well someone was understood has a defensible middle, where an agreement item does not and a midpoint there would absorb the category below it; the two formats are never pooled and no score crosses them. The correspondence metric of S1 9.2 is deliberately not used here, and using it would invert the task: that metric scores an entry against a record of what the speaker actually felt, the extracts carry no such record, and a respondent applying it would score the echo extract highest, because an echo corresponds perfectly to what was said while an inference need not. The quantity wanted is the respondent’s own judgement of understanding, not a correspondence score. The anchors of S1 4.3 are not an ordinal scale of specificity: four of the six fall below the threshold and two above it. Of the eight extracts, four are built below the threshold and four above it, and the description S1 records above the threshold is expressly not decision-relevant and carries no reliability target, so a slope over anchor position would depend on the order in which the sub-threshold examples happen to be listed. Two provisional quantities are taken instead, both defined on the one distinction S1 does fix. The first is the mean rating given to the sub-threshold extracts, which is attributed understanding where the material sets no determinate attribution. The second is the difference between the mean rating of the supra-threshold and of the sub-threshold extracts, which is how far the respondent’s judgement moves with determinacy. A low threshold shows as a high first value and a small second. Whether either captures the disposition the main text calls readiness is to be established in validation and is not assumed here. A finer graded task remains possible and would require a specificity scale that S1 does not currently define, not merely a running example number. What S1 fixes is the specificity of each extract, not how far the speaker was in fact understood, and the task asks for no judgement of accuracy: the extracts carry no criterion of what the speaker actually felt, and none is implied. The quantity is the respondent’s own standard, that is how much determinacy he requires before he counts a contribution as understanding, and the anchor coding is the scale against which that standard is read. There is therefore no correct answer to the rating itself, only a correct specificity coding of the material, and the respondent is not told it. Readiness thereby has a second indicator that does not ask him to report a bias.

**The other two domains.** Current appraisal asks only for the felt side, and two further items record datable events and are scored into nothing (S2.6). Retrospective recognition measures post-hoc reappraisal, declared as that and never as a measure of the discrepancy; it is the smallest domain, seven items of thirty-three, optional, and never construct-defining.

## S2.3 Item-writing rules

1. First person, one proposition per item, no stacked negations, no technical vocabulary.
2. No item names a kind of person or a partner type. Items address contributions, occasions, and what the perceiver credits.
3. No item asks him to infer the partner’s motive, intent, strategy or willingness.
4. No item asks about the discrepancy in the current relationship.
5. No item addresses the perceiver as mistaken. A reverse-keyed item describes the partner or the past situation, never a failing of his.
6. Readiness items are valence-neutral: a perceiver who reads his partner as cold must be able to score high on readiness, and the current-appraisal domain must let him credit that partner with an exact grasp of what the coldness does to him.
7. Readiness items measure motivation and threshold, not expression, since a perceiver who wants to be understood and says little has high readiness and low expression.
8. Readiness items carry no threat or separation semantics: nothing about fear of abandonment, reassurance, or the partner’s availability.
9. Any item about the fate of something he said asks about a later, independent occasion, at least seven days afterwards, the interval fixed as in S1 6.5, never about the next turn, which is echo rather than persistence.
10. Any item touching a correction carries the availability filter of S2.6. An item that cannot carry one is not written.
11. Partner reserve, talkativeness and openness are not criteria and enter no score.

## S2.4 Response format

Six-point agreement, 1 strongly disagree to 6 strongly agree, with no neutral midpoint, which would otherwise absorb the category below.

Set apart from the six points, every item carries two further boxes, recorded separately: **X1**, no occasion of this kind arose; **X2**, an occasion arose and I cannot judge it. Neither is a scale point. Both are recorded as missing, never scored as 1, never treated as a low value, never imputed downward: a component that cannot be judged never enters as a low value, and failure to show understanding is not shown failure to understand. The two are kept apart because the account treats them differently, X1 being an absence of material and X2 uncertainty in the perceiver, which establishes nothing either way (S2.11). Both rates are reported separately. Items marked [NJ] are those on which the boxes are expected to be used often, and there they are printed beside the item.

**Keying.** Items marked [R] are reverse-keyed and recoded as 7 minus the raw value before any score is formed. Keying is balanced within each domain wherever the content allows it, at least two reverse-keyed candidates in readiness and in current appraisal. The retrospective domain carries none: every item there describes a revision the respondent has made, and a reversal of that content would ask him to affirm that no revision occurred, which is a different quantity and not the same one scored backwards. Item selection there therefore carries an acquiescence check in its place, so that acquiescence is separable at the item-analysis stage rather than confounded with the domain score; an acquiescence index is formed from the balanced domains, where reverse-keyed items exist, and carried as an observed covariate. A latent acquiescence factor is not modelled in the retrospective domain: with every item keyed in the same direction it would not be identified separately from the domain factor, and the model would not estimate.

**Pronouns.** Partner pronouns are set from one question at the start of the administration and substituted throughout; the item text is otherwise fixed. The pool is printed here with a neutral placeholder and the article uses the generic form.

## S2.5 Domain A: readiness

Readiness is the motivation to be understood together with the weight given to small signs of attentiveness, that is, the threshold at which an ambiguous contribution is treated as evidence of having been understood. Both facets are required: motivation alone would not support H1, because the threshold facet is what separates readiness from attachment anxiety. The score is graded and stays graded, a continuous covariate in the designs of Section 3.4, not to be dichotomised, tertile-split, banded or categorised.

Instruction: “These questions are about close intimate relationships, including your present one if you are in one. Answer for how things generally are for you.”

*Facet a, motivation to be understood*

- A1. Being understood accurately by a partner matters more to me than almost anything else a relationship gives me. [SV]
- A2. Having a partner who knows what I am like inside is one of the things I most want. [ECR-A]
- A3. I would rather be understood by a partner than agreed with. [SV]
- A4. It would be a real loss to me if a partner never came to know what I am actually like.
- A5. Being comfortable in a relationship is not enough for me without being understood in it. [SV]
- A6 [R]. I can do without being understood by a partner as long as things run smoothly.

*Facet b, weight given to small signs of attentiveness*

- A7. A small gesture from a partner is enough for me to take it that what I was going through has been understood. [NJ]
- A8. A short message from a partner is enough to leave me sure of what has been taken in. [NJ, EMP]
- A9. When a partner goes quiet, I generally take it that more has been taken in than the words show. [NJ]
- A10. I decide early that a partner has understood me, without waiting to see more. [NFC]
- A11. A small sign of attention from a partner tells me a good deal about how well I am understood. [EMP]
- A12 [R]. I wait for a partner to say something specific enough that it could be wrong, before I conclude that I have been understood.

**How the facets enter H1.** H1 is tested on the product of the threshold facet and the responder condition, with the motivation facet, attachment anxiety (Brennan et al., 1998) and the main effects of the threshold facet and of the condition entered alongside and never pooled. The equivalence bound applies to that product term and not to the main effect, which is reported beside it as secondary. The quantity is therefore the differential contribution of the threshold facet across levels of the responder condition, and not the residual of an undifferentiated readiness score. The decision rule is fixed now and does not depend on the dimensionality result: the threshold facet and the motivation facet enter H1 separately whether or not a second-order representation is descriptively supported. No second-order factor is fitted over the two facets. One defined over exactly two first-order factors is not identified without restrictions and, with the usual restrictions, is fit-equivalent to the correlated two-factor model, so no confirmatory step could support it; the question therefore does not arise, and the hypothesis is in any case about the threshold facet and not about readiness as a whole. This is the specification stated with H1 in main text 3.4, and the two are one specification, not two.

**Retained length.** The target is fixed in advance at six items, three per facet, the shortest form that keeps both facets estimable. Retention within facet is by highest corrected item-total correlation subject to content coverage, decided in a calibration sample that contributes to no hypothesis test; parallel analysis and confirmatory testing run in independent halves.

**Content the ECR anxiety subscale does not contain.** A2 is adjacent to the anxious wish for closeness, and A1, A3 and A5 are close to the self-verification tradition; all four are flagged rather than left unflagged. All of facet b is an evidential threshold, and attachment anxiety measures worry about a partner’s feelings and availability rather than a threshold: that facet is the incremental content on which H1 rests.

## S2.6 Domain B: current appraisal

Current appraisal records what the perceiver credits his partner with now; the referent is the present partner and the tense present. No item asks about a discrepancy and none about past relationships.

Two quantities are carried and never pooled. Attributed understanding names the questionnaire quantity B1 to B8 and is validated against the felt component of the probe (main text, Section 6.4), which is the outcome in every prediction of Section 3.4 but H3, whose dependent variable is attributed depth; attributed depth, marked [D], is secondary and serves the sub-operations of Sections 3.2.1.b and 3.2.2.a. The bridge between them, that concealed interiority implies the resources to grasp, is a conjecture, and stays testable only where both are measured independently in the same administration. Depth items never substitute for the primary outcome and are never combined with it.

Instruction: “These questions are about your present partner. Answer for how things are now.”

*Attributed understanding (primary)*

- B1. My partner knows what is going on in me. [PPR]
- B2. My partner can put into words what I am feeling. [PPR]
- B3. My partner has an accurate picture of how my earlier experiences affect me now.
- B4. My partner sees sides of me I keep out of sight.
- B5. My partner grasps the things about me that I like least.
- B6. When my partner is harsh with me, I take it that they register exactly what that does to me. [NJ]
- B7 [R]. There are sides of me my partner has no picture of.
- B8 [R]. What my partner takes me to be feeling is often beside the point.

*Attributed depth (secondary)*

- B9. There is more going on inside my partner than they show. [D, 3.2.1.b, NJ]
- B10. My partner’s inner life is fuller than what they put into words. [D, 3.2.1.b, NJ]
- B11. My partner carries things inside that no one reaches. [D, 3.2.1.b, NJ]
- B12. A warm moment with my partner leaves me sure their depth was there all along, including when none of it showed. [D, 3.2.2.a, NJ]

B5 and B6 are the dark-reading items, so that the domain cannot become a positive-illusions scale by accident: B6 credits the partner with an exact grasp of something damaging, the case of Section 4.1, a reading that lowers his standing and credits him at once with registering what the perceiver could not say, and B5 does the same for content the perceiver is ashamed of. B11 is not a dark-reading item and carries attributed depth. B1 and B2 are the matched understanding anchors administered alongside the four depth items, so that the two quantities are estimable together; the pairing is at domain level and not item for item.

**The vignette adaptation.** H3 of main text 3.4 is a vignette experiment and has no present partner to ask about. B9 to B12, and with them the two matched understanding anchors B1 and B2, are therefore administered there in an adapted wording that names the responder of the vignette in place of the partner, on the same six-point format, rated after each episode of the sequence. The adaptation measures what the respondent credits a described figure with and is not a score about anyone he knows; it forms no part of the current-appraisal score, enters no validation reported in S2.10, and is exempt from the four-week rule of S2.10, point 10, which governs items about a standing relationship. Whether the adapted wording behaves as the partner-referenced items do is an open question and is reported with the experiment.

*Occasion reports, scored into nothing*

O1 and O2 record datable events rather than what the perceiver credits his partner with. O2 is the perceiver-side echo of the second route coded under S1; O1 records whether an attribution about him was set at all, at a remove that keeps it off the occasion window of S1 3.3, so it is no counterpart of the rate of S1 5.4; neither is a proxy for demonstrated understanding. They enter no domain score, and no analysis in which the responder condition is a predictor, since responder-condition content would then sit on both sides of the model. They are reported item by item and carry their own labels rather than B numbers, so that no mean over a range of B items can pick them up.

- O1. At least a week later, on an occasion separate and unconnected from the one on which I told my partner something determinate about my own state, they said something about me that could have been wrong and that I could have put right. [NJ, filter F1]
- O2. At least a week later, on a separate and unconnected occasion, my partner said something about me that fitted the corrected version rather than the one I had put right. [NJ, filter F2]

Recall of what the perceiver supplied is not scored here: O1 asks whether a proposition about him was set, which is the quantity Section 3.1 defines, so a responder who remembers perfectly and sets nothing about him is not credited by it. Both items ask about later, independent occasions and not about the reply that followed.

**Filters.** Two worked examples stand before F1, so that determinate carries the S1 standard and not an everyday one. Below: “I have been a bit off lately.” Above: “I was dreading the phone call more than the appointment.”

F1 (before O1): in the past three months, did you tell your partner something determinate about how you were feeling, determinate enough that they could have got it wrong? If no, O1 is X1.

F2 (before O2): think of a time you told your partner plainly and in so many words that they had got something about you wrong, then answer three questions. (i) Did you say it outright rather than hinting? (ii) Did you say it the way you usually reach them, the same way as other things they do take in? Name two other occasions on which they answered you to the point in that same way. (iii) Did the same subject come up again at least a week later, on an occasion unconnected with the one on which you put it right? O2 is answered only where all three are yes and two such occasions can be named. Where no correction of this kind occurred, O2 is X1; where one occurred and a gate fails, O2 is X2. An unavailable correction is not scored at all, and never as a failure.

## S2.7 Domain C: retrospective recognition

**The direction problem, to be read before the items.** Recognising a past over-attribution requires the reappraisal the configuration prevents, so endorsement rises with insight while current calibration improves. No wording repairs this, and the domain is therefore contained by four rules rather than repaired by wording. (i) A high score indexes insight, not severity and not susceptibility, and it may never be reverse-scored into an index of susceptibility. (ii) A low score is not evidence that no such history exists; it is the expected reading inside an active configuration. (iii) It measures post-hoc reappraisal, which correlates with having left the relationship, with having entered treatment, and with current relationship satisfaction, so the covariates of S2.8 are reported with every retrospective result. (iv) It is optional, administered only where a past intimate relationship exists to answer about, and its omission costs the other two domains nothing.

Instruction: “These questions are about earlier intimate relationships, not your present one. Answer for how you see those relationships now.”

- C1. Looking back, I credited a former partner with more understanding of me than I would credit them with now.
- C2. Looking back, I filled in a good deal of what I took a former partner to have understood.
- C3. In an earlier relationship I felt known in a way I would not call known today.
- C4. I now see that things I read as understanding were my own words coming back to me.
- C5. Looking back, I held to the belief that I was understood after occasions that spoke against it. [NJ]
- C6. Looking back, what I took for depth in a former partner was largely what I put there. [D]
- C7. Looking back, I now see a former partner understood me less well than I believed at the time.

C1 to C5 and C7 serve the primary quantity; C6 carries attributed depth. Both boxes of S2.4 apply in the domain form: **X1**, I have had no earlier intimate relationship I can judge this against, or no occasion of this kind arose in the relationship I am answering about; **X2**, an occasion arose and I cannot judge it. C5 is unanswerable where no disconfirming occasion occurred, and that case is X1 and never a disagreement.

Two earlier candidates were dropped rather than filtered. One asked whether a former partner had never come back to something he had been corrected about: it collects the second route by self-report about an absent partner and can carry no workable availability filter, which rule 10 requires. The other asked whether the respondent had stopped asking a former partner what he had understood: it measures the behavioural outcome of Section 6.4 rather than recognition, and is carried in S2.8 instead.

## S2.8 Covariates, scored into nothing

**Reported disclosure, perceiver-rated.** D1. My partner tells me about things that are going on in them. [NJ] This records self-disclosure as a covariate of discoverability: not a criterion, not evidence of the configuration, entering no domain score and reported separately, so that the old disclosure criterion is not reintroduced through it. It is not the coded discoverability of S1 9.7 and never substitutes for it; the two names are kept apart so that the prohibition of S2.1 stays enforceable.

**Accuracy demand, self-reported.** D2, present: “I ask my partner what they have understood of me.” D3, retrospective: “I now see that I stopped asking a former partner what they had understood of me.” These are the perceiver-side record of the behavioural outcome of Section 6.4. They belong to no domain and are reported with that outcome, never in place of the observed rate.

**Insight confounds for the retrospective domain.** V1, is the relationship you answered about ongoing or ended? V2, if ended, how many months ago? V3, are you in psychological treatment now, have you been in the past, or never? V4, relationship satisfaction, measured with a short validated scale carrying its own satisfaction anchors, or, where length forbids, the single agreement-worded item “I am satisfied with my current relationship” on the six points of S2.4, with the single-item measurement error acknowledged in every model. All four enter as covariates in every model containing the retrospective score, which is never reported without them.

## S2.9 Scoring

Scores are formed separately for each quantity listed below, each estimated from the items of that group after reverse-keying and from no others; the estimator is fixed below. The three content domains yield four scores, because readiness carries its two facets separately, and attributed depth is a secondary index beside them.

- Readiness is not scored as one value. Motivation = A1 to A6, threshold = A7 to A12, or three retained items each in the short form; the two are carried separately through scoring, missing-data handling and reliability, and no mean, sum or factor score over all twelve is formed anywhere. No second-order factor is fitted: over exactly two first-order factors it is not identified without restrictions and is fit-equivalent to the correlated two-factor model with them, so there is no confirmatory step that could support one.
- Current appraisal = B1 to B8.
- Attributed depth (secondary) = B9 to B12, reported alongside the current-appraisal score and never inside it.
- Retrospective recognition = C1 to C5 and C7. C6 carries attributed depth, is reported as a single indicator with the note that one item carries no reliability, and is added neither to this mean nor to the depth index, lying in another domain and another time window.
- O1 and O2 are reported item by item. They form no score and enter no mean.

Scores are retained to two decimals for computation and reported to one, since no reliability estimate yet supports a finer discrimination.

**Missingness is informative.** X1 and X2 rates are reported per item and per domain and analysed as outcomes in their own right, since the account predicts them to fall as attribution rises. Domain scores are model-based, by full-information maximum likelihood on the confirmatory model with expected a posteriori scores, and not means over answered items, because the answered item set differs across respondents by construction: a well-calibrated perceiver correctly answers X2 on the depth items, and a mean would then rest on a different item set for him than for the next respondent. Full-information maximum likelihood is consistent under an ignorable mechanism, that is where missingness is at random given the modelled variables (Enders and Bandalos, 2001), and that is an assumption here rather than a property of the design: X2 may depend on the very attribution tendency that is unobserved, and X1 is in part genuine inapplicability rather than an absent value on a scale. The two are therefore modelled separately, X1 as a structural skip and X2 with the covariates predicting it entered in the model, and every analysis using a domain score carries both a complete-case sensitivity analysis and a pattern-mixture comparison under a stated departure from ignorability. Neither removes the assumption; both show how far the result depends on it. As a reporting rule and not an estimation procedure, a score is reported only where answered items reach a minimum: motivation 5 of 6 and threshold 5 of 6, each judged on its own items, or 3 of 3 in each short-form facet, the two facets never pooled to reach a minimum; current appraisal 6 of 8; depth 3 of 4; retrospective 4 of 6. An unformed score is not a low score and is never replaced by a low value, by the respondent’s own lowest answer, or by any downward imputation.

**No total, and no derived difference.** There is no sum score, no mean of means, no composite, no bifactor general factor and no overall index. The reason is printed with the items so that it travels with them: a total would mix a domain whose endorsement rises with insight, the retrospective one, with domains whose endorsement does not, so the same total would be reached by a perceiver who has recalibrated and by one who has not. No difference, ratio or residual between any two S2 scores is formed either. The surplus is computed from the probe and from nowhere else; it is predicted by the account rather than constitutive of it, and no step here treats it as a criterion, a definition or a qualifying condition. A high current-appraisal score establishes no configuration, no surplus, and nothing about the partner.

## S2.10 Validation programme

The criterion is the demonstration probe of Section 3.5, the adaptation of the empathic accuracy paradigm (Ickes, 1993). Validation criteria differ by domain, and the pool is the candidate throughout. Current appraisal is validated against the felt-understanding component of the demonstration probe. Readiness is evaluated on the independent measurement grounds set out in point 1, because the association it would otherwise be validated against is the one H1 puts at risk. Nothing below has been carried out.

1. **Readiness.** Validated without reference to the association H1 predicts, on four grounds: content coverage against the definition of readiness in the main text; the two-facet structure; reliability within each facet reported separately; and convergence of the self-report threshold facet with the independently constructed task of S2.2. Each of the four carries a value fixed now, before any data are seen: content coverage judged by two independent raters against the definition in the main text, with every retained item mapped to a component of it and disagreements resolved before the sample is drawn; a two-factor solution retained by parallel analysis and confirmed in an independent half at CFI of at least .95 and RMSEA of at most .06; omega of at least .70 within each facet, reported separately and never for a pooled score; and a correlation of the self-report threshold facet with each of the two task quantities of S2.2 whose 90 per cent interval excludes zero and whose point estimate is at least .25 in absolute value, positive with the mean rating of the sub-threshold extracts and negative with the difference between the supra- and sub-threshold means, since a low threshold shows as a high first value and a small second and the opposite sign would count against the measure rather than for it. Where both hold, the task stands as the second indicator of readiness. Where one holds and the other does not, the surviving task quantity is reported descriptively and enters no readiness score, and readiness rests on the self-report facet alone, which is stated with the result. Where neither holds, the task is not used and readiness again rests on the self-report facet. None of these three outcomes makes readiness unmeasured: that verdict follows only from failure of one of the other three grounds, which are content coverage, the two-facet structure and reliability within facet. The convergence ground decides whether readiness has one indicator or two, and not whether it has any. Association with the probe discrepancy is not an eligibility requirement for a readiness score, because it is the quantity H1 puts at risk. Where the four hold, a null at Stage 2 counts against H1 and not against the measurement; where readiness is unmeasured on the three grounds set out above, H1 is reported as untested; and where the convergence ground alone fails, H1 is tested on the self-report facet and a null is reported as resting on that one indicator. Each of these outcomes is decided on these numbers and not after the result is known.
2. **Current appraisal.** To be validated against the felt-understanding component from the probe, rated on the shared three-point metric. The demonstrated component is no part of that validation, and the difference between the two is never asked of the respondent.
3. **Validation precedes test.** Stage 1 runs in an independent sample and establishes each score on the grounds set out in points 1 and 2; only a score that has passed it enters a hypothesis test at Stage 2. No analysis serves as its own validation and as the hypothesis test at once. What differs between the domains is the criterion: current appraisal is validated against the felt component of the probe, which H1 does not predict, while readiness is validated on measurement grounds alone.
4. **What a null means.** Stage 1 for current appraisal is a correlation with the felt component of at least .30, fixed in advance as a measurement convention and not revised afterwards; failure ends that domain’s use as a research proxy and does not touch the account, because the probe and not the questionnaire carries the two understanding quantities. Readiness is not separable in that way, being one of the two constitutive conditions of Section 3.1 and not measured by the probe, which is why it carries two indicators. Where both pass Stage 1 and the product term at Stage 2 falls inside the bound, H1 is falsified as stated and, where the evidence for the probe’s intended use required by main text 3.4 is in hand, the result counts against the account. Stage 1 rests on evidence that does not depend on the relationship H1 predicts: content coverage against the definition of readiness in the main text, the two-facet structure, reliability within each facet, and agreement between the self-report threshold facet and the independently constructed performance task of S2.2. Where that evidence holds and H1 then returns a null, the null counts against H1 and not against the measurement. Only where Stage 1 itself fails is the readiness condition reported as unmeasured and H1 as untested, and point 1 fixes which of those failures carries that consequence. The distinction is fixed before the data are seen, so that a disappointing result cannot afterwards be re-described as a measurement failure.
5. **The bound.** The H1 estimand is in points of the three-point metric of main text 3.5, and the bound is 0.1 points per standard deviation of the threshold facet. H1 is falsified where the 90 per cent interval falls entirely inside plus or minus that value, which is the equivalence result (Lakens, 2017), and supported where the interval lies entirely above it; every result resting on it is reported again at 0.05 and 0.15. The figure is a planning value proposed by the author, set at a third of the 0.3-point contrast bound of main text 3.4 and on the same footing as the rate threshold of S1 5.4. It is prespecified and is not revised once the data are seen; where the argument from clinical relevance that main text 3.4 leaves open is made, it is made and registered before data collection.
6. **Sample.** The sample must be fixed on power and not on the width of one interval. At n = 275 the half-width of the 90 per cent interval equals the bound, so the interval falls inside it only where the estimate lands almost exactly on zero, and the power of the equivalence test is under 1 per cent. Powering the test against a true effect of zero requires the standard error on the Fisher metric to be at most .0343 for 80 per cent power, that is about 854 usable dyads, and at most .0305 for 90 per cent, about 1,079, before covariates. Those figures are an illustrative calculation on the correlation metric under a simple-correlation model assuming a true effect of zero, which is itself an assumption and not the absence of one, and they are not a design. The quantity actually tested is not a correlation but a points-per-standard-deviation slope from the cumulative-link model of main text 3.5, and the two are related through the dispersion of the outcome: a bound of 0.1 points per standard deviation of the threshold facet is a standardised slope of 0.1 divided by the standard deviation of felt understanding in points. Three ordered points fix that standard deviation from one side only: it cannot exceed 1.0, which is reached where half the ratings fall at 0 and half at 2, and it falls towards zero as the ratings concentrate in one category. The standardised slope is therefore at least .10 and carries no upper limit the scale can supply. The figures above are computed at .10, which is the smallest value the metric admits and so the largest sample this conversion can require; any real dispersion below the maximum puts the slope above .10 and the converted requirement below those figures. How far below turns on the dispersion of felt understanding, which the scale does not fix. Two things follow, and the second is uncomfortable enough to be stated plainly rather than left for a reader to derive.

First, the figures illustrate a simplified planning scenario and are **neither a lower nor an upper bound for H1** under the multilevel ordinal model of Supplementary Material S1, 14.11. They are computed for a main effect on a correlation metric, and within that same simplification the product term H1 tests needs more than its own main effect does. That comparison holds inside the simplification and does not carry across to the model actually specified, whose requirement is settled by the simulation named below. H1 tests the product with the met indicator of Supplementary Material S1, Section 14.3, and the standard error of an interaction coefficient is inflated relative to a main effect by one over the square root of p times one minus p, where p is the proportion coded met among the classified dyads and n counts classified dyads; the not-classified indicator of S1 14.3 raises the requirement further, and the table takes no account of it. That proportion is unknown, which is itself a finding of Section 11 of this supplement, and the inflation is severe at every plausible value.

| Proportion meeting the condition | Inflation of the required n | n for 80 per cent | n for 90 per cent |
| --- | --- | --- | --- |
| .50 | 4.00 | about 3,400 | about 4,300 |
| .30 | 4.76 | about 4,100 | about 5,100 |
| .20 | 6.25 | about 5,300 | about 6,700 |

Second, a study of that size is not a study one investigator completes, and no purpose is served by implying otherwise. The honest reading is that H1 at this bound would be a multi-site undertaking on the scenario tabulated here, that the bound is the expensive part of it, and that widening the bound is a substantive decision about clinical relevance to be argued before collection rather than a concession made afterwards. What scale the specified model actually requires is not settled by this scenario. The figures in the table are themselves illustrative, resting on a simple-correlation analogue and on a true effect of zero. The binding calculation waits on the dispersion estimated in the coding study and is to be made by simulation on the model actually specified, with estimated scale scores, covariates and repeated measures, where interval estimation in a regression model brings further properties of that model into the sample-size requirement (Bonett and Wright, 2011). A statistician is to set it before data collection. What is fixed here is the point of principle: it is power that fixes the sample, not the width of one interval. Under the minima of S1 3.5 each dyad supplies fifteen assessable occasions across at least three encounters, each probe episode runs at least ten minutes, and twenty per cent of dyads are double-coded in full. A sample in the thousands, were the scenario to hold, would be a multi-site undertaking running over years. Two consequences are accepted here regardless of what the scenario turns out to require. The coding study of main text 6.4 comes first and is small, and it is what decides whether the manual is usable at all. The hypothesis tests are staged after it, H2 and H5 in the analogue first, because they need fewer dyads for longer, and the field module of S1 14.13 last. 7. **Convergent and discriminant, per domain and never on a total.** The discriminant target is readiness against ECR anxiety. The H1 test concerns the product of the threshold facet and the responder condition, with the motivation facet, ECR anxiety and both main effects accounted for, as specified in S2.5, and it is a test of one differential contribution rather than a demonstration of construct validity in full. Current appraisal is correlated with the Perceived Responsiveness and Insensitivity Scale (Crasta et al., 2021) as a convergent check and not a discriminant one, since the domain shares its measurement tradition with perceived partner responsiveness (Reis et al., 2004) and a construct cannot discriminate against its own measure. The parsimony question that leaves is settled by a prespecified incremental test: current appraisal must add explained variance to the felt component over PRIS entered first, with a semipartial whose 90 per cent interval excludes zero. Where it does not, the domain is dropped and PRIS used in its place. The retrospective score is correlated with both and reported descriptively, with no claim of either kind. 8. **Provenance flags.** A2 ECR-A, the anxious wish to be close to and known by a partner; A1, A3 and A5 SV, the preference for being seen as one is over being affirmed, treated in the self-verification tradition; B1 and B2 PPR; A8 and A11 EMP, sensitivity to others’ cues in empathy inventories; A10 NFC, settling early on a judgement. Unflagged items carry no overlap identified at drafting. The flags name content areas and not the wording of foreign items; at the item-analysis stage each flagged item is dropped, reworded, or retained with the shared variance removed by part correlation, and the decision recorded. 9. **Psychometrics.** Dimensionality is examined within each domain separately, by parallel analysis then confirmatory testing, never across domains, since no total exists. Reliability is reported separately for motivation, threshold, current appraisal, attributed depth and retrospective recognition, as omega total alongside alpha, and no reliability estimate is formed for a pooled readiness score. Retest for current appraisal runs over one to two weeks in a subsample selected for no intervening change of relevance; the four-week interval is reserved for course tracking, where movement is the quantity of interest and no reliability reading is taken from it. Readiness is retested over four weeks, being a disposition. O1 and O2 enter no retest estimate, recording occasions rather than a standing appraisal. Invariance across culture and gender is untested and is a precondition of any group comparison. The threshold task of S2.2 carries its own estimates, reported before either of its quantities is used in H1: internal consistency across the four sub-threshold extracts and across the four supra-threshold extracts separately, and the reliability of the difference between the two means, computed from those two estimates and their correlation. Where the reliability of the difference falls below .60 it is reported and the difference is not used as an indicator, and H1 then rests on the self-report facet alone and says so. 10. **Re-administration.** Current appraisal not oftener than every four weeks, except for the retest subsample of point 9, which is retested at one to two weeks by design and contributes to no course-tracking estimate, and except for the vignette adaptation of S2.6, which refers to no standing relationship; readiness at intake and at the end of a course, the retrospective domain at most once per wave.

## S2.11 Misuse

This section sits with the items rather than in the article, because a caveat in a discussion section does not travel with a scale.

Sparse determinate content is explained equally well by depressive disengagement, by neurodivergent calibration of self-disclosure, and by culturally formed reserve, and the selectivity demarcation proposed in Section 5.4 is a rule to be tested rather than relied on. This pool must not be used to formulate a relationship in which determinate content is sparse for any of those reasons, and no score licenses such a formulation. The responder condition is entertained and never established from self-report.

Uncertainty about a partner settles nothing either way. No item scores doubt or confusion as an indicator, and no scoring note reads low certainty as exclusion. A perceiver who remains appropriately unsure can have a partner who meets the responder condition, and this pool cannot tell.

The reason no cutoff can be derived from an S2 sample is arithmetic rather than cautionary: the prevalence of the responder condition is unknown, and so are the sensitivity and specificity of the coding procedure of Section 3.1. Predictive values turn on all three and cannot be estimated (Meehl and Rosen, 1955).

**Administration.** Where there is violence in the relationship, the retrospective domain is not administered and no result from this pool is put to the respondent as a reality test. Items B3 and B6 can open material the setting must be able to hold; the pool is administered where that is the case and not otherwise.

Attachment strength and attribution calibration are separate quantities, and this pool measures neither the bond nor its loosening. Nor is it the primary outcome measure of a treatment trial: the outcome specified in Section 6.4 is behavioural, and monitoring here is a course-tracking adjunct. A narrowing of the probe discrepancy may reflect improved demonstrated understanding rather than recalibration by the perceiver, and a fall in the current-appraisal score on its own establishes neither, since that score records only what the partner is credited with. An S2 change score is therefore uninterpretable without the demonstrated-understanding term reported alongside it.

## S2.12 What has not been done

No item analysis has been carried out, so item difficulties, item-total correlations and distributional properties are unknown and the reduction of readiness to six items awaits them. The threshold task has not been piloted and its extracts have not been coded. Dimensionality is untested, the three domains being content domains and not established factors. Reliability is unestimated, there are no norms and no reference distributions, and invariance across culture, gender and language is untested. Acquiescence and the effect of the missing midpoint are unexamined, reading level has not been assessed, and no translation has been prepared. The pool has not been administered to anyone.

## References cited in this supplement

Bonett, D. G., and Wright, T. A. (2011). Sample size requirements for multiple regression interval estimation. *Journal of Organizational Behavior*, 32(6), 822-830.

Brennan, K. A., Clark, C. L., and Shaver, P. R. (1998). Self-report measurement of adult attachment: An integrative overview. In J. A. Simpson and W. S. Rholes (Eds.), *Attachment Theory and Close Relationships* (pp. 46-76). New York: Guilford Press.

Carnes, P. J. (1997). *The Betrayal Bond: Breaking Free of Exploitive Relationships*. Deerfield Beach: Health Communications.

Crasta, D., Rogge, R. D., Maniaci, M. R., and Reis, H. T. (2021). Toward an optimized measure of perceived partner responsiveness: Development and validation of the Perceived Responsiveness and Insensitivity Scale. *Psychological Assessment*, 33(4), 338-355.

Enders, C. K., and Bandalos, D. L. (2001). The relative performance of full information maximum likelihood estimation for missing data in structural equation models. *Structural Equation Modeling*, 8(3), 430-457.

Ickes, W. (1993). Empathic accuracy. *Journal of Personality*, 61(4), 587-610.

Lakens, D. (2017). Equivalence tests: A practical primer for t tests, correlations, and meta-analyses. *Social Psychological and Personality Science*, 8(4), 355-362.

Meehl, P. E., and Rosen, A. (1955). Antecedent probability and the efficiency of psychometric signs, patterns, or cutting scores. *Psychological Bulletin*, 52(3), 194-216.

Reis, H. T., Clark, M. S., and Holmes, J. G. (2004). Perceived partner responsiveness as an organizing construct in the study of intimacy and closeness. In D. J. Mashek and A. Aron (Eds.), *Handbook of Closeness and Intimacy* (pp. 201-225). Mahwah, NJ: Lawrence Erlbaum Associates.
